# Farming practices exert selection pressures on the resistome of natural populations of house mice

**DOI:** 10.1038/s41467-026-76403-9

**Published:** 2026-08-05

**Authors:** Morgane Gicquel, Aimara Planillo, Emanuel Heitlinger, Sofia K. Forslund-Startceva, Stephanie Kramer-Schadt, Susana C. M. Ferreira, Víctor Hugo Jarquín-Díaz

**Affiliations:** 1 Department of Ecological Dynamics, Leibniz Institute for Zoo and Wildlife Research (IZW), Berlin, Germany; 2 Department of Conservation Biology and Global Change, Doñana Biological Station (EBD-CSIC), Seville, Spain; 3 Institute for Biology. Department of Molecular Parasitology, Humboldt University Berlin (HU), Berlin, Germany; 4 Federal State Agency for Consumer & Health Protection Rhineland-Palatinate, Koblenz, Germany; 5 Max-Delbrück-Center for Molecular Medicine in the Helmholtz Association (MDC), Berlin, Germany; 6 Experimental and Clinical Research Center, Charité – Universitätsmedizin Berlin, corporate member of Freie Universität Berlin and 22 Humboldt-Universität zu Berlin, Berlin, Germany; 7 Experimental and Clinical Research Center, a cooperation between the Max-Delbrück-Center 26 for Molecular Medicine in the Helmholtz Association and the Charité - Universitätsmedizin, Berlin, Germany; 8 German Center for Cardiovascular Research (DZHK) Partner Site Berlin, Berlin, Germany; 9 DFG Excellence Cluster ImmunoPreCept, Berlin, Germany; 10 Institute of Ecology, Technische Universität Berlin, Berlin, Germany; 11 Research Institute of Wildlife Ecology, University of Veterinary Medicine Vienna, Vienna, Austria

**Keywords:** Microbiome, Antimicrobial resistance

## Abstract

The factors maintaining antimicrobial resistance genes (ARGs) in non-domesticated animal microbiomes remain unclear for species inhabiting human-dominated or less human-impacted landscapes. We analysed 875 gut metagenomes from natural populations of house mice (*Mus musculus*) on German farms between 2016 and 2022 to identify environmental and host determinants of ARG occurrence. Using joint species distribution models, we quantified the influence of landscape, climate and mouse associated characteristics on the occurrence of individual ARGs and on trait dependence among genes. Environmental variables and livestock farming intensity explained 27% of ARG variation, whereas host characteristics accounted for 8%. Analysis of ARG traits revealed that agricultural land use and exposure to livestock increased the occurrence of potentially mobile ARGs. Pig density was strongly associated with an integron-encoded sulfonamide resistance gene (*sul1)* and genes conferring tetracycline (*tet*) and beta-lactam resistance (*cblA-1*) (posterior probability 0.75). Consistently, mouse resistomes have a distinctive resistome, but share more than 50% of ARGs with livestock manure, including widespread genes and those promoted in livestock. Here, we show that landscape conditions, particularly farming intensity, shape the distribution of specific ARGs and potentially mobile ARGs in house mice microbiomes.

## Abstract (web summary)

Whether farms influence the spread of antibiotic resistance genes to non-domesticated animals inhabiting the same environment has been an elusive question. Here, the authors analyzed >800 metagenomes from captured house mice living on farms and found that farming intensity affects the distribution of specific ARGs in their microbiomes.

## Introduction

Antimicrobial resistance (AMR) represents a major concern for global health, responsible for an estimated 5 million deaths worldwide yearly^1,2^. In response to this crisis, the World Health Organization and the Food and Agriculture Organization established the ‘Global Action Plan on Antimicrobial Resistance’ in 2015, aiming for a coordinated One Health approach including human, animal and environmental health sectors^3^. The complexity of transmission routes, origins and reservoirs within and between humans, farm animals and their shared environments poses challenges for implementing effective control strategies^4^. In particular, our understanding of how different environmental factors impact the occurrence or potential transmission of antibiotic resistance genes (ARGs) in bacterial communities associated with natural populations of non-domesticated animal hosts, hereafter referred to as wildlife, or the external environment, which both might act as AMR reservoirs, is still limited^5,6^.

The mechanisms of antimicrobial resistance are a natural phenomenon that has enabled bacteria to survive, persist and evolve within specific ecological contexts, which are determined by the dynamics of the microbial community^7,8^. The use of antimicrobial drugs in medical, agricultural and livestock production imposes additional evolutionary selection pressure on bacterial communities, driving the occurrence and transmission of ARGs^9–13^. Human activities have accelerated the horizontal spread of established ARGs to clinically relevant pathogens, even from evolutionarily distant bacteria through mobile genetic elements^14^. Latent genes represent, in counterpart, a large collection of less studied genes conferring resistance that remain understudied in environmental bacterial communities and are largely absent from databases^15^. Under antibiotic selection pressure, ARGs can also evolve from precursor genes, so-called proto-ARGs, that have other functions within bacterial genomes and do not directly confer a resistance phenotype^16–18^. Thus, the resistome of a bacterial community is constituted by the metagenomic composition of established, latent and precursor genes, all of which may directly or indirectly contribute to resistance. The resistome varies substantially between environments, which represents important implications for the emergence and dissemination of resistance.

Metagenomic screening has extensively characterised the resistome of human gut, wastewater and livestock^19–22^. Such high throughput approaches have been instrumental to identifying socio-economic, genetic and environmental factors associated with resistance potential, and the dynamics of clinically relevant ARGs from environmental reservoirs to humans^10,23–26^. However, the sporadic systematic metagenomic screening of wildlife-associated resistomes limits our understanding of their role in AMR dynamics and background levels of resistance in species able to mobilise antimicrobial resistant bacteria over large geographical distances or to maintain local dissemination^27,28^. Therefore, the use of high-throughput approaches in wildlife species becomes crucial in two directions: first, to determine the basal resistome in their microbiomes and second, to assess the impact of environmental drivers on resistance dynamics.

In human-dominated landscapes, generalist wildlife species able to move freely at the interface between natural and anthropogenic environments encounter diverse sources of ARGs, e.g. via foraging, and may act as mobile links for resistance dissemination^29–32^. While birds and large mammals have been highlighted as sentinels for resistance due to their capacity for long-distance movement, small mammals, particularly rodents, are key to understanding ARG transmission at local scales, and for distinguishing environmental effects in transmission and spread^33–35^. Rodents, such as rats (*Rattus norvegicus*, *R. rattus*) and house mice (*Mus musculus*), living near human settlements and domestic animals, as well as wild rodents that have never been exposed to antibiotics, are all known to carry ARGs and resistant bacteria in their gut^36–38^. The latter highlights both the role of pest species as carriers and disseminators of resistant bacteria and ARGs, and the widespread nature of antibiotic resistance in the ecosystem. In the context of antimicrobial resistance ecology, it is crucial to understand how bacteria carrying ARGs interact with their total environment, i.e. their direct host environment as well as the wider landscape context. Microbial communities and their functional potential, including resistance genes, are shaped by different host characteristics, e.g. different life-history strategies such as dispersal or philopatry or sex differences^39^. In this respect, it is also of importance how ecological dynamics within rodents’ gut microbiota are impacted by external environmental changes and could be used to detect, predict and even control the emergence and spread of resistance threats.

Changes in the environment, land use and landscape structure are known to drive the emergence of zoonosis, and are expected to influence wildlife and their associated microbial communities through different biotic and abiotic factors that surround them^40–42^. Especially farms with high livestock density or extensive crops are sources of bacteria carrying ARGs and pollute other environments through manure, dust moved by wind, water or even wildlife^43^. Spatially-explicit integration of environmental factors along environmental gradients in a multidimensional way provides a framework to understand how microbial communities and their resistomes respond to environmental change and which factors contribute to the composition of ARG traits like mobility^44^. Nevertheless, the linkage from environment via host to microbiome to resistome has been rarely incorporated to investigate how abiotic environmental factors in an ecosystem shape the distribution and diversity of ARGs in wild microbial communities due to the imperfect integration of ecological frameworks into microbiome research.

Here, we analysed the resistome of densely sampled natural populations of house mice (*Mus musculus*) communities across transects spanning hundreds of kilometres in Germany. Under a spatially-explicit approach, we assessed whether the composition, potential mobility and selection of ARGs within gut microbial communities are associated with anthropogenic, environmental and climatic factors, and disentangled these effects from host-specific influences. Our study provides an integrative landscape-scale perspective on the transmission routes and environmental drivers underlying ARG dynamics in mobile natural populations of non-domesticated hosts.

## Results

### Composition of the house mouse resistome and microbiome

**Fig. 1:**
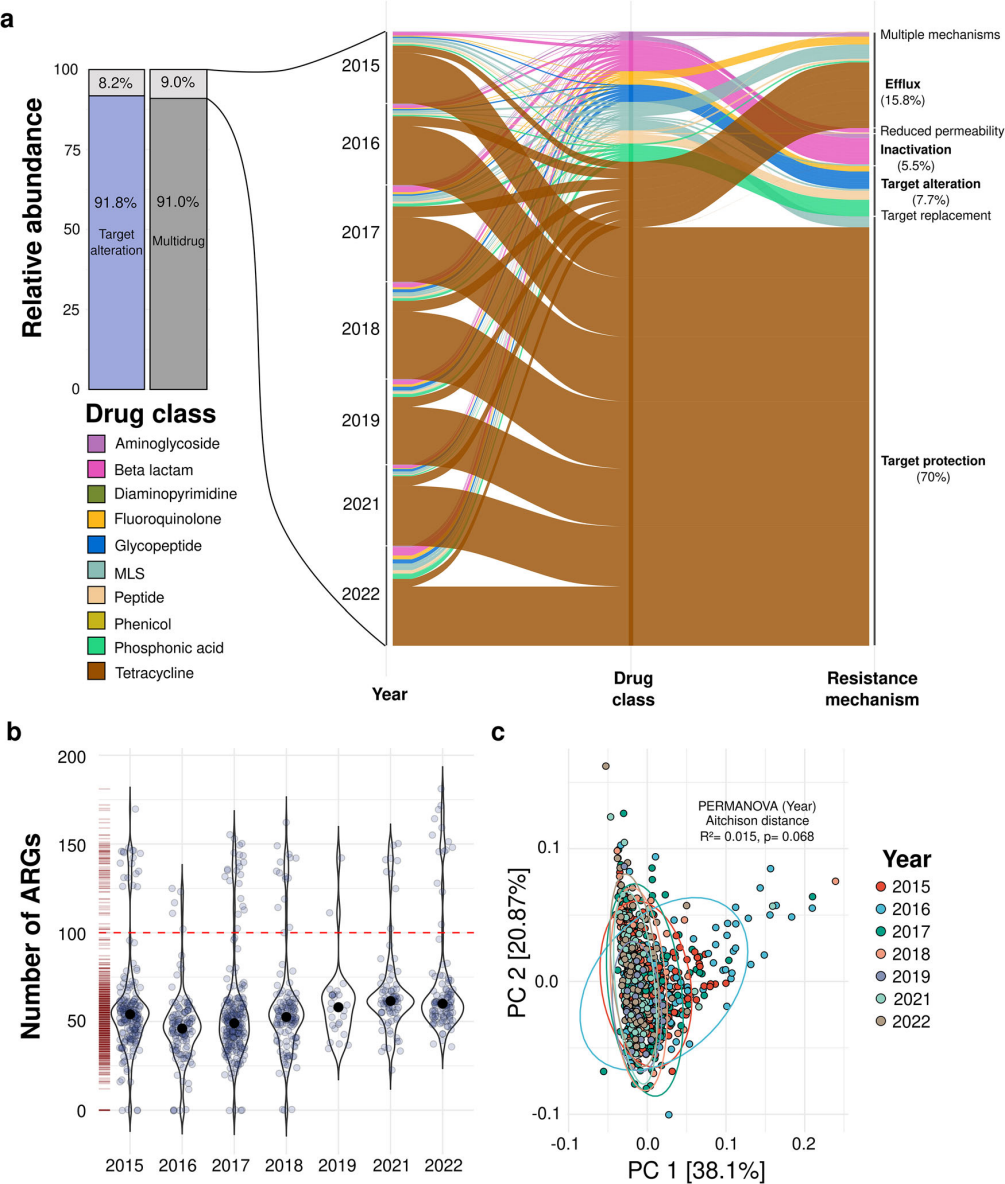
General overview of ARGs in house mouse microbiomes. **a** Average relative abundance of ARGs in all house mouse microbiomes from this study. More than 90% of the abundance was related to multidrug genes (139 genes) and target alteration genes (147 genes) as resistance mechanisms. Genes conferring resistance to the ten relevant drug classes account for only 9% of the total abundance. Of these, the majority were genes conferring resistance to beta-lactam antibiotics (46 genes), accounting for less than 1% of the total abundance. Genes conferring resistance to tetracycline (18 genes) represent 6% of the total abundance. Genes conferring resistance to tetracycline, fluoroquinolone and MLS (macrolides, lincosamides and streptogramins) antibiotics, which are related to the target protection mechanism, accounted for 70% of the abundance related exclusively to the ten major antibiotic classes. Genes with efflux, target alteration or antibiotic inactivation each represented more than 5% of the abundance related exclusively to the ten major antibiotic classes. **b** ARG richness in house mice over the years. The number of ARGs detected in 2016 was significantly lower than in other years within the same sampling campaign (Two-sided Wilcoxon rank-sum test, *p*-value Bonferroni adjusted <0.001, Table 1), and no differences were observed between years at the end of the study period. Most mice (*n* = 774) had an ARG richness of around 50 genes, while a smaller group (*n* = 85) had a higher number of genes detected. The dashed red line defines a threshold of 100 ARGs detected. Each point represents a single mouse metagenome. The black points indicate the median. **c** Principal component analysis (PCA) showing dissimilarity in ARG profiles between years of sampling. Each point represents the ARG profile of a sample. Distances between points reflect biological gene composition dissimilarity based on Aitchison distances. Points and ellipses are coloured by year of sampling.

We detected 340 ARGs in the metagenomes of 875 house mice (*Mus musculus;* hereafter: mouse). The majority of the detected genes (178) potentially confer resistance to the ten relevant antibiotic classes that are of high clinical concern. However, these genes accounted for only 9% of the total abundance. The remaining genes corresponded to non-specific multidrug genes, including mutations in housekeeping genes (e.g. rRNA) and promoter regions, and members of different multidrug efflux pump families, accounting for up to 90% of the total gene abundance (Fig. 1a). The most prevalent resistance mechanism is antibiotic target alteration (147 genes), which included 79 multidrug genes and 14 genes associated with other xenobiotics, such as disinfectants and antiseptics. Among the genes conferring resistance to relevant drug classes, those related to the target protection resistance mechanism for tetracyclines, fluoroquinolones and MLS (macrolides, lincosamides and streptogramins) antibiotics accounted for 70% of the abundance and were distributed across all years. Only 16% of the genes (55 genes) had prevalence higher than 50% and abundance above 2.5E6 Fragments Per Kilobase of gene per Million mapped reads (FPKM) across mouse metagenomes, including five conferring resistance to tetracycline and one to beta-lactam antibiotics (Supplementary Fig. 1). About 23.5% (80 genes) were potentially associated with mobile genetic elements (MGEs). Most of the genes (260) were located on the main bacterial chromosome and correspond to multidrug or mutations in housekeeping genes associated with *Escherichia coli* variants. On average, each mouse metagenome contained 59.6 genes (Fig. 1b), with few mice showing ARG richness higher than 100, which remained consistent over the years. During the sampling period of this study, we observed a significant increase in the number of genes detected towards the end; particularly, those from the last two years showed a strong increase. While temporal differences could reflect small differences in sequencing depth between sampling periods (Two-sided Wilcoxon rank-sum test, effect size *r* = 0.096, *p*-value = 4.59E-03, Supplementary Table 1) that could be attributed to the different DNA extraction kits, this change did not impact the microbial richness and the increase in ARGs was detected nonetheless after rarefying to even sequencing depth (Supplementary Fig. 2). Those mice from 2016 had lower richness (Two-sided Wilcoxon rank-sum test, effect size *r* > 0.2, *p*-value Bonferroni adjusted <0.01, Table 1) within the same sampling period. ARG composition was highly similar between mice; year explained only 2% of the variance, and the composition remained consistent over time (PERMANOVA, *p* > 0.05; Fig. 1c).

**Table 1:** Differences in ARG richness in house mice at different years

Year 1	Year 2	N 1	N 2	*P*-value adj.	Significance	Effect size
2015	2016	191	127	0.0002436	***	0.25
2015	2021	191	84	9.56E-05	****	0.28
2015	2022	191	104	2.84E-05	****	0.28
2016	2018	127	126	0.005061	**	0.23
2016	2019	127	26	0.011865	*	0.28
2016	2021	127	84	3.70E-12	****	0.51
2016	2022	127	104	8.55E-15	****	0.54
2017	2021	217	84	7.25E-08	****	0.34
2017	2022	217	104	4.10E-10	****	0.37
2018	2021	126	84	0.0003318	***	0.30
2018	2022	126	104	0.00013713	***	0.30

Only significant comparisons are shown. Two-sided Wilcoxon rank-sum test. *P* adjusted after Bonferroni correction.

We identified ARGs in 22,113 assembled contigs with >1000 bases of length. Out of them, 5874 contained genes mapped with ≥80% identity and ≥80% coverage. The majority of the ARG-carrier contigs correspond to bacterial main chromosomes (*N* = 5687) and 235 to plasmids (Supplementary Fig. 3a). In total, 28 different genes co-localised at plasmids, including genes associated with aminoglycoside (*n* = 8), glycopeptide (*n* = 8) and tetracycline (*n* = 5) resistance, with a mean abundance of 15 FPKM across the plasmids (Supplementary Fig. 3b).

A total of 606 bacterial mOTUs belonging to 50 different families were detected in the house mouse microbiomes. Each metagenomic sample contained an average of 212 mOTUs (range: 25–374), and the composition remained homogeneous over the years, sexes, geographical locations and levels of ARGs detected in the samples (PERMANOVA, *p* > 0.05). Members of the *Lachnospiraceae* and *Muribaculaceae* families were highly prevalent (prevalence > 98%) and represented the majority of abundance, at 25.8% [95% CI: 24.7–26.8] and 10.7% [95% CI: 9.99–11.5], respectively. Other bacterial families that could potentially mediate ARG transmission, such as *Enterobacteriaceae* and *Enterococcaceae*, had a prevalence below 50%, or were not detected, e.g. *Staphylococcaceae*. However, *Enterobacteriaceae* (Spearman’s correlation, Rho = 0.54, *p* = 7.3E-8), and particularly *Escherichia coli* (Spearman’s correlation, Rho = 0.71, *p* = 2.2E-16) abundance showed a strong correlation with the ARG richness within each mouse resistome and partially explained the difference in ARG levels in some resistomes (Supplementary Fig. 4). The genes corresponding to mutations in housekeeping genes associated with *Escherichia coli* variants (*n* = 48) characterise the resistome from mice with high ARG richness (≥100 ARGs) as they are occurring more (Two-sided Wilcoxon rank-sum test, *r* = 0.52, *p* = 2.39E-51) and are differentially abundant in these group of mice (Supplementary Fig. 5).

### Impact of environmental characteristics on the occurrence of ARG and compositional structure within house mouse microbiomes

**Fig. 2:**
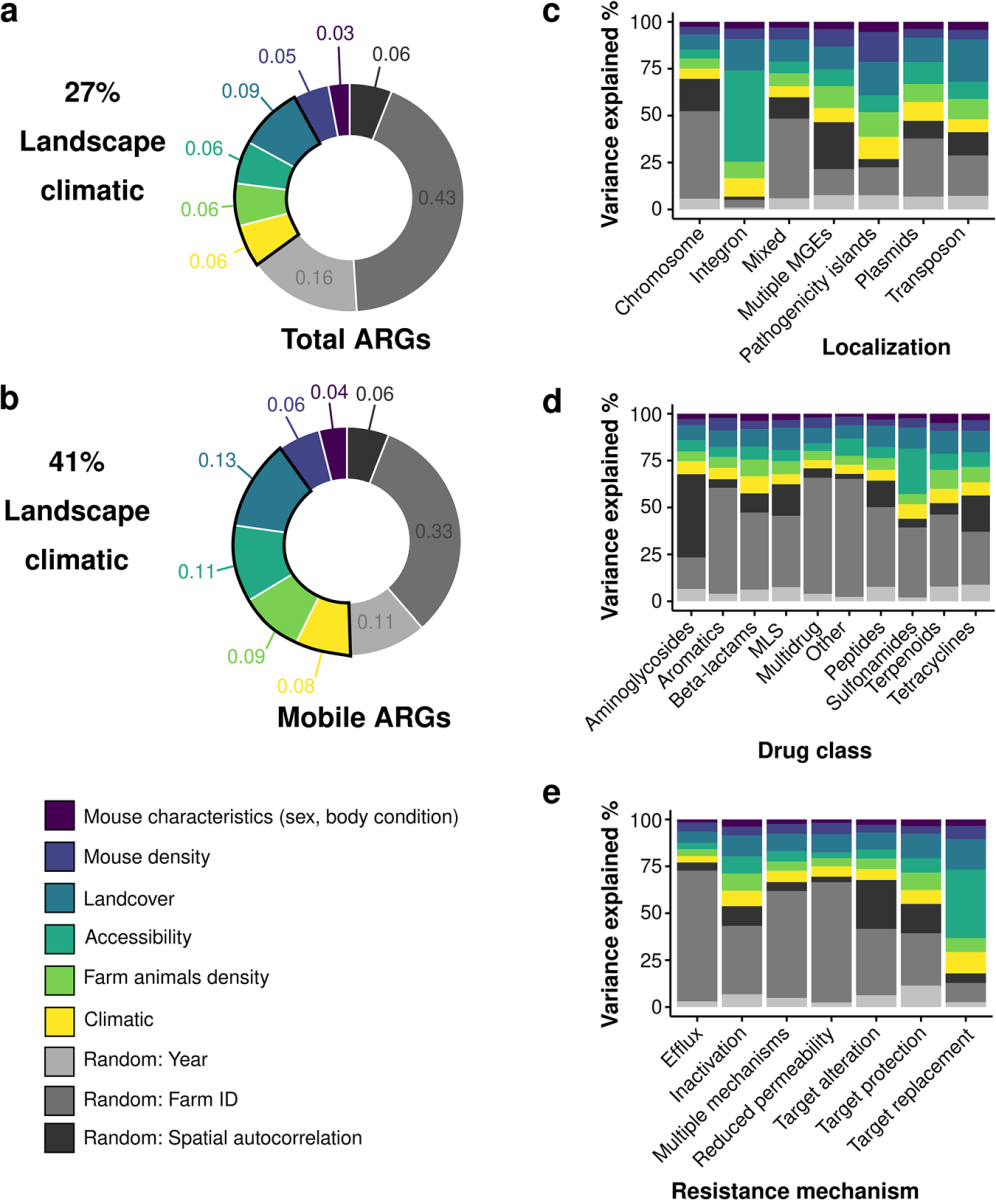
Relative contribution of mouse characteristics, environmental factors and random effects to the variation in the occurrence of ARGs in the mouse gut microbiome. **a** Variance partitioning revealed that the farm of origin and the geographical and temporal proximity between the samples (random effects) had a larger impact on the occurrence of all the detected ARGs, accounting for over 60% of the total variation. Environmental factors, including landscape characteristics and climatic factors, accounted for an additional 27%. Mouse characteristics and density explained less than 10%. **b** When these effects were evaluated only for potentially mobile genes, the environmental factors increased their explanatory contribution to 41% of the ARG occurrence, indicating that mobile genes were less dependent on specific farm characteristics. The variance partitioning averaged for each ARG’s traits (**c** Localisation, **d** Drug class and **e** Resistance mechanism) shows that the occurrence of genes with potential localisation in integrons, genes conferring resistance to sulphonamides and with target replacement mechanisms are more influenced by environmental factors.

To determine the impact of environmental characteristics associated with the farms on the occurrence (presence-absence) of ARGs in house mice microbiomes, we employed joint Species Distribution Models (jSDM) for each of the ARG responses, controlling for geographical and temporal proximity between samples and the farm identity and considering selected mouse-related characteristics, environmental factors quantify the contributions of each explanatory variable. The jSDM on ARG presence-absence had an average effective sample size of over 800, which is indicative of robust parameter estimation and low autocorrelation. Additionally, Gelman-Rubin Diagnostic parameters for beta and gamma were higher than 1, indicating adequate model convergence. Overall, the model showed strong predictive performance (AUC = 0.91), identifying samples with high and low probability of specific ARG occurrence (Supplementary Fig. 6). On average, 65% of the variance in ARG occurrence in the mouse gut bacterial microbiome was explained by random effects, and 35% by explanatory variables (Fig. 2a). The identity of each farm (Random: Farm ID) was the dominant source of explained variance, which indicates strong farm-specific effects not captured by environmental or host factors. Unmeasured ecological and farm management variables at the local level likely influenced the large variance explained by the farm. Spatial proximity between samples led to similar ARG occurrence profiles, indicating local spread or spatially similar conditions not included in our models.

Mouse-related characteristics had a low impact on the occurrence of ARG. Mouse density, represented by the number of mice trapped at the farm site, explained 5%, while mouse condition and sex explained only 3%. Among the environmental parameters, land cover represented by agricultural lands, tree cover, small woody features and impervious surfaces explained about 9%. Farm accessibility, together with farm animal density (poultry, pig, cattle) and climatic variables, explained an additional 6%.

We used the reported localisation in the Comprehensive Antibiotic Resistance Database (CARD) as a proxy of ARG mobility and refer to it as potential mobility. Thus, when the model predicted the occurrence of only ARGs in any mobile genetic element, mouse characteristics and environmental factors explained more (51%) than the model for all genes combined (35%). Land cover, accessibility, farm-related variables and climate particularly had greater explanatory power for mobile genes, showing a potentially stronger link to management practices and dispersal pathways by explaining 41% together (Fig. 2b). Potentially mobile ARGs were less tied to specific farms (33% vs. 43% for all genes) and more responsive to environmental characteristics, indicating greater potential for spread.

Ecological and environmental factors explained a large portion of ARGs trait variance, especially for those potentially in mobile genetic elements, like plasmids, integrons and transposons (Fig. 2c). The spatial proximity was the most influential factor in drug class differences, especially for genes related to resistance to aminoglycosides, tetracyclines and MLS (random: spatial autocorrelation > 20% variance explained) and indicated that they are more tied to farming practices. Genes conferring resistance to sulphonamides or beta-lactams showed a higher effect of mouse and environmental factors (>30%) (Fig. 2d). Genes corresponding to target replacement and inactivation resistance mechanisms were more influenced by environmental factors (>35%) (Fig. 2e).

**Fig. 3:**
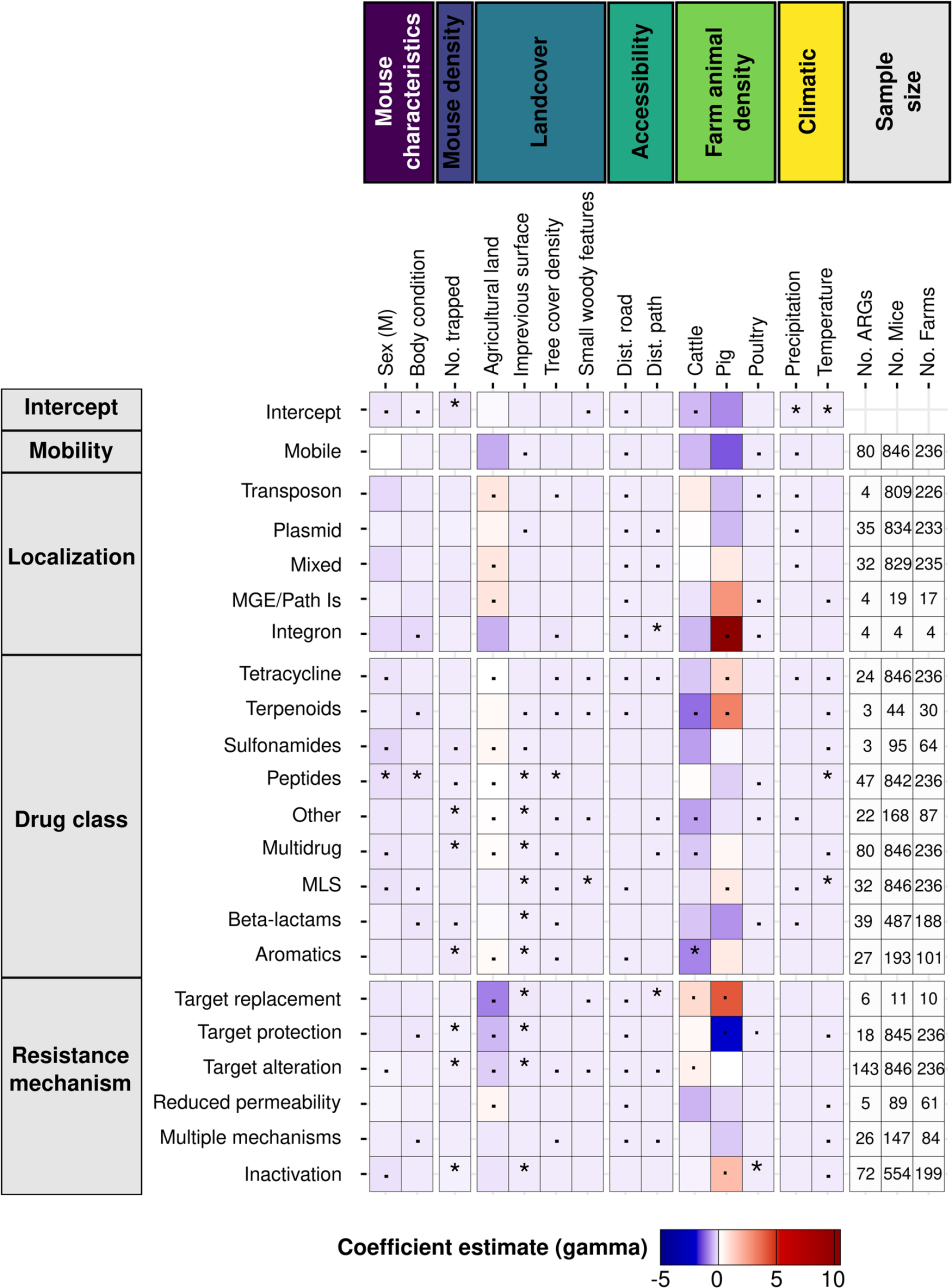
Effect of mouse-related, environmental, farm animals’ density and climatic variables on ARG occurrence agglomerated according to their traits. The heatmap shows the coefficient estimate (gamma) for the trait and a given explanatory variable in the columns (jSDM). The higher the coefficient estimate, the higher the association of the variable with the ARG trait. The intercept reference level for traits corresponds to ARGs that are non-mobile, located on chromosomes, giving resistance towards the aminoglycoside drug class, and having an efflux resistance mechanism. The number of ARGs, mice and farms is indicated in the columns from the sample size category. MLS refers to macrolides, lincosamides and streptogramins. Symbols in tile indicate the posterior probability of the estimate in the model: ∗ >0.95 and · >0.75.

To understand the influence of individual factors on the occurrence of different ARGs, we compared the gamma coefficient estimates of the jSDMs (ᵞ) to quantify the effect and directionality of each mouse-related, environmental or climatic factor on ARGs, averaged across their different traits (Fig. 3). While most of the factors included in our models had a negative association with the occurrence of ARGs at different agglomeration levels, livestock density and agricultural land were two factors strongly associated overall, although not in all cases with support higher than 75%. We observed a positive association between the surface of agricultural land and genes related to aminoglycoside, MLS and tetracycline inactivation that could be localised in mobile genetic elements. The presence of ARGs in integrons, as compared to chromosomal ARGs, was strongly and positively associated with higher pig density (ᵞ > 10, support: 0.75). The genes detected in house mice with potential co-localisation in integrons associated with pig density were *sul1*, *aadA24* and *dfrA5*. These genes are linked to conferring resistance against sulphonamide, aminoglycosides and diaminopyrimidine antibiotics, respectively. Among them, *aadA24* was detected in an assembled contig corresponding to a plasmid, providing further evidence of its mobility potential in house mice microbiomes (Supplementary Fig. 3b). The presence of ARGs conferring resistance to fluoroquinolone, phenicol and rifamycin (drug class: aromatics) showed a well-supported but low decrease with the increase in cattle density (*ᵞ* < 0, support: 0.95). Clinically and veterinary relevant ARGs conferring resistance to tetracycline were positively associated with high pig density (*ᵞ* > 0, support: 0.75), as well as other genes related to fusidane and pleuromutilin (drug class: terpenoids; *ᵞ* > 0, support: 0.75). When we focused on the impact of genes agglomerated by resistance mechanisms, a high density of pigs and cattle was positively associated with genes involved in target replacement mechanisms, including *sul* and *dfr* genes, which could also be mobile (*ᵞ* > 0, support: 0.75). While pig density was negatively associated with the occurrence of ARGs with target protection mechanisms (*ᵞ* < −5, support: 0.75), it was positively associated with genes involved in antibiotic inactivation (*ᵞ* > 5, support: 0.75). The latter included 40 genes for beta-lactamases, such as the AmpC type beta-lactamases *ampC1* and *ampH* (prevalence < 10%), as well as the *cbIA-1* gene, which is highly prevalent in mouse resistomes (prevalence > 50%). In addition to beta-lactamases, the group of genes involved in antibiotic inactivation influenced by pig density also included the *aph(3’)-IIIa* gene, which is potentially mobile and confers resistance to aminoglycosides (9% prevalence) which was also detected in assembled contigs annotated as plasmids (Supplementary Fig. 3b). Particularly, the association between *cbIA-1* and pig density was better supported when we analysed the effect on a subset of clinically relevant genes (Supplementary Fig. 7). To evaluate the effect of mice genotype, we performed the analysis for a subset of mice (*n* = 771), for which we have calculated the genotype as a hybrid index^45^ as an additional mouse characteristic. Three models, including the genotype, were evaluated: one with all the genes detected, one only with potentially mobile genes and one for clinically relevant ARGs. We observed an increase in the variance explained by mouse characteristics from 3% to 9%. The variance explained by landscape variables (land cover, accessibility and farm animals) and climatic variables compared to models without host genotype had an increase from 27% to 37% in the model for all ARGs, from 41% to 46% in the model with potentially mobile ARGs, and from 37% to 44% in clinically relevant ARGs (see Supplementary Fig. 8). These additional results suggest that the host genotype reduces the unexplained variance (random effects) and enhances the importance of environmental factors.

### Effect of environmental characteristics on the ARG compositional structure within house mice microbiomes

We employed the jSDM on the ARG abundance after filtering for rare ARGs to determine the impact of the environmental factors on the compositional structure of ARGs in house mouse microbiomes. The abundance model included 161 ARGs and similarly to the occurrence based jSDM, the diagnostic parameters indicated adequate model convergence (Supplementary Fig. 9). The variance in ARG abundance in the mouse gut bacterial microbiome was explained on average almost totally by the random effects (88.1%), and relatively few by the fixed effects (11.9%), which is a much higher proportion compared to models predicting ARG occurrence. Similar to the occurrence model, farm spatial proximity (random effect: spatial autocorrelation) was the dominant source of explained variance. This means that the spatial proximity of samples led to similar ARG abundance profiles, indicating spatially similar conditions or potential local spread not accounted for in our models. The landcover variables explained about 4%, followed by farm animal density, climatic variables and accessibility, each explaining 2%, and mouse-related characteristics each explained 1%. When focusing only on mobile ARGs, mouse-related, ecological and climatic variables explained about the same amount of variance in mobile genes (13.1%) compared to all genes (11.9%) (Supplementary Fig. 10). Considering the little variance in ARG abundance explained by these variables, we only detected a few supported effects. The temperature showed a mild negative association with ARG abundance (*ᵞ* < 0, support: 0.95). As in the occurrence-based jSDM, farm animal density did not show a well-supported relationship with ARGs in transposons and plasmids (*ᵞ* > 0, support <0.75) (Supplementary Fig. 11).

### Association of house mouse resistomes with livestock resistomes

**Fig. 4:**
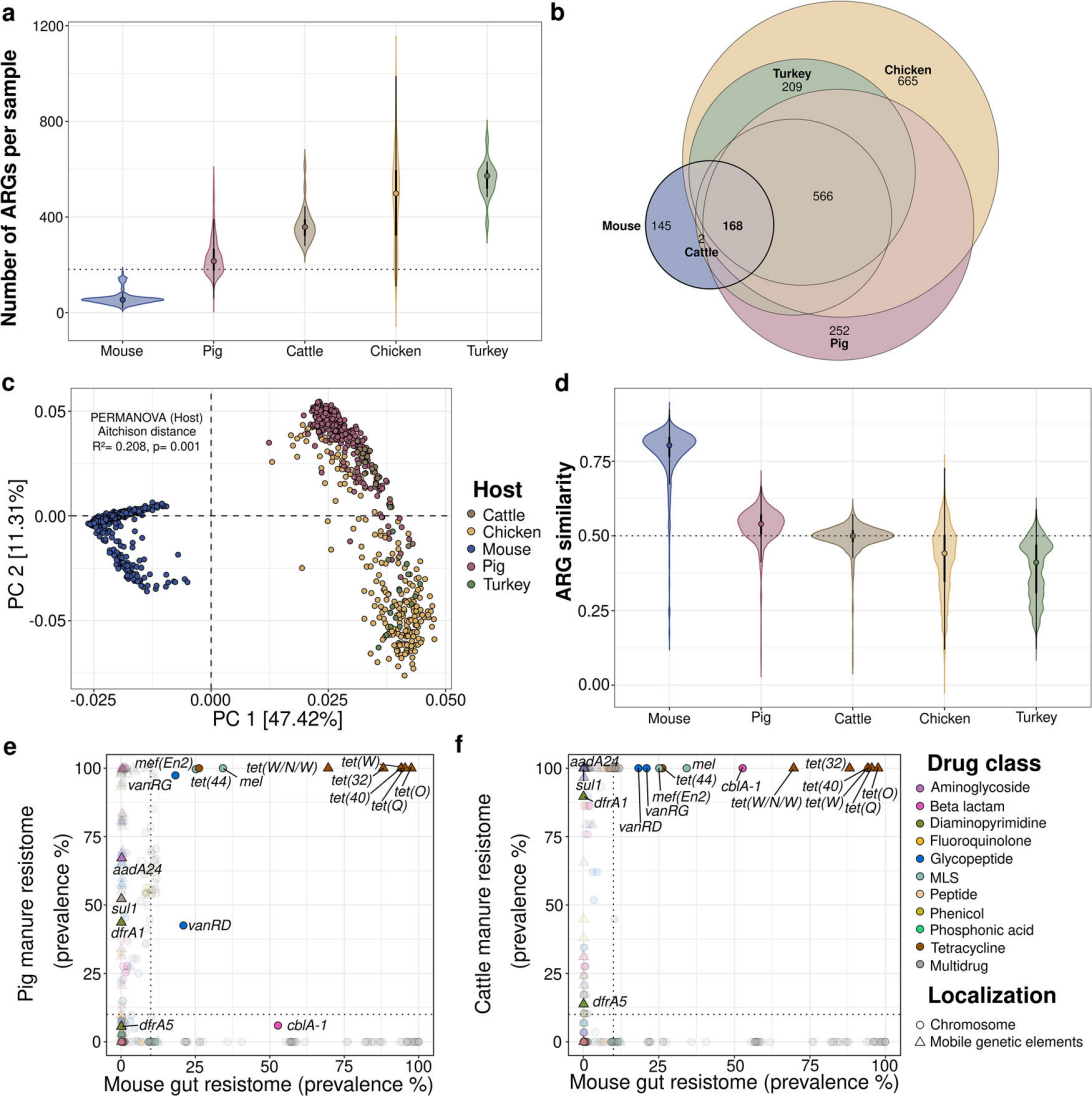
Similarities between the house mouse resistome and livestock manure ARG reservoirs. **a** ARG richness in house mice (*n* = 859) compared to livestock manure (*n*_pig_ = 268, *n*_chicken_ = 239, *n*_cattle_ = 62 and *n*_turkey_ = 70). The number of ARGs detected in house mice was significantly lower than in any other host’s manure resistome (Two-sided Wilcoxon rank-sum test, *p*-value Bonferroni adjusted <0.001, Supplementary Table 2). Poultry manure had the highest ARG richness. Pig manure’s resistomes had a smaller difference to mouse resistome richness than for the other hosts (Contrast _pig-mouse_: 173, SE [6.06]). The dashed black line defines the maximum number of ARGs detected in house mice. Each point represents the median per host. The violin represents the distribution of richness per host’s resistome. Solid boxes inside the violin indicate the interquartile range, and whiskers extend to the minimum and maximum values per host. **b** Euler diagram of shared ARGs between house mouse resistomes and different livestock manure resistomes; numbers next to the host labels indicate the number of unique genes for that particular host. A total of 168 genes were shared between the mouse resistome and the other hosts. The livestock resistomes shared 566 ARGs among them. **c** Principal component analysis (PCA) showing the dissimilarity in resistome composition among hosts. Dissimilarity is based on Aitchison distances between samples on centred log ratio-transformed ARG abundance. Each dot represents an individual resistome. **d** Comparison of ARG composition similarity between mouse resistomes and each of the other hosts’ resistomes. ARG similarity was calculated as 1 - Aitchison distance between two samples. Similarity values of 1 represent identical ARG profiles, while those equal to zero indicate completely different resistomes. The similarity values between each mouse resistome and each livestock host are presented (*n*_mouse-mouse_ = 368,511; *n*_mouse-pig_ = 230,212; *n*_mouse-chicken_ = 204,442; *n*_mouse-cattle_ = 24,911 and *n*_mouse-turkey_ = 14,603). Pig and cattle manure’s resistomes had a higher compositional similarity to mouse resistomes than to the poultry hosts (contrast _pig-mouse_: −0.264, SE [0.0098], contrast _cattle-mouse_: −0.310, SE [0.013]). The dashed black line indicates a similarity value of 0.5. Points represent the median resistome similarity between the mouse and a given host. Violin represents the distribution of similarity to mouse resistomes per host. Solid boxes inside the violin indicate the interquartile range, and whiskers extend to the minimum and maximum similarity values. **e** Prevalence of ARGs in the house mouse gut and pig manure metagenomes. While the gene *cbIA-1* had higher prevalence in mouse guts than in pig manure, indicative of house mouse promotion, three genes that can be localised in integrons (*aadA24*, *sul1* and *dfrA1*) were more prevalent in pig manure than in mice, indicating a promotion of those genes in pig manure. **f** Prevalence of ARGs in the house mouse gut and cattle manure metagenomes. The gene *cbIA-1*, prevalent in more than 50% of the mouse gut metagenomes, could be detected in all the cattle manure resistomes, similar to those genes that can be localised in integrons. For both environments, six *tet* genes had a prevalence above 75%, indicating co-promotion in such environments. In **e**, **f** labels are shown for ARGs with prevalence higher than 25% in either of the hosts and those genes that could be localised in integrons. The shape indicates whether the gene can be localised in the chromosome (circles) or in mobile genetic elements (triangles) and thus suggest mobility potential. Dashed lines indicate a cutoff at 10% prevalence to highlight genes associated with one of the hosts or in both.

Given the association of the occurrence of specific ARGs and gene traits within the house mouse resistome with the density of farm animals, we assessed the similarity between the resistomes of livestock and those of house mice in our study. We compared the resistomes of house mice to two previously published datasets: one including manure resistomes from pigs, cattle and chickens in nine European countries and the other including resistomes of rural and urban pigs and poultry (chickens and turkeys) from Ghana, as a non-European reference. We detected 2834 ARGs in total, considering 269 pig, 238 chicken, 29 cattle and 17 turkey manure metagenomic samples in addition to our 859 house mouse gut metagenomes. Overall, we observed that house mouse resistomes were less rich in ARGs than livestock resistomes (Two-sided Wilcoxon rank-sum test, effect size *r* > 0.2, *p*-value Bonferroni adjusted <0.01, Supplementary Table 2), while livestock resistomes resembled the richness detected in their original studies^25,46^. To quantify the relative effect of these differences, we fitted a linear model with ARG richness as the response. Estimated marginal means confirmed the differences between mice in the other host’s resistomes. However, the difference between the resistomes of mice and pig manure was smaller than the one observed for the other hosts (Contrast_pig-mouse_: 173, SE [6.06], Fig. 4a).

To determine the potential connectivity between house mouse resistomes and livestock manure, we calculate the number of ARGs shared between mice and each livestock host, as well as the composition similarity. Considering the total number of different ARGs detected (*N* = 2834) in the distinct hosts, the resistomes of mice shared around 50% of genes (*n* = 168) with those of livestock manure and only had a significant overlap with cattle’s manure resistome (Fisher’s exact test, *n* = 174 shared genes, *p*-value Bonferroni adjusted = 7.108813E-13, Fig. 4b). The house mouse resistome showed a distinctive ARG composition compared to the resistomes of livestock manure (PERMANOVA, *R*^2^ = 0.21, *p* = 0.001, Fig. 4c). Resistome similarity was higher between resistomes of mice and pig and cattle manure than between resistomes from poultry hosts (contrast _pig-mouse_: −0.264, SE [0.0098], contrast _cattle-mouse_: −0.310, SE [0.013], Fig. 4d).

We compared the gene prevalence from both pigs and cattle against the prevalence in house mice resistomes to determine genes that could be promoted by a specific host or in both hosts’ resistomes. Those genes present in ≥10% of both mouse and livestock host metagenomes were classified as co-promoted, while genes with prevalence ≥10% in only one of the hosts were classified as host-specific-promoted. When mouse and pig manure prevalences were compared, we observed that 11 genes were co-promoted, particularly tetracycline resistance genes (*tet*) associated with mobile genetic elements. In contrast, three genes encoded in integrons (*aadA24*, *sul1* and *dfrA1*) were promoted in pig manure, but still detected in mouse resistomes (Fig. 4e). The gene *cbIA-1* encoding a non-mobile beta-lactamase has a higher prevalence in the mouse gut than in pig manure indicative of house mouse promotion. A similar picture arose from the comparison between mouse and cattle manure, with *tet* and integron-associated genes being co-promoted, but with the difference that *cbIA-1* is also co-promoted in both cattle manure and mice, suggesting the circulation of such genes between manure and house mice (Fig. 4f).

## Discussion

Our study showed that the occurrence of antimicrobial resistance genes (ARGs) in the microbiomes of natural populations of generalist species, such as house mice, is influenced by land use characteristics. Here, we provided a framework to assess how environmental gradients influence ARG composition and the potential interactions that facilitate gene mobility among microbial communities, by conceptualising the ARGs as metacommunities within host-associated microbiomes sampled across transects within heterogeneous landscapes. We demonstrated that environmental variables related to farms, particularly livestock farming intensity, explained a higher proportion of the variation in ARG occurrence than host characteristics did. This proportion increased further when the analysis was restricted to genes' potential co-localisation in mobile genetic elements. High pig farming density was closely associated with the integron-encoded sulphonamide resistance gene *sul1*, as well as with genes conferring resistance to tetracyclines and one to beta-lactams. These findings demonstrate that specific ARGs in the resistomes of natural populations of house mice are also found in livestock manure, highlighting the exchange of resistance genes between agricultural and natural ecosystems.

Our metagenomic screening revealed that the resistomes of house mice are primarily composed of genes of limited clinical or veterinary concern, largely involved in multidrug resistance mechanisms that are ubiquitous in bacterial genomes of both host-associated and environmental origin^47,48^. This pattern is consistent with our previous 16S-based predictions of chromosomally encoded ARGs in house mice^49^ specifically, and with wild rodent resistomes in a broader sense^50^. However, we were able to detect genes potentially co-localised with mobile genetic elements. A previous study in natural populations of house mice from urban environments reported a wide distribution of ARGs conferring resistance to macrolide–lincosamide–streptogramin (MLS) antibiotics, fluoroquinolones, tetracyclines and beta-lactams^38^. While our house mouse resistomes included some of these previously reported genes and others of high priority^51^, they were detected here at low abundance and prevalence compared to other environments like the gut and livestock microbiome or wastewater systems. One explanation for the low prevalence could be the reduced abundance or absence of their typical bacterial hosts. Families of commensal gut bacteria such as *Enterobacteriaceae*, *Enterococcaceae* and *Staphylococcaceae*, which are usually involved in mediating ARG transmission within host-associated microbiomes^22,52,53^, were poorly represented in our house mouse samples as previously reported with amplicon-based approaches^39,54,55^, and mostly dominated by known fibre degraders like members of the *Lachnospiracea* family, probably related to their diet^56^. In other wild rodent species, culture-based analyses have revealed elevated levels of resistance among *Enterobacteriaceae*^36^. In contrast, wild cotton mice (*Peromyscus gossypinus*) inhabiting polluted environments with microbiomes dominated by *Desulfovibrionaceae*, *Pseudomonadaceae* and *Helicobacteraceae* showed high abundance and prevalence of multidrug resistance genes, with only a few genes associated with last resort antibiotics^57^. Conversely, wild rodents from rural areas in Germany appeared to play only a minor role in the transmission of resistant *Escherichia coli* to the environment^58^, consistent with our broader characterisation of the house mouse resistome. Together, these observations suggest that the resistomes of natural populations of house mice, similar to those of other rodents, are dominated by environmentally ubiquitous ARGs. While the natural populations of house mice are neither fully wild nor fully domestic animals, they represent an intermediate stage that gets in contact with and is influenced by both environments. Thus, the house mouse ARG composition was largely shaped by the bacterial taxa present in their microbiomes, and both bacterial and ARG composition could reflect the degree of anthropogenic exposure and potential for gene exchange across ecological boundaries.

Beyond microbial interactions within the host that may drive the selection of specific ARGs, we focused on identifying ecological drivers of the house mouse resistome. Given that both biotic and abiotic factors shape microbial communities through natural selection, and selective pressures may vary from habitat to habitat^59^, we hypothesised that rodents’ gut microbial communities and associated genetic repertoires are shaped by exposure to diverse environmental conditions. We observed that intrinsic characteristics of each farm exerted a strong influence on the overall ARG composition. Ecological and environmental variables also explained a substantial proportion of the variability in ARG occurrence. When the environmental effects are taken together, they exceed the effects of host density and sex, consistent with previous observations in the human gut resistome^19^. While our models included more environmental than host variables and therefore a higher amount of variance was expected, each subset of environmental variables (e.g. land cover, accessibility and farm animals) had a similar explanatory power to host variables with regard to the total ARG composition. This highlights the importance of the environment and its different components. Furthermore, this importance increased drastically for mobile ARGs. These patterns likely reflect anthropogenic factors that promote antibiotic resistance in bacteria within the mouse gut. Environmental and climatic factors were more influential for genes potentially co-localised on mobile genetic elements and, therefore, more likely to be transferred through horizontal gene transfer. This suggests that potentially mobile genes may be promoted by changes in agricultural intensity or land management practices, unlike chromosomal, non-mobile ARGs, which are less susceptible to external environmental pressures. In external environments such as soil and water, conventional agricultural management practices, including the use of manure as fertiliser, herbicides or compounds containing heavy metals, increase the prevalence of ARGs and mobility between bacteria by horizontal gene transfer^60–63^. We found that extensive agricultural land use was positively associated with potentially mobile genes, suggesting that those genes may be selected either through the direct impact of stress-inducing xenobiotics that reach wildlife via water and soil, or present in their microbiome through the invasion of bacteria carrying ARGs selected in the agricultural environment, as previously observed in humans^64,65^. Moreover, it has been shown that cross-species ARG transmission between livestock and wild mammals is mediated by their co-occurrence with mobile genetic elements^66^. The dissemination of ARGs between different environments, and between host-associated and environmental microbiomes, increases with the intensity of anthropogenic activities^67^. Thus, the influence of anthropogenic agricultural and farming practices is persistent on the house mouse resistome.

The use of antibiotics for food production has long been recognised as a strong contributor to antimicrobial resistance in the human microbiome, independently of clinical antibiotic consumption^68–70^. By modelling the effect of agricultural surface and livestock density on the mouse resistome, we aimed to test whether the effect of antibiotic use for food production can be quantifiable not only in humans but also in other species inhabiting human-impacted environments. Similar to agricultural practices, livestock density, particularly of pigs, was strongly associated with potentially mobile ARGs within house mouse microbes. The association between livestock density and mouse resistome can reflect two interconnected processes: dissemination of mobile ARGs through faecal pollution and epidemiological connectivity through host movement and interactions^71^. As livestock resistomes are considered hotspots of resistance genes, the dissemination of ARGs through manure occurs at a larger extent than from human, sewage and soil sources^13,72^. Pig manure is highly enriched in mobile ARGs and represents a relevant source for spreading ARGs to the environment^73–76^. The movement of mice between habitats and social interactions likely increase their exposure to sites contaminated with livestock faeces or with other rodents carrying ARGs^35,77^. Consistent with our results, other studies detected that wild rodents and even larger species such as wild boar (*Sus scrofa*) from rural areas with high livestock density in Germany, carry *E. coli* resistant to antibiotics commonly administered in pigs, poultry and cattle, suggesting livestock to wildlife transmission^43,58^. Overall, interactions between agricultural management practices, livestock density and mouse mobility may have significant impacts on the composition and mobility of resistomes in natural populations of house mouse populations.

We observed a strong association of pig density with highly prevalent genes conferring resistance to tetracycline (*tet*), which could be traced in both the house mouse resistome and in pig and cattle resistomes from Germany and other European countries. Tetracyclines have been historically used in livestock husbandry in large-scale intensive farming to prevent diseases and promote animal growth^78,79^. In Germany, tetracyclines remain among the most widely administered antibiotics, together with beta-lactams^80,81^. Despite its regulation intended to minimise selection of resistant bacteria^82^, residues of tetracyclines and their bacterial transformation products persist in livestock manure and fertilisers at concentrations that still represent an ecological risk and have antibacterial activity^83,84^. Thus, *tet* genes are persistent and highly abundant in environments with faecal or manure pollution. These genes are considered part of the cross-environment core resistome and useful for the monitoring of the total resistome diversity in the environment^15,85,86^. In our study, we detected six *tet* genes that may reflect the impact of livestock on the house mouse resistome, as they were enriched in both environments. The transmission of these genes from livestock to wildlife likely results from their co-localisation on mobile genetic elements, which facilitates efficient spread through horizontal gene transfer across bacteria^87,88^. Tetracycline ARGs are known to be promiscuous and their capacity to transfer between phylogenetically distant bacterial hosts^14^. Our results indicate that house mouse resistomes are strongly impacted by livestock density, and that mobile *tet* genes represent good markers for tracking ARG transmission between farm environments and wildlife species.

Genes encoding for commensal beta-lactamases, like *cblA-1* and other genes like *sul1* were additional markers of anthropogenic and livestock influence identified in the house mouse resistome. The *sul1* gene, although not specifically promoted in house mouse resistomes, provides interesting insights into potential ways of resistance transmission. *sul1* is a mobile and highly prevalent gene in different environments, particularly human-impacted surface waters and wastewater commonly used for agricultural irrigation^89,90^. In riverine environments, animal feeding operations have been identified as a relevant source of *sul1*^91^, highlighting how this gene may serve as a marker of connections between farming activities, agricultural practices and the dissemination to broader environments, including wildlife hosts such as house mice. We also detected that the *cblA-1* gene was highly promoted in house mouse resistomes. Although *cblA-1* is not a mobile gene and is restricted to the genus *Bacteroides*^92,93^, this *cblA-1* cephalosporinase is highly prevalent in the human gut microbiomes from Western countries and may exacerbate antimicrobial resistance infections if transferred to pathogens^94^. The presence of this gene likely reflects human or cattle faecal pollution, further supporting its role as an indicator of anthropogenic impact in the resistomes of non-domesticated animals. Taken together, genes such as *sul1* and *cblA-1* can be complementary indicators of livestock-derived and anthropogenic pollution within the house mouse resistome.

Our findings reveal that antimicrobial resistance in non-domesticated animals is not an isolated phenomenon but a measurable reflection of anthropogenic activity across agricultural landscapes. We integrated metagenomic screening and ecological data to demonstrate the impact of the external environment on the resistome of natural populations of house mice, allowing the identification of specific genes that trace this impact. The transmission dynamics between livestock, the environment and other natural populations of mammal hosts, such as house mice, can be explained by the influx of selective agents and bacteria carrying ARGs from the farming environment into surrounding habitats, where ARGs could be maintained within microbiomes of non-domesticated species. Unlike other environments, such as wastewater, polluted soil and the gut of humans and livestock, the house mouse resistome is characterised by several regulators that do not themselves confer a resistant phenotype and fewer of the high-priority genes identified by the WHO. In this respect, mice are not the most relevant carriers and are definitely not the most practical samples for surveillance purposes. However, our analysis clearly shows that their resistomes reflect anthropogenic impact, particularly with regard to genes potentially carried by mobile genetic elements. Therefore, we identified mobility as an important trait for assessing how farm animals and agricultural practices influence the resistance profiles of natural house mice populations in surrounding environments. This study broadens our understanding of the transmission of antimicrobial resistance in human-impacted environments and supports the development of strategies to monitor the mobility and dissemination of ARGs and resistant bacteria into the environment and wildlife species.

## Methods

### Study area

**Fig. 5:**
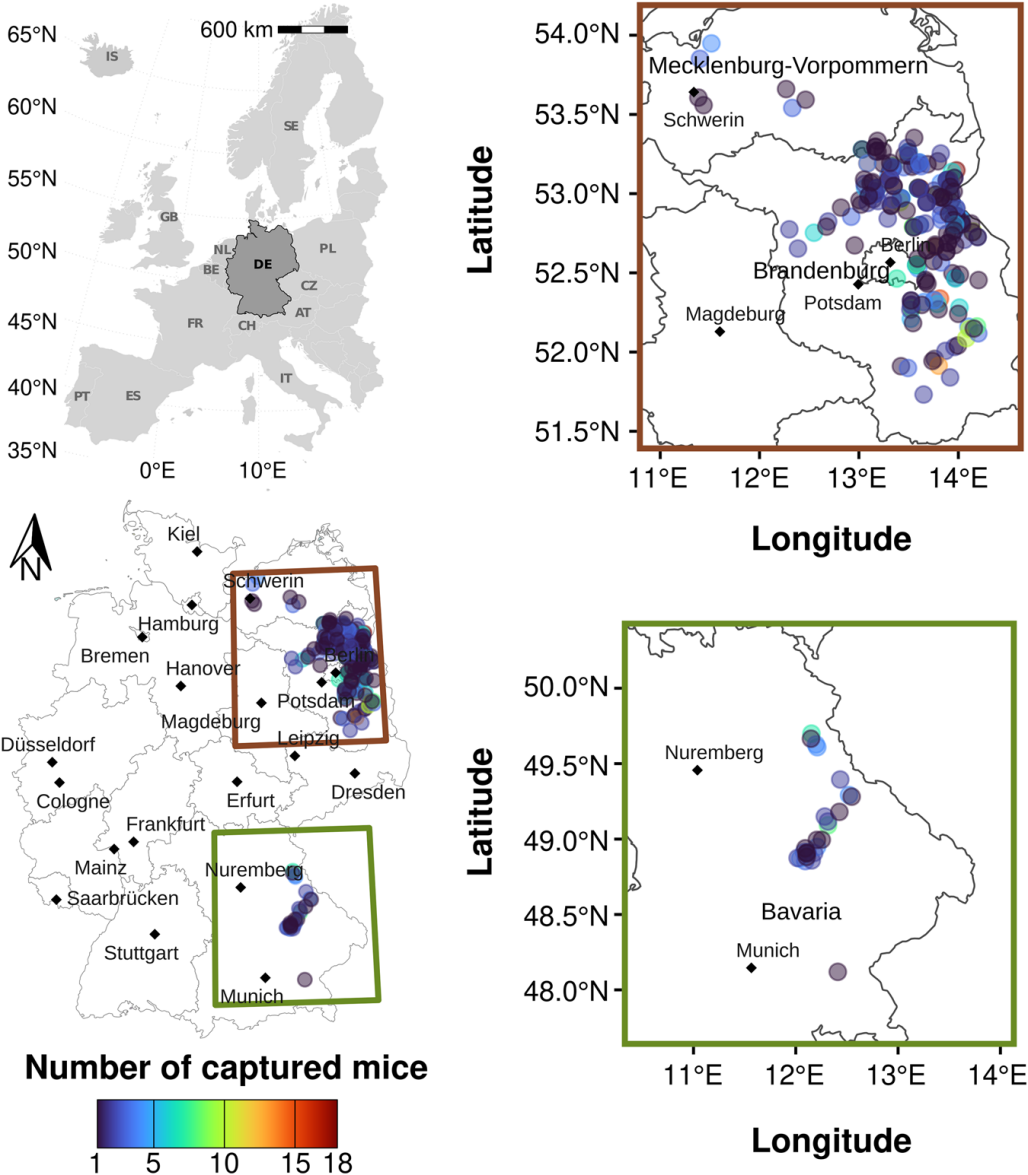
Geographical distribution of farms where mice were captured. The points indicate the geographical locations of the farms where the mice were collected. The colour indicates the number of mice captured at each location. The upper-right panel, shown with a brown border, focuses on the northern German transect, including Berlin, Brandenburg and Mecklenburg-Vorpommern and includes 802 house mice from 213 farms. The lower-right panel, shown with a green border, focuses on the southern German transect, including Bavaria, which includes 73 house mice from 27 farms. The overview maps of Europe, Germany and the German federal states were created using a public domain map dataset including vector country and administrative boundaries from Natural Earth. Made with Natural Earth. Free vector and raster map data @ naturalearthdata.com. European countries are indicated with ISO 3166-1 alpha-2 codes. Diamonds highlight the main cities in each German federal state for geographical reference.

We conducted our study in Germany, central Europe, in the federal states of Berlin, Brandenburg, Bavaria and Mecklenburg-Vorpommern (Fig. 5). Overview maps from Europe and Germany were created using a public domain map dataset including vector country and other administrative boundaries from Natural Earth. Human population density in Germany is around 241 people/km2, with 77% of the population living in urban areas (www.worldometers.info). The states selected for the study area are characterised by a large proportion of land dedicated to agriculture, from circa 70% in Mecklenburg-Vorpommern to 45% in Brandenburg and Bavaria, and 4% in Berlin (https://www.laiv-mv.de/Statistik/, https://www.statistikdaten.bayern.de/, Amt Für Statistik Berlin-Brandenburg, 2025). Forested areas occupy 24%, 37%, 36% and 18% of the state surface, respectively. Within agriculture, a large proportion is dedicated to animal husbandry, with Germany being the main European producer of milk or pork (www.bmleh.de). Crops and animal husbandry occur across the country, with different levels for each state. Poultry farming is present in all states, but it is predominant in Mecklenburg-Vorpommern. Cattle farms are found mainly in Bavaria and some areas in Brandenburg. Pig farming occurs in some locations in Bavaria and Mecklenburg-Vorpommern. In contrast, Berlin and Brandenburg produce mainly vegetables (www.bmleh.de). The study area is highly heterogeneous, with farms being in densely populated municipalities that are officially considered as urban areas according to the Federal Statistical Office. However, our main focus was on the farming practices, regardless of the official designation of the locality.

### Ethics

Animal capture and handling were conducted under the animal experimentation licences No. 2347/35/2014 and 2347/28/2021 issued by Landesamt für Arbeitsschutz, Verbraucherschutz und Gesundheit Brandenburg. Approval was provided to capture and dissect house mice from their natural environments. After capture, mice were housed individually in cages overnight and euthanised by the overdoses of isoflurane. All mice were dissected within 24 h after capture.

### Sample collection

House mice (*Mus musculus*) were captured alive from different private properties (farms) between 2015 and 2022, with only 2020 trapping missing due to COVID-19 contact restrictions. The 875 mice (479 females, 395 males) from this study represent a natural population of non-domesticated animals, which cohabitate in domestic environments. The gastrointestinal colon content was collected, flash frozen in liquid nitrogen, and later stored at −80 °C for DNA extraction at a later time^95^.

### DNA extraction and sequencing

Metagenomic DNA from colon content was extracted in two randomised batches. Samples collected between 2015 and 2018 (*N* = 679) using NucleoSpin®Soil kit (Macherey-Nagel GmbH & Co. KG, Düren, Germany) following the manufacturer’s protocol with modification as reported in previous studies^39,95^. Samples from 2019, 2021 and 2022 (*N* = 196) were extracted using the ZymoBIOMICS-96 MagBead DNA Kit (Zymo Research Europe GmbH, Freiburg, Germany) in the automatised system TECAN Fluent® 780 NAP workstation following the manufacturer’s instructions with minimum modifications to the lysis step. In brief, 750 μL ZymoBIOMICS Lysis Solution was added to ~100 mg of colon content and transferred to ZR Bashing Bead Lysis Tubes with beads (0.1 and 0.5 mm). The samples were mechanically disrupted using a PeQLab Precellys 24 (Bertin Corp., Rockville, MD, USA) for 2 × 15 s at 5500 rpm. After 5 min rest, the cycle was repeated two more times for a total of three cycles. The DNA was eluted with 50 μL of ZymoBIOMICS DNase/RNase Free Water. Aliquots with at least 750 ng quantified using Qubit fluorometric quantification dsDNA Broad Range Kit (Thermo Fisher Scientific, Waltham, USA) were shipped on dry ice for library preparation and sequencing at the Competence Centre for Genomic Analysis (Kiel, Germany). Sequencing libraries were prepared with the Illumina DNA prep kit following the manufacturer’s instructions. Shotgun sequencing was done on the Illumina NovaSeq6000 S4 platform (Illumina, San Diego, California, USA) using 300 cycles and v1.5 reagent kits.

### Bacterial taxonomy, ARG annotation, diversity and composition

Paired-end shotgun sequencing reads were assessed for quality using FastQC v0.12.1 and processed using the NGLess pipeline v1.5^96^. Briefly, quality control consisted of trimming and filtering the reads with a minimum quality of 25, a minimum length of 45 bp and removing the first 6 nucleotides. Two sequential decontamination steps were then performed. The first was to remove human contamination by filtering reads with a minimum match size of 45% and 90% identity to the reference human genome assembly GRCh38.p14. The second decontamination step aimed to remove host DNA by filtering reads mapped to the reference house mouse (*Mus musculus*) genome assembly GRCm39-mm39 using the same criteria as for the human genome. Both reference genomes were masked for low complexity regions and regions mapping to the proGenomes3 gene catalogue^97^. The taxonomic profiling of decontaminated metagenomic reads was performed with mOTUs3^98^. Samples with less than 1000 reads assigned to bacteria were removed from the analysis, and only mOTUs with an abundance above 0.005% and 1% prevalence within each metagenome were included in the analysis. ARG annotation to summarise ARG occurrence and abundance in each sample was performed using Resistance Gene Identifier (RGI bwt) v5.2.1-2. Metagenomic reads were aligned to the CARD v3.2.7^99^ using the bowtie2 aligner v2.4.5^100^ and annotated following the Antibiotic Resistance Ontology (ARO). For purposes of this study, only ARO IDs with more than 80% coverage to the reference were retained for further analysis and denoted as ARGs. ARG abundance was normalised to FPKM to control for differences in sequencing depth between samples and gene length differences. In addition, abundances were binned to ARG, AMR gene family and drug classes level. ARGs were manually regrouped based on the drugs to which they confer resistance. ARGs referring to penam, cephalosporin, carbapenem, cephamycin, penem and monobactam were grouped into the beta-lactam class. Those referring to macrolides, lincosamides and streptogramins were grouped into the MLS class. Those referring to more than one drug class were grouped into the multidrug class. The potential mobility of the ARGs detected by a read-based approach was determined based on their genetic locations with MGEs by referencing CARD Data. To determine the genetic context of ARGs, a contig-based annotation was performed. Metagenomic de novo assembly was performed with the decontaminated and quality-controlled reads using SPAdes genome assembler v4.1.0 under default parameters (k-mer = 21, 29, 39, 59, 79, 99). Only contigs longer than 1000b were used for further analysis. ARG annotation was done with RGI main using DIAMOND aligner and for plasmids using Plasmer v1.0^101^. Only ARGs in contigs with identity and coverage to the reference equal to or higher to 80% were considered. The richness of ARGs observed for each sample was calculated as the total ARG detected in the dataset for a given sample, using Microbiome v1.26.0^102^.

The bioinformatic analysis was performed on the High-Performance Computing Cluster from the Max Delbrück Centrum, Berlin (Max-Cluster), equipped with 5.2k Cores—46TB RAM.

### Retrieval of publicly available livestock manure metagenomes

To assess the proximity of house mice-derived resistomes to those from farm animals, we used two available datasets from livestock manure. One from the project EFFORT, including pooled manure from pig, cattle and poultry herds in nine European countries^25,46^. The second was from rural and urban pig and poultry farms in Ghana, which was included as a non-European reference^103^. A total of 639 faecal metagenomes were downloaded from the NCBI Sequence Read Archive (BioProjects PRJEB22062, PRJEB39685 and PRJEB62878). For these datasets, only metagenomic data from the Illumina paired-end platform with a minimum of 1E6 reads to ensure compatibility with data generated for our study and minimise inherent technical differences related to collection and sequencing between datasets. To avoid any bias in the comparisons, all metagenomes were processed and ARGs annotated in the same way as house mice-derived metagenomes (see Bacterial taxonomy and ARG annotation section).

### ARG traits and explanatory variables

We annotated detected ARGs based on four traits: (i) Potential mobility (binary) indicates whether the ARG is associated with mobile genetic elements (MGE) as reported in the CARD database (FALSE: Chromosomally encoded ARGs; TRUE: ARG associated with mobile elements such as plasmids, integrons, transposons). (ii) Localisation (categorical) indicates the type of mobile genetic element in which the gene could be located. In our dataset, localisation includes chromosome, plasmid, integron, transposon, pathogenicity island, multiple mobile genetic elements and mixed when it could be either on the bacterial chromosome or in an MGE. Due to its low representation, multiple MGEs and pathogenicity islands were grouped together. (iii) Drug class group (categorical) groups ARGs into broader drug class categories to increase statistical power due to the sparse representation of some individual classes. Grouping was based on similarity in chemical structure (Supplementary Table 3). (iv) Resistance mechanism (categorical) is a functional mechanism by which the ARG confers resistance and is classified into antibiotic target alteration, antibiotic inactivation, efflux, target protection, target replacement and reduced permeability. A subset of ARGs was analysed as clinically relevant based on the World Health Organization’s list of medically important antimicrobials^51^ (Supplementary Table 4).

We prepared a set of host-level and environmental explanatory variables hypothesised to influence the distribution of antibiotic resistance genes (ARGs) in house mice. As mouse host characteristics reflecting different life-histories, we used sex (female/male), the body condition *(BC)* of the mice calculated as the residuals of the body weight and body length relationship and a mouse density proxy, calculated as the number of trapped individuals per site (*Ntrapped*). While mouse density may influence microbial exchange or exposure risk, the body condition might reflect disturbed microbiomes either as a cause or a consequence of bad health. For a subset of mice (*N* = 771), the host genotype, represented by the genome admixture of the two mouse subspecies within the collection area, was available as a hybrid index (HI). The HI was calculated as the proportion of *Mus musculus musculus* subspecies alleles present in a set of 14 diagnostic markers, as reported in Balard et al.^39,45^.

Environmental variables included proportions of different land cover features, such as the proportion of forest or agriculture in a 100 m raster, accessibility of the farm calculated by distances to linear transport features, farm animal density describing municipality-level livestock densities for cattle, pigs and poultry, as well as climatic data, averaged for the month of August in the respective sampling year.

All spatial variables were projected to a common coordinate reference system (ETRS89/LAEA Europe, EPSG:3035, see source and original specifications of spatial layers in Supplementary Table 5), rasterised if needed and resampled to a 100 × 100 m^2^ resolution for spatial consistency. The proportion of agricultural areas was derived from the Corine Land Cover spatial layer class 2 by calculating the percentage of the land use class within each 100 × 100 m^2^ grid cell. Distances to roads and paths were calculated by rasterising the vector map to a 100 m resolution and calculating the distance to each linear road/path feature to the centre grid cell coordinates. Values from these layers were then extracted for each sample based on its geographical location. Farm animal information was obtained from the Thünen agricultural atlas as the number of livestock units per municipality^104^. We selected the three main livestock species in our study area (cattle, pigs and poultry) and transformed the number of heads of each species into densities to obtain a measure comparable across all sampling sites. The densities were obtained by dividing the number of heads by the total area of the corresponding municipality (Supplementary Fig. 12).

### Factors affecting ARG distribution and abundance

We applied two jSDM within the Bayesian framework of Hierarchical Models of Species Communities (HMSC)^105^ to model the occurrence (presence-absence) and the abundance of ARGs as a function of selected explanatory variables. Models were implemented using the *Hmsc* v 3.3-7^106^. The HMSC framework is ideal for the analysis of multiresponse data, as it allows for the inclusion of both fixed and random effects (described in the following paragraphs), as well as controlling for the spatial autocorrelation structure in the residuals, while analysing the data at the level of the taxa or the traits. Additionally, it also provides a measure of association between the different taxa after controlling for the effects of all explanatory variables, i.e., positive or negative associations after accounting for environmental preferences.

The response matrix in the model consisted of presence-absence or abundance data, respectively, for ARGs detected in individual mice sampled across multiple farms, municipalities and regions. To assess how ARG trait–host-environment interactions shaped ARG distributions, we also included a trait matrix comprising relevant ARG characteristics (see ARG traits and explanatory variables) in both models. Each of the detected ARGs in house mice resistomes could be represented in multiple traits. To model binary ARG presence-absence data, we used a probit error distribution, and to model ARG abundance, we log-transformed it (log(*x* + 1)) to fit a Gaussian distribution. For the abundance models, we first filtered out ARGs with an overall prevalence lower than or equal to 5% to avoid chain convergence problems.

After the removal of correlated explanatory variables (fixed effects), we additively included the mouse characteristics (sex, body condition, mouse density), landcover (agricultural land, impervious surface, tree cover density, small woody features), accessibility (distance to road, distance to path) farm animal density (cattle, pig and poultry) and climatic characteristics (precipitation and temperature) as explanatory variables into the model. To account for the nested and spatio-temporal structure of the study design, we specified three levels of random effects: Spatial autocorrelation was accounted for by including the XY-coordinates of farm locations as a spatially-explicit random effect. To account for local farm-level variability and nested effects of the sampling design with multiple mice per farm, we included FarmID. Lastly, we included Year as a temporal random effect to capture inter-annual variability.

### Association of house mouse and livestock manure resistomes

After preprocessing and ARG annotation, the resistomes of livestock manure and house mouse were merged into a single dataset for further analysis. The resulting dataset includes FPKM abundances for each ARG, along with the country and host of origin and ARG ontology. The combined dataset was compiled using Phyloseq v1.48.0^107^. Resistome richness and dissimilarity were estimated as described for the house mouse dataset alone (see ‘Bacterial taxonomy and ARG annotation’ section). The prevalence of each ARG in mice and livestock was estimated as the proportion of metagenomes from each host where the given gene was detected. Comparisons of host-based prevalence were used to determine the potential sources of genes between the livestock hosts and the mice. Genes were categorised as co-promoted if they were present in ≥10% of samples from both mice and a given livestock host. Genes present only in ≥10% of samples from one type of host were categorised as host-specific. ARGs present in fewer than 10% of samples from mice and a given livestock host were classified as non-promoted^108^.

### Statistics and reproducibility

All analyses were performed in R version 4.4.1 (R Core Team, 2024). Source data and code required for the analysis are available to ensure reproducibility (See ‘Data availability’ and ‘Code availability’).

For microbiome and resistome analysis, Principal component analysis and permutational multivariate analysis of variance (PERMANOVA) were performed based on centred-log-ratio transformed FPKM ARG abundances and 9999 permutations using ‘adonis2’ from package ‘vegan’ v2.6-475. PERMANOVA for both microbial and ARG profiles was run by = ‘margin’ using year of collection, mouse sex, longitude, latitude and transect as geographical references, taxonomically assigned read counts, and level of ARG count as predictors, stratifying by sequencing batch. Comparisons of ARG richness between mice with different levels of ARGs were conducted using a two-sided Mann–Whitney U-test with Bonferroni correction for multiple comparisons. The effect size r was calculated as the Z statistic of the MWU test divided by the square root of the sample size, as implemented in the package rstatix v0.7.2. Significance was determined at a *p*-value cut-off of 0.05 unless otherwise stated. All figures were created using the following packages: ggplot2 v3.5.1, ggsci v3.2.0, ggpubr v0.6.0, gridExtra v2.3 and RColorBrewer v1.1-3 with minor editions using Inkscape v0.92.5. Data handling was performed using pipelines compatible with tidyverse v2.0.0.

To reduce multicollinearity among environmental explanatory variables, we calculated pairwise Pearson correlation coefficients *r* and excluded one variable from each pair with |*r*| > 0.7, retaining the most ecologically informative predictors (Supplementary Fig. 13).

For the ARG occurrence jSDM, we ran four parallel Markov Chain Monte Carlo (MCMC) chains, with 1000 burn-in iterations, followed by 10,000 sampling iterations and a thinning interval of 10, resulting in 4000 posterior samples per parameter. For the ARG abundance jSDM model, we ran four parallel MCMC chains, with 1000 burn-in iterations, followed by 15,000 sampling iterations and a thinning interval of 10, resulting in 6000 posterior samples per parameter.

Model convergence was assessed using MCMC trace plots, Gelman-Rubin diagnostics, and effective sample sizes of a random subset of parameters. Model performance was evaluated using Tjur’s *R*², or *R*², and the area under the receiver operating characteristic curve (AUC). To quantify the contributions of the host and environmental explanatory variables versus the nested and spatio-temporal structure of the data, we decomposed *R*² into variance components associated with fixed effects and each random effect using the partitioning tools in the *Hmsc* v 3.3-7.

The relative effect of the livestock host of origin on differences in richness against the house mouse resistome was estimated by fitting a linear model with ARG richness as response and host and country as predictor variables using ‘stats’ v3.6.2. Estimated marginal means (EMMs) for each host, and pairwise comparisons between EMMs of mouse and livestock resistome richness were calculated using ‘emmeans’ v1.11.1. Fisher’s exact test was used to determine the degree of intersection in ARGs detected between the resistomes of mice and livestock. Pairwise comparisons were performed between each livestock host and the mouse resistome, and the *p*-values were adjusted using Bonferroni correction for multiple comparisons. An Euler diagram to represent ARG overlaps between the resistome of different hosts was done with ‘eulerr’ v7.0.2. To determine whether the resistome composition of mice was more similar to that of a particular livestock host, a linear mixed-effects model was fitted using ‘lme4’ v1.1-37. The model used *1 - Aitchison* distance as a measure of resistome similarity between pairs of samples. Only resistome similarities within mice and between mice and livestock hosts were used. Host pair and country pair were used as fixed effects, and the sample IDs of the pairs were included as random effects to control for pseudo-replication. As described for the richness model above, EMMs were calculated for each host pair, and the relative degree of resistome similarity was determined by calculating pairwise comparisons between EMMs within the mouse and between mouse-livestock resistomes.

### Reporting summary

Further information on research design is available in the Nature Portfolio Reporting Summary linked to this article.

## Supplementary information

Supplementary Information

Reporting Summary

Transparent Peer Review File

## Data Availability

All raw sequence data are deposited and available online at the European Nucleotide Archive under accession number PRJEB102564. Additional source data for the analysis have been deposited in Zenodo and it is available at 10.5281/zenodo.20624924. Faecal metagenomes from livestock animals were downloaded from the NCBI Sequence Read Archive BioProjects: PRJEB22062, PRJEB39685 and PRJEB62878. All run accession numbers are available at Supplementary Table 6. Data used for overview maps in Fig. 5 and Supplementary Fig. 12: Made with Natural Earth. Free vector and raster map data @ naturalearthdata.com.

## Code availability

All the code used for visualisation and statistical analysis have been deposited in Zenodo under the 10.5281/zenodo.20624924 and the EcoDyn github account https://github.com/EcoDynIZW/Gicquel_Planillo_2026_NatCommun.
