# Supplementary material for: Farming practices exert selection pressures on the resistome of natural populations of house mice
Source: Nat Commun. 2026 Aug 5;17:7848. doi: 10.1038/s41467-026-76403-9 (PMC13443167; doi:10.1038/s41467-026-76403-9)
Supplement: Supplementary file 1 [file 41467_2026_76403_MOESM1_ESM.pdf]

Farming practices exert selection pressures on the resistome of natural populations of house mice

Morgane Gicquel[1], Aimara Planillo[1,2], Emanuel Heitlinger[1,3,4], Sofia K. Forslund-Startceva[5,6,7,8,9], Stephanie Kramer-Schadt[1,10], Susana C. M. Ferreira[1,2,11], Víctor Hugo Jarquín-Díaz[5,6,7*]

[1]Department of Ecological Dynamics, Leibniz Institute for Zoo and Wildlife Research (IZW), Alfred-Kowalke-Straße 17, 10315, Berlin, Germany.

[2]Department of Conservation Biology and Global Change, Doñana Biological Station (EBD-CSIC), Seville, Spain

[3]Institute for Biology. Department of Molecular Parasitology. Humboldt University Berlin (HU). Philippstr. 13, Haus 14, 10115, Berlin, Germany.

[4]Federal State Agency for Consumer & Health Protection Rhineland-Palatinate, Koblenz, Germany.

[5]Max-Delbrück-Center for Molecular Medicine in the Helmholtz Association (MDC), Berlin, Germany.

[6]Charité – Universitätsmedizin Berlin, corporate member of Freie Universität Berlin and Humboldt-Universität zu Berlin, Experimental and Clinical Research Center, Lindenberger Weg 80, 13125 Berlin, Germany.

[7]Experimental and Clinical Research Center, a cooperation between the Max-Delbrück-Center for Molecular Medicine in the Helmholtz Association and the Charité - Universitätsmedizin Berlin, Germany.

[8]German Center for Cardiovascular Research (DZHK) partner site Berlin, Berlin, Germany.

[9]DFG Excellence Cluster ImmunoPreCept, Berlin, Germany.

[10]Institute of Ecology, Technische Universität Berlin, Rothenburgstr. 12, 12165 Berlin, Germany.

[11]University of Veterinary Medicine Vienna, Research Institute of Wildlife Ecology, Veterinärplatz 1 1210, Vienna, Austria

These authors contributed equally: Morgane Gicquel, Aimara Planillo

*Corresponding author: VictorHugo.JarquinDiaz@mdc-berlin.de

Supplementary Information

We include figures and tables that further support the results and conclusions presented in the main manuscript.

Inventory of Supplementary Information

Supplementary Figures

- Supplementary Fig. 1. Overall prevalence and abundance per ARG.
- Supplementary Fig. 2. Effect of sampling campaigns in sequencing output, taxonomy and ARGs.
- Supplementary Fig. 3. Genetic context of ARGs in contigs with more than 1000b.
- Supplementary Fig. 4. General overview of the colon microbiomes from house mice and their relationship to ARG levels.
- Supplementary Fig. 5. Mice with high ARG level (>= 100 ARGs) are characterised by *Escherichia coli* related genes.
- Supplementary Fig. 6. Convergence diagnostics for the ARG presence-absence joint species distribution model (jSDM).
- Supplementary Fig. 7. Variance partitioning showing the relative contribution of mouse characteristics, environmental factors, and random effects, on the explained variation in clinically relevant ARG occurrence in mouse gut microbiomes.
- Supplementary Fig. 8. Variance partitioning showing the relative contribution of mouse characteristics, environmental factors, and random effects, on the explained variation.
- Supplementary Fig. 9. Convergence diagnostics for the jSDM ARG abundance model.
- Supplementary Fig. 10. Variance partitioning showing the relative contribution of mouse characteristics, environmental factors, and random effects, on the explained variation in ARGs abundance in mouse gut microbiomes.
- Supplementary Fig. 11. Effect of mouse-related, environmental, farm animal density and climatic variables on ARG abundance agglomerated according to their traits.
- Supplementary Fig. 12. Maps of environmental layers in the sampled regions (Berlin-Brandenburg-Mecklenburg-Vorpommern and Bavaria).
- Supplementary Fig. 13. Pearson correlation matrix showing the pairwise linear relationships between the explanatory variables.

Supplementary Tables

- Supplementary Table 1. Effect of sampling campaigns in sequencing output, taxonomy and ARGs.
- Supplementary Table 2. Differences in ARG richness between house mice and livestock manure.
- Supplementary Table 3. Classification of antimicrobial resistance genes (ARGs).
- Supplementary Table 4. Clinically relevant groups of antimicrobial resistance genes (ARGs).
- Supplementary Table 5. Environmental layers used for modelling ARG occurrence in the mouse gut microbiome.
- Supplementary Table 6. Livestock manure sequencing runs used for comparative analysis.

Supplementary Figures

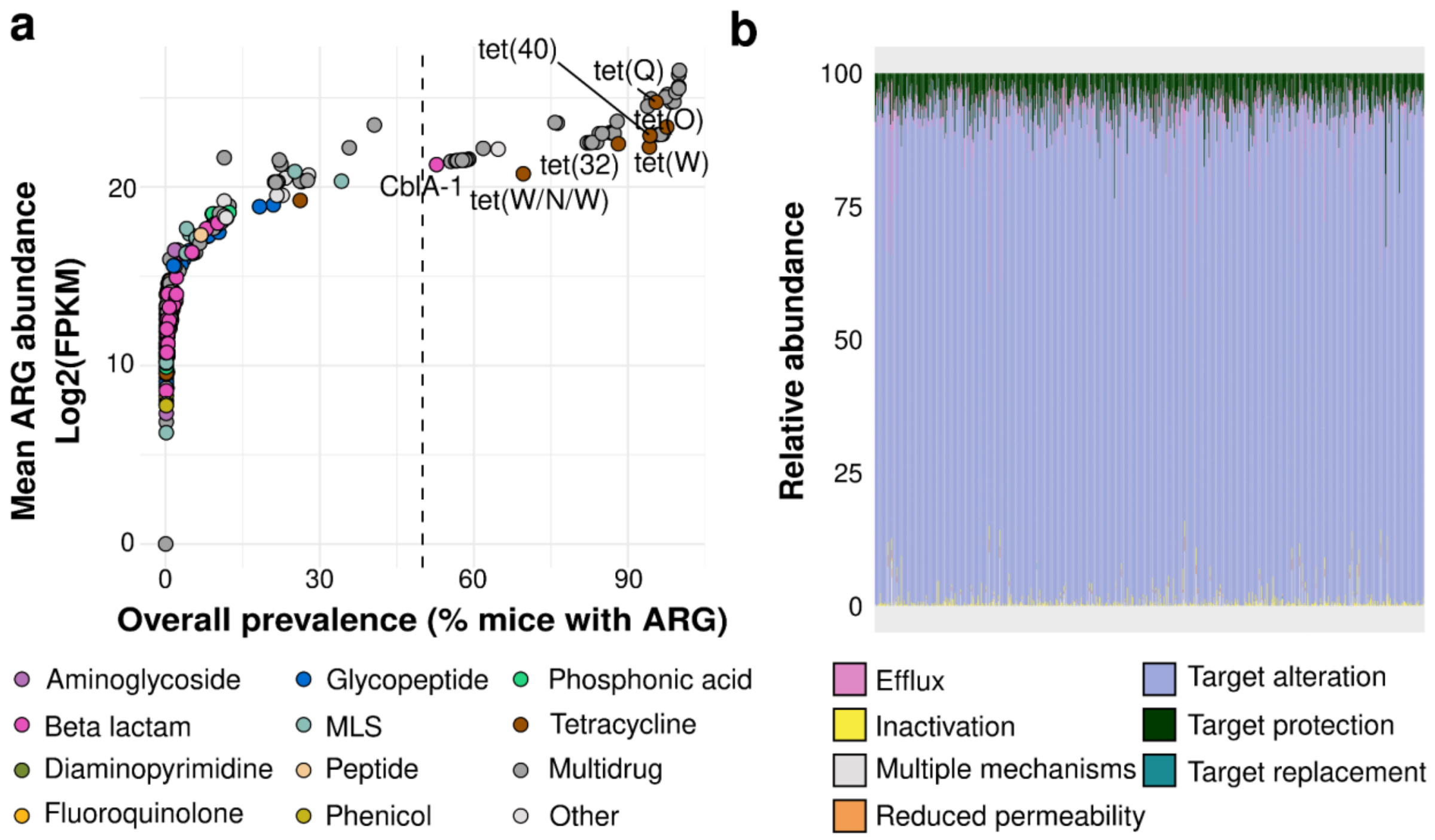

Supplementary Fig. 1. Overall prevalence and abundance per ARG. a. Mean abundance and prevalence of ARGs in the colon content metagenomes from house mice. In total, 55 genes had an overall prevalence of more than 50% and a mean abundance over 2,500,000 FPKM across the metagenomes. Only six of these highly prevalent genes confer resistance to two relevant drug classes: tetracycline and beta-lactam. **b.** Individual mouse relative abundances by resistance mechanism. Genes related to target alteration and protection were consistently more abundant in all mouse metagenomes.

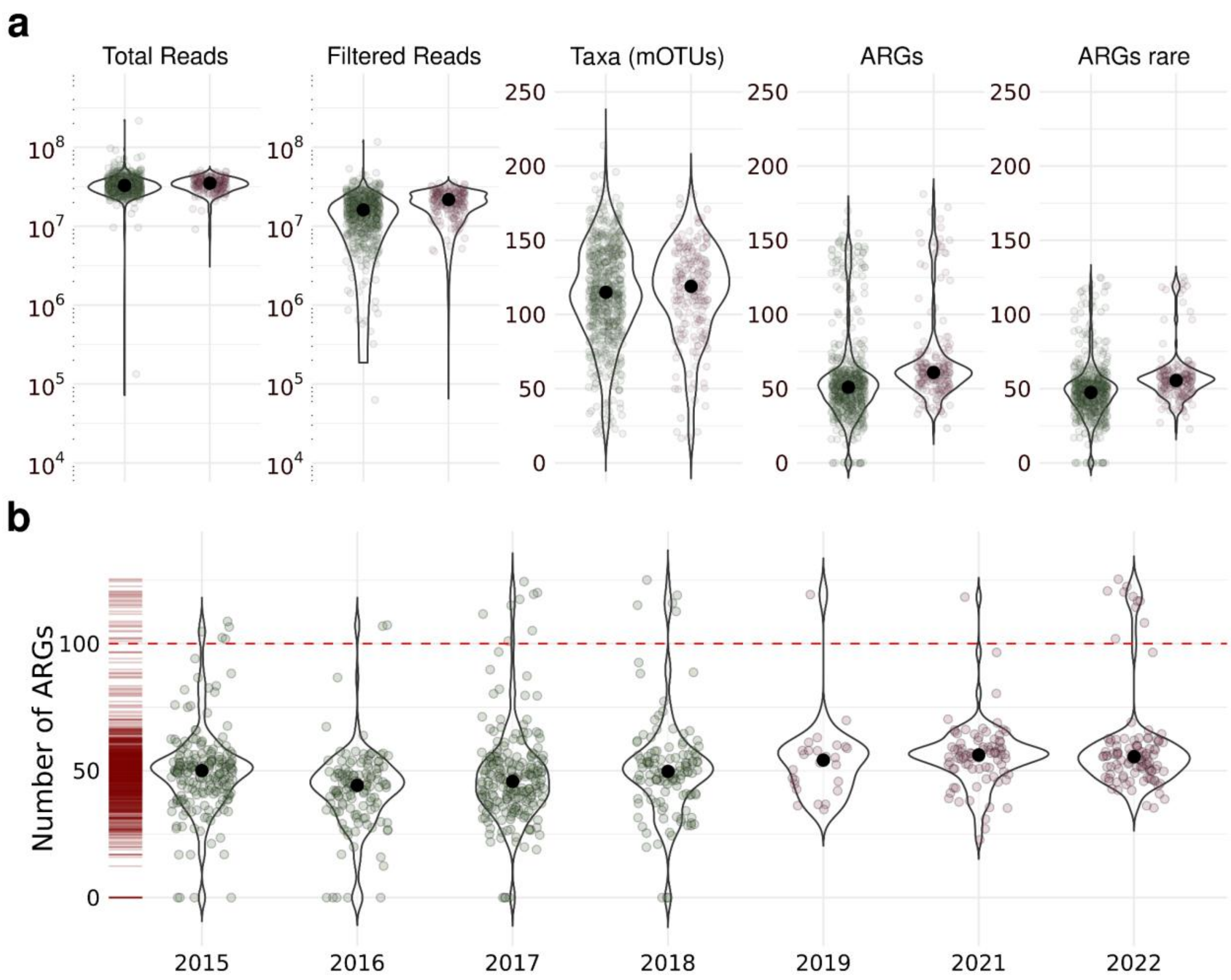

Supplementary Fig. 2. Effect of sampling campaigns in sequencing output, taxonomy and ARGs. a. Significant differences on total reads, filtered reads after preprocessing and number of ARGs without and with rarefaction were observed between sampling campaigns (green = 2015-2018, pink = 2019 to 2022), although the effect size of the difference in sequencing parameters was lower compared to the effect in ARG differences. **b.** The number of rarefied ARGs detected in 2016 was significantly lower than the number detected in 2015 and 2018 within the same sampling campaign (Two-sided Wilcoxon rank-sum test, *p*-value Bonferroni adjusted <0.001, Supplementary Table 1). There was no difference in ARGs detected between years at the end of the study period. Most mice (n = 843) had an ARG richness of around 50 genes, while a smaller group (n = 32) had a higher number of genes detected. The dashed red line defines a threshold of 100 ARGs detected. Each coloured point represents a single mouse metagenome. Black points indicate the median.

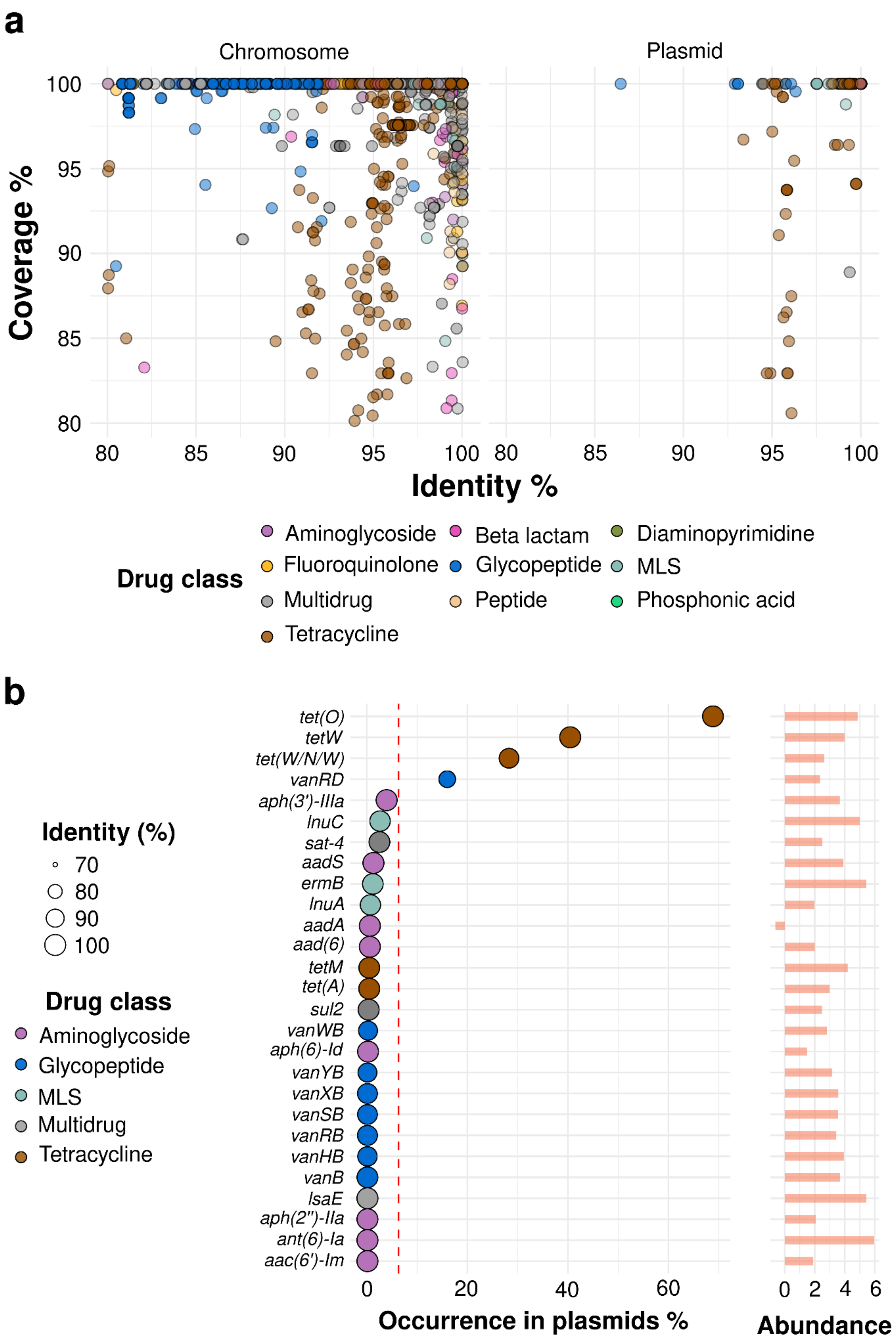

Supplementary Fig. 3. Genetic context of ARGs in contigs with more than 1000b. a. Out of 5874 contigs containing genes with >=80% identity and >=80% coverage, 96% of these

contigs correspond to genes in bacterial main chromosomes (N= 5687) and 235 contigs correspond to genes localised in plasmids. **b.** Plasmids carry 27 different ARGs, three *tet* genes co-localised at plasmids were detected in more than 20% of samples, the rest of genes were detected in plasmids for less than 10% of the samples. The mean abundance was 15 FPKM across the plasmids. The size of the points represent the percentage of identity to the reference ARG- The abundance is expressed as log2 FPKM.

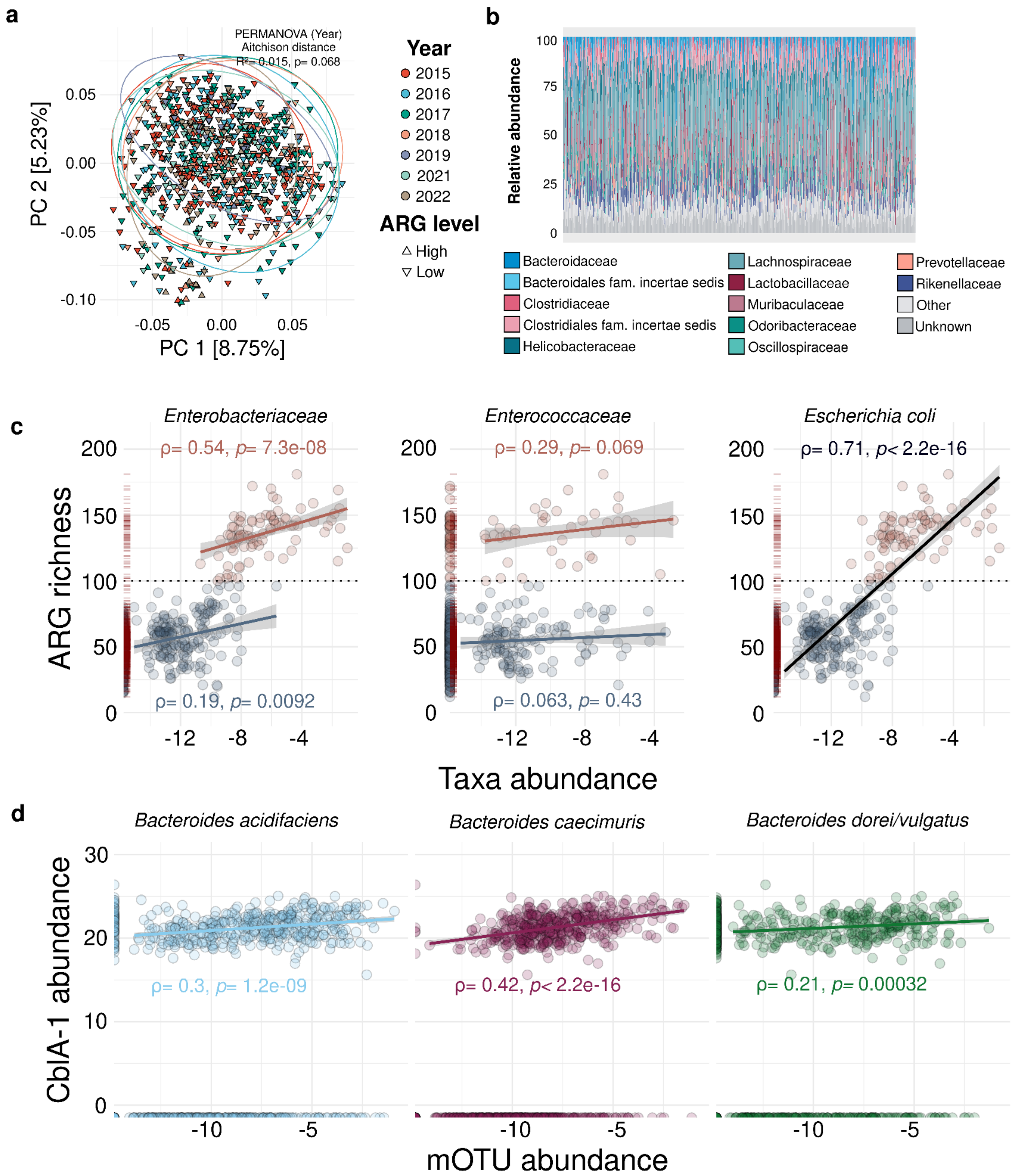

Supplementary Fig. 4. General overview of the colon microbiomes from house mice and their relationship to ARG levels. a. Principal component analysis (PCA) showing dissimilarity in microbial composition between years of sampling. Each point represents the colon microbiome of a sample. Distances between points reflect biological gene composition

dissimilarity based on Aitchison distances. Points and ellipses are coloured by year of sampling. Shape represents whether an individual metagenome has higher or lower than 100 ARGs predicted. **b.** Individual mouse relative abundances of operational taxonomic (mOTUs) units agglomerated by bacterial families. mOTUs from the Lachnospiraceae family were consistently more abundant and prevalent in all mouse metagenomes (Mean relative abundance= 25.8% [95%CI: 24.8-26.8], Prevalence= 99.8%). **c.** Spearman's rank correlation between microorganisms commonly associated with resistance and ARG richness per mouse. Commensal gut families (*Enterobacteriaceae*, *Enterococcaceae*) explain differences in ARG levels in mice. Higher abundance of such families is related to higher levels of resistance. *Escherichia coli* has a strong correlation to the total ARG richness. **d.** The abundance of the *cblA-1* gene coding for a Class A beta lactamase is related to *Bacteroides* sp. Within the house mouse microbiome. *B. caecimuris* abundance has a stronger correlation compared to the other two *Bacteroides* species with prevalence above 50%. Taxa abundance is expressed as the log2 of the relative abundance, and for the gene *cblA-1* the abundance is expressed as log2 FPKM.

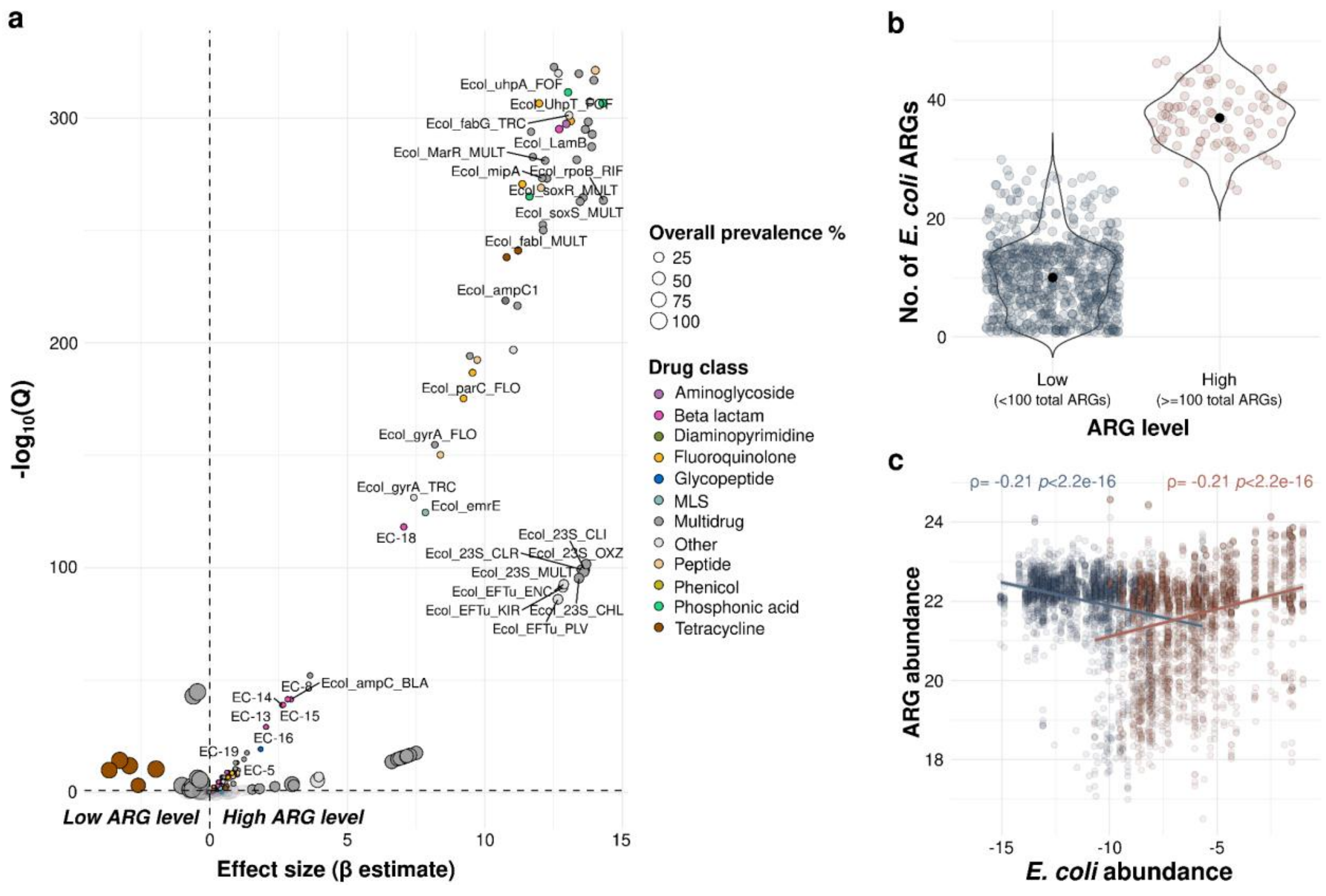

Supplementary Fig. 5. Mice with high ARG level (>= 100 ARGs) are characterised by *Escherichia coli* related genes. a. 48 genes related to *E. coli* were differentially abundant and **b.** significantly higher in occurrence in mice with higher ARG richness (Two-sided Wilcoxon rank-sum test, r=0.52, *p*-value= 2.39e-51). The volcano plot shows the genes' different abundance between the two subsets of house mice metagenomes. The size of the points indicates its prevalence across metagenomes and color the drug class. Violin plot shows that mice with higher ARG richness carry significantly more resistance conferring SNPs in housekeeping genes. **c.** The abundance of those *E. coli* related genes was positively correlated to *E. coli* abundance in the house mice metagenomes. Spearman's correlation

between *E. coli* and ARGs. The abundance for both *E. coli* and ARGs is expressed as log2 relative abundance or FPKM, respectively.

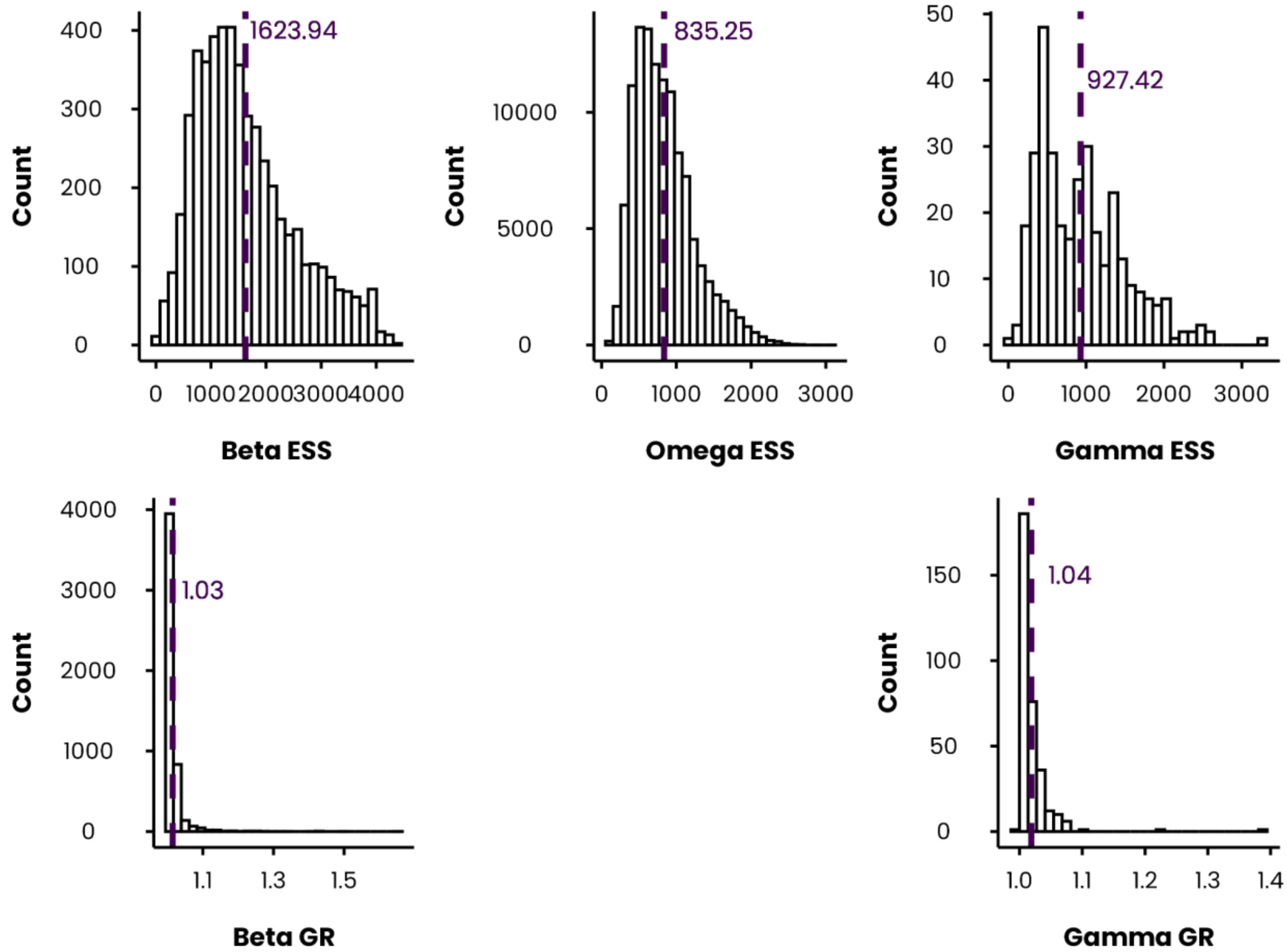

Supplementary Fig. 6. Convergence diagnostics for the ARG presence-absence joint species distribution model (jSDM). The top row shows the distribution of effective sample sizes (ESS) for monitored parameters, with the dashed purple line indicating the average ESS. The bottom row shows the distribution of Gelman–Rubin (GR) statistics, with the dashed purple line marking the average GR value. Model convergence: The average effective sample sizes for all parameters are high (>800), indicating that the model estimates robust parameters and exhibits low autocorrelation and good mixing. Gelman-Rubin diagnostic values are acceptable for betas (1.03) and gammas (1.04), which means chains converge well. It could not be compiled for omegas for structural reasons (huge matrices for each sample). Model fit metrics: Pseudo-R^2 (Tjur R^2) = 0.13 , AUC = 0.91, R^2 sp = 0.17, R^2 site = 0.72.

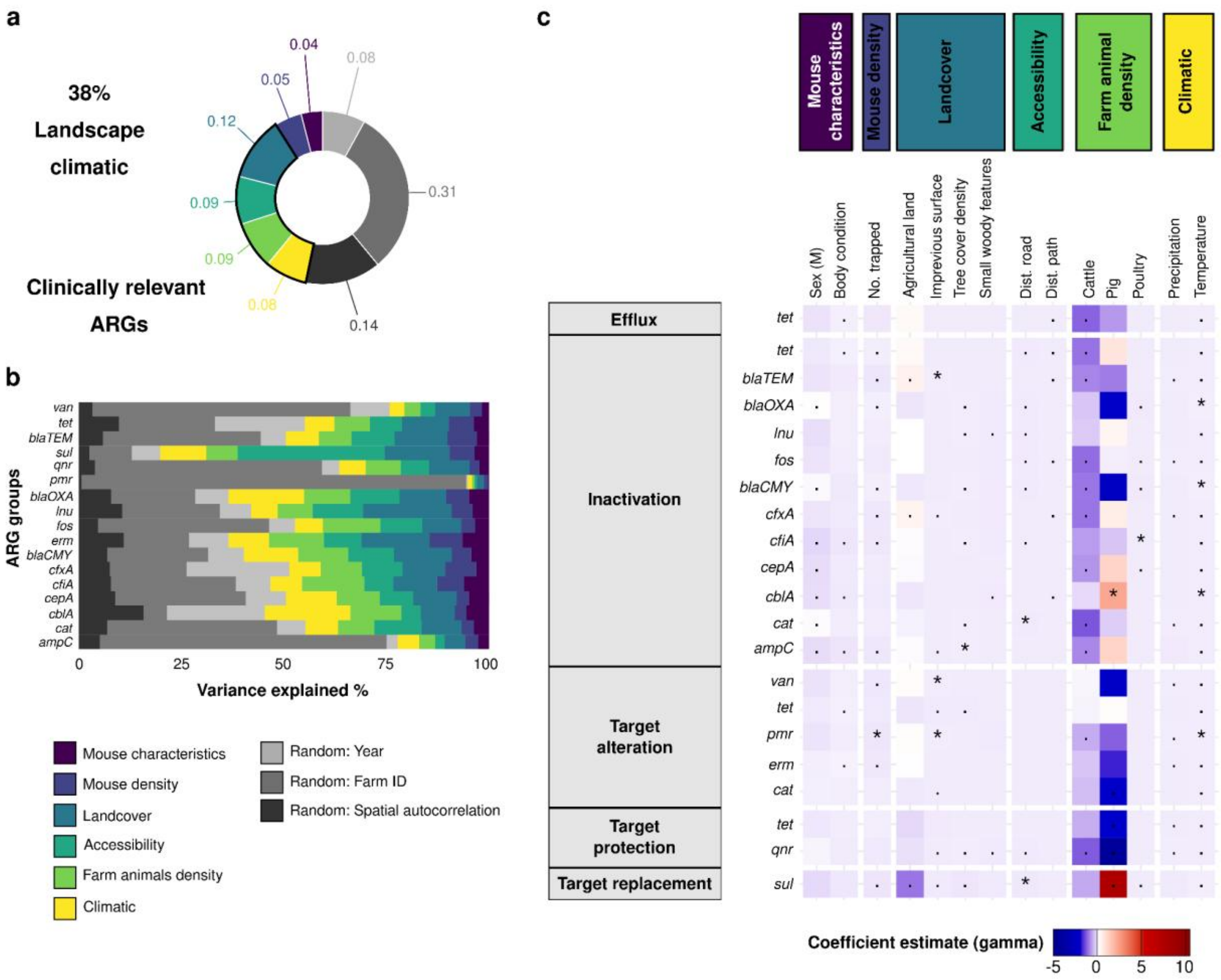

Supplementary Fig. 7. Variance partitioning showing the relative contribution of mouse characteristics, environmental factors, and random effects, on the explained variation in clinically relevant ARG occurrence in mouse gut microbiomes. a. Landcover accounts for the higher proportion of variance on these genes **b.** Variance partitioning in occurrence averaged for each ARG group. For *sul* genes landscape characteristics are particularly relevant and account for more than 70% of occurrence variance. **c.** The heatmap shows the coefficient estimate (gamma) for each ARG group and a given explanatory variable of the jSDM. Cattle and pig density are explanatory variables with stronger associations compared to other variables in the model. *sul* genes with target replacement mechanism and the *cblA-1* beta-lactamase are strongly and positively associated with pig density ($\gamma > 5$, support: 0.75). *tet* genes are distinctively associated with pig density, while those related to antibiotic inactivation have a positive association, those involved in efflux and target protection mechanisms are negatively associated, and especially the latter with a strong effect ($\gamma < 0$, support: 0.75). The higher the coefficient estimate, the higher the association of the variable with the ARG group. Symbol in tile indicate the posterior probability of the estimate in the model: $*$ > 0.95 and $\cdot$ > 0.75.

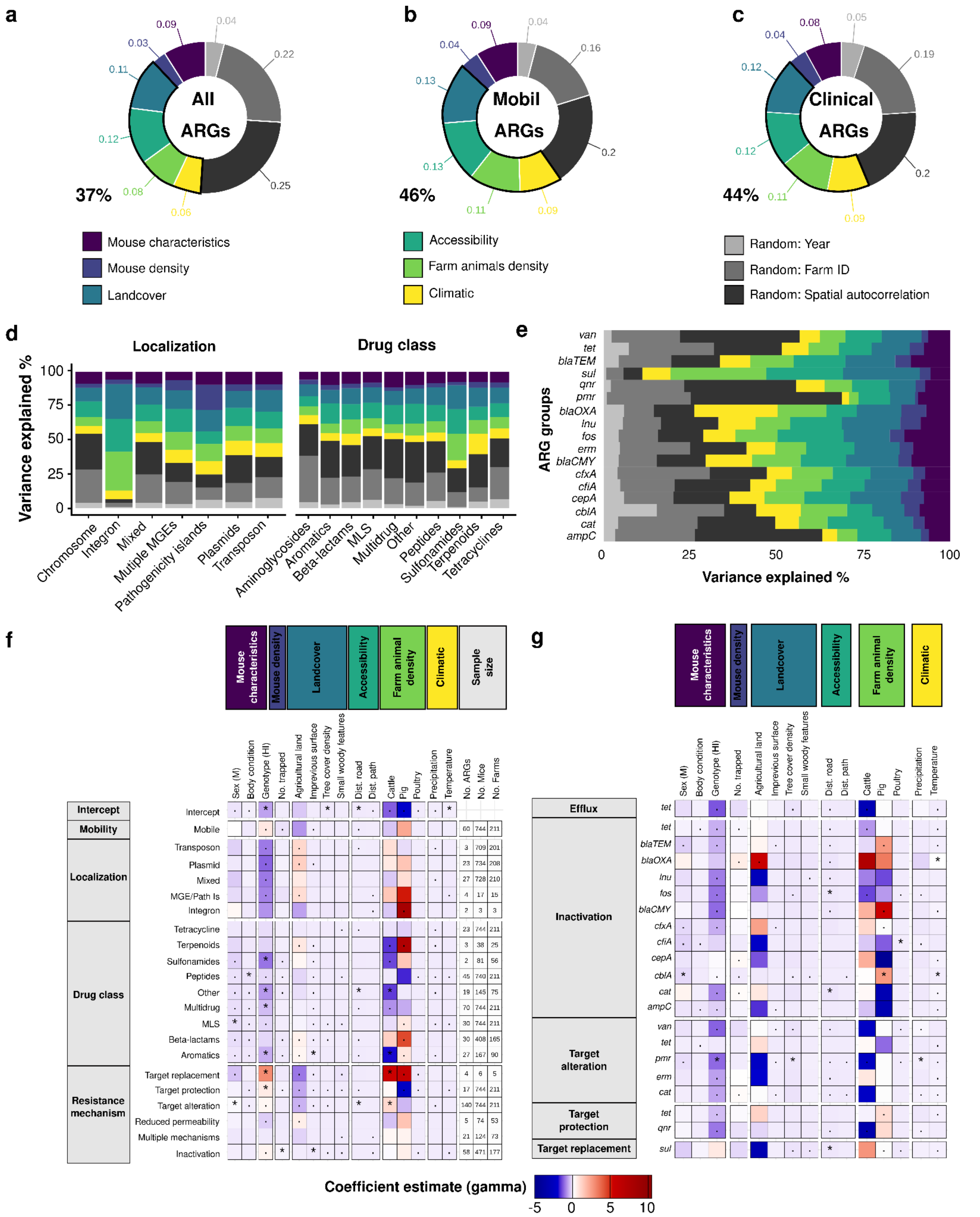

Supplementary Fig. 8. Variance partitioning showing the relative contribution of mouse characteristics, environmental factors, and random effects, on the explained variation **a.** in all ARGs detected, **b.** potentially mobile ARGs and **c.** clinically relevant ARG occurrence in mouse gut microbiomes in a model that include host genotype as an additional host characteristic. **d.** The variance partitioning averaged for each ARG's traits shows the occurrence of genes with potential localization in integrons, genes conferring resistance to sulfonamides and conferring resistance to sulfonamides are more influenced by environmental factors. **e.** Variance partitioning in occurrence averaged for each clinically relevant ARG group. For *sul* genes landscape characteristics are particularly relevant and account for more

than 70% of occurrence variance. The heatmaps show the coefficient estimate (gamma) for **f.** the ARG traits or for **g.** each clinically relevant ARG group and a given explanatory variable of the jSDM. The higher the coefficient estimate, the higher the association of the variable with the ARG group. Symbol in tile indicate the posterior probability of the estimate in the model: ∗ > 0.95 and · > 0.75.

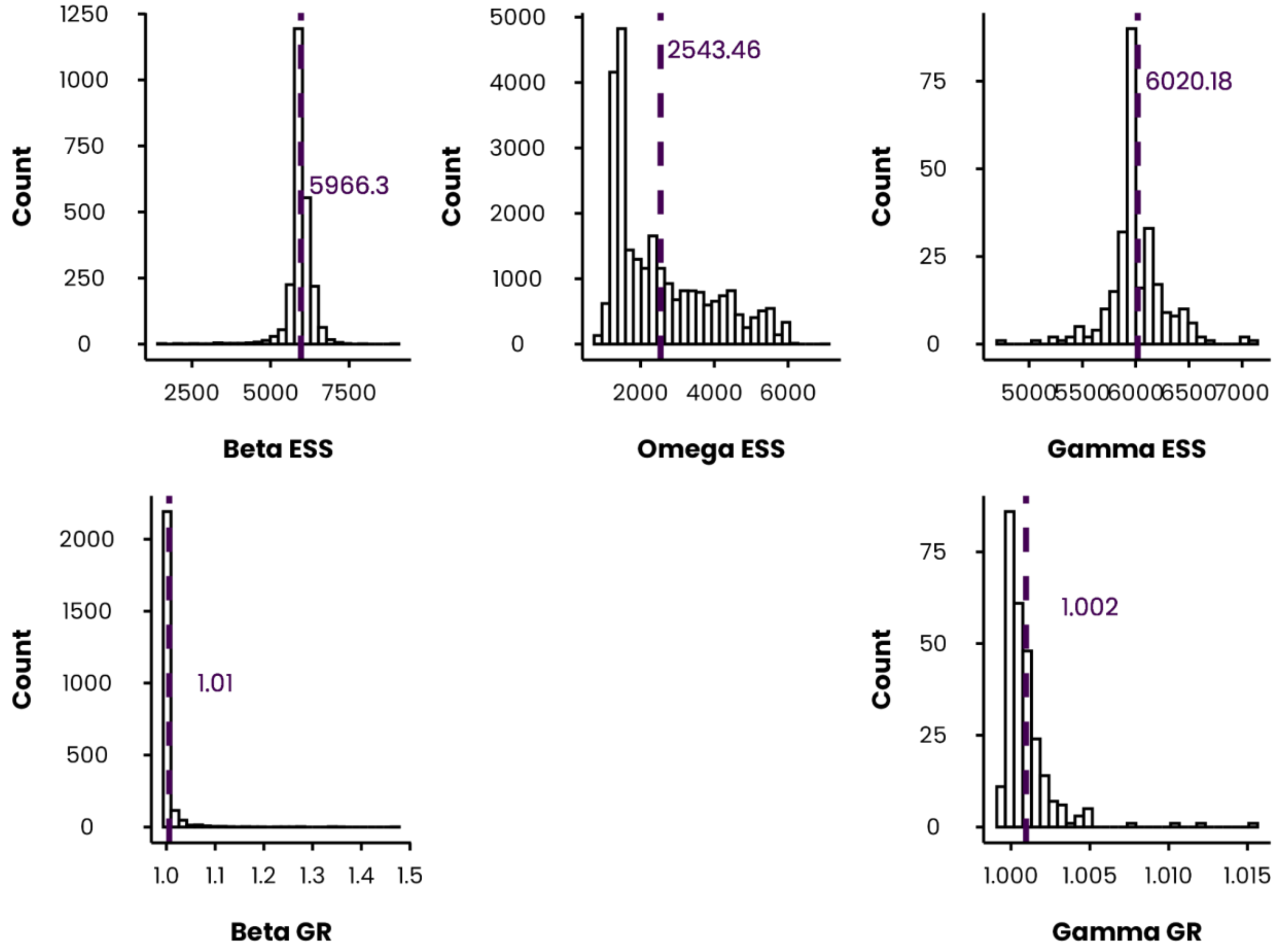

Supplementary Fig. 9. Convergence diagnostics for the jSDM ARG abundance model. The top row shows the distribution of effective sample sizes (ESS) for monitored parameters, with the dashed purple line indicating the average ESS. The bottom row shows the distribution of Gelman–Rubin (GR) statistics, with the dashed purple line marking the average GR value. Model convergence: The average effective sample sizes for all parameters are high (>2500), indicating that the model estimates robust parameters and exhibits low autocorrelation and good mixing. Gelman-Rubin diagnostic values are acceptable for betas (1.01) and gammas (1.002), which means chains converge well. Model fit metrics: Pseudo-R^2 (Tjur R^2) = 0.22 , AUC = 0.796, R^2 sp = 0.22, R^2 site = 0.81

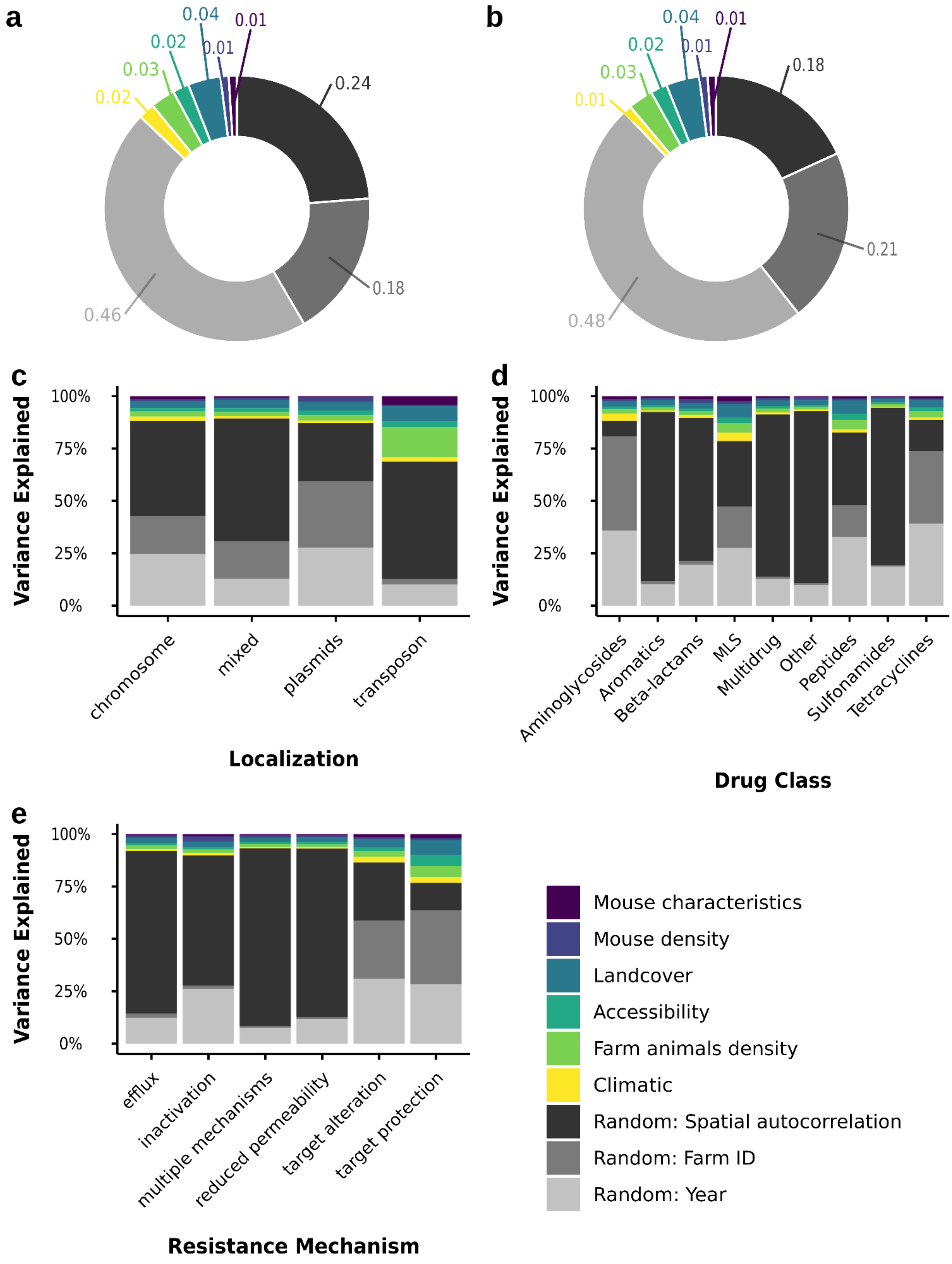

Supplementary Fig. 10. Variance partitioning showing the relative contribution of mouse characteristics, environmental factors, and random effects, on the explained variation in ARGs abundance in mouse gut microbiomes a. for all genes and **b.** only for mobile genes. Variance partitioning in abundance averaged for each ARG trait (**c.** Localization, **d.** Drug class and **e.** Resistance mechanism).

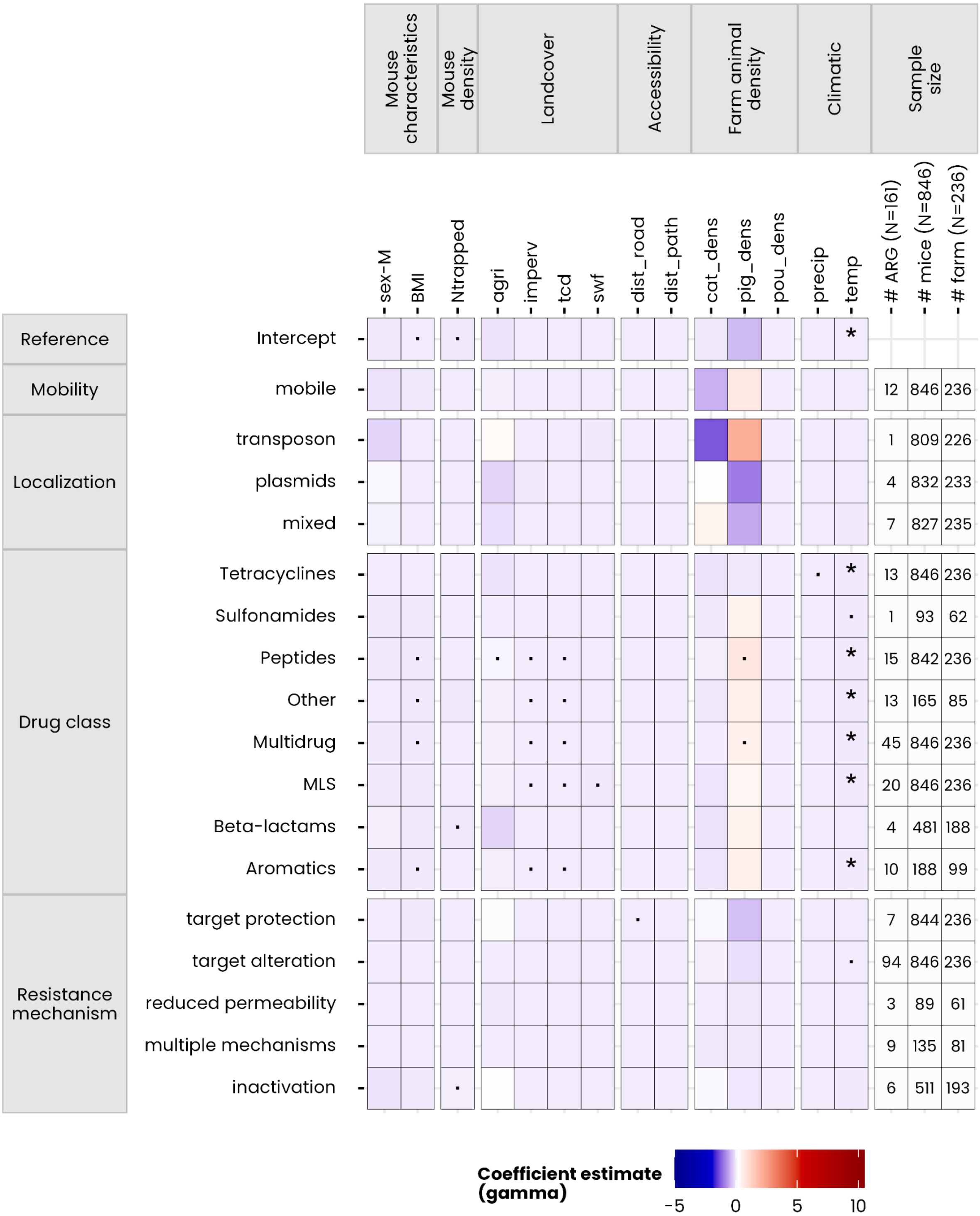

Supplementary Fig. 11. Effect of mouse-related, environmental, farm animal density and climatic variables on ARG abundance agglomerated according to their traits. The intercept reference level for traits corresponds to ARGs that are non-mobile, located on chromosomes, giving resistance towards the aminoglycoside drug class, and having an efflux resistance mechanism. Symbol in tile indicate the posterior probability of the estimate in the model: $*$ > 0.95 and $\cdot$ > 0.75.

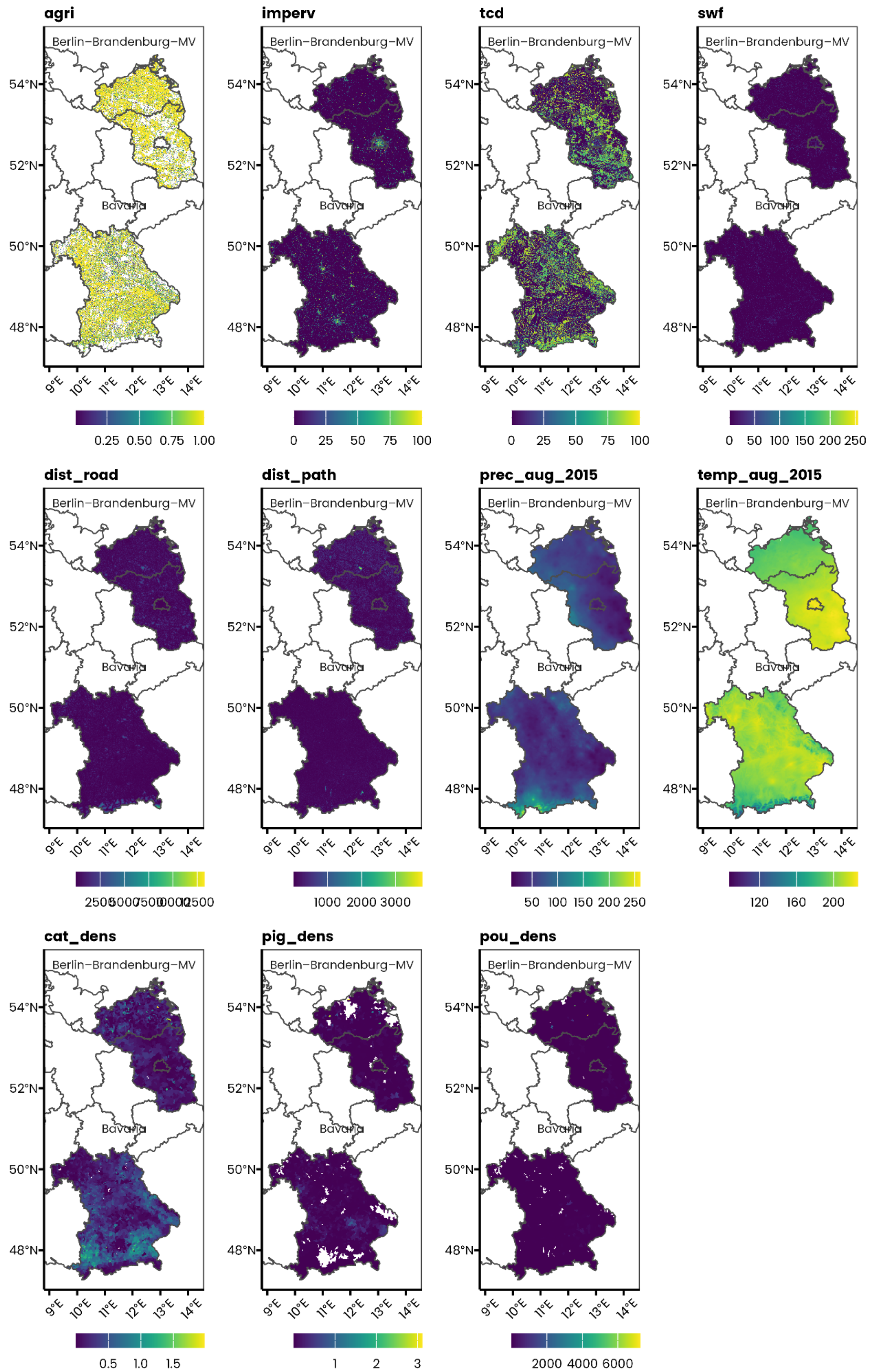

Supplementary Fig. 12. Maps of environmental layers in the sampled regions (Berlin-Brandenburg-Mecklenburg-Vorpommern and Bavaria). For precipitation and temperature, we only displayed the values recorded during the year 2015. Overview maps from Germany and the federal states were created using a public domain map dataset including vector

country and other administrative boundaries from Natural Earth. Made with Natural Earth. Free vector and raster map data @ naturalearthdata.com.

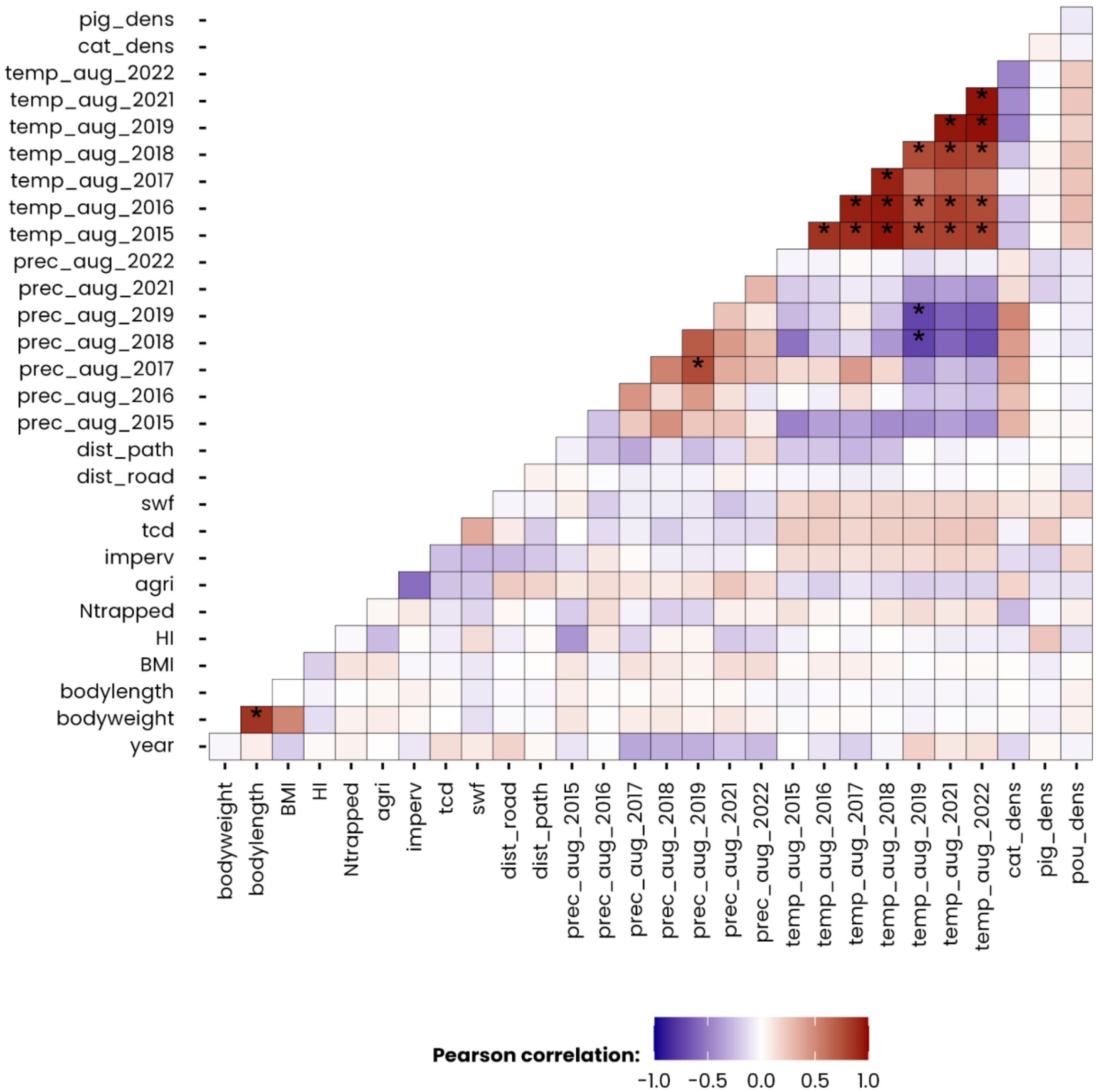

Supplementary Fig. 13. Pearson correlation matrix showing the pairwise linear relationships between the explanatory variables. Colours indicate the strength and direction of the correlation, with blue representing negative correlations and red representing positive correlations. Symbol $*$ in tile indicates correlation values |r| > 0.7.

Supplementary Tables

Supplementary Table 1. Effect of sampling campaigns in sequencing output, taxonomy and ARGs

Measurement	N Group 1	N Group 2	P-value	Significance	Effect size
Total reads	661	214	4.59E-03	**	0.096
Filtered reads	661	214	4.18E-17	****	0.284
Taxa (mOTUs)	645	214	0.223	NS	0.030
ARGs	661	214	2.23E-19	****	0.304
ARGs rarefied	661	214	2.26E-19	****	0.304

Two-sided Wilcoxon rank-sum test. Exact P-values for each independent comparison for each measurement.

Supplementary Table 2. Differences in ARG richness between house mice and livestock manure

Host 1	Host 2	P-value adj.	Significance	Effect size	Contrast
House mice (n=859)	Cattle (n=29)	4.73E-19	****	0.31	306±16.4
	Chicken (n=238)	1.29E-121	****	0.71	416±6.4
	Pig (n=269)	6.10E-132	****	0.73	173±6.1
	Turkey (n=17)	1.56E-12	****	0.24	497±21.2

Two-sided Wilcoxon rank-sum test. P adjusted after Bonferroni correction.

Supplementary Table 3. Classification of antimicrobial resistance genes (ARGs) into drug classes and broader drug class groups for the association analysis in the joint species distribution models (jSDMs). To address the sparse representation of certain drug classes, ARGs were grouped into broader drug class groups based on chemical structure and mechanism of action. This table details the reclassification of individual drug classes to their corresponding broader groups, along with the number of ARGs identified per class and per group.

Drug class group	Drug class	Number of ARGs per class	Number of ARGs per group
Aminoglycosides	aminoglycoside	59	59
Aromatics	fluoroquinolone	23	29
	phenicol	3	
	rifamycin	3	
Beta-lactams	beta-lactam	8	39
	carbapenem	4	
	cephalosporin	27	
MLS	elfamycin	7	32
	lincosamide	6	
	macrolide	18	
	streptogramin	1	
Multidrug	multidrug	81	81
Other	aminocoumarin	4	22
	diaminopyrimidine	4	
	mupirocin	1	
	nitroimidazole	1	
	nucleoside	1	
	phosphonic acid	10	
	zoliflodacin	1	
Peptides	glycopeptide	25	48
	peptide	23	

Sulfonamides	sulfonamide	3	3
Terpenoids	fusidane	1	3
	pleuromutilin	2	
Tetracyclines	tetracycline	24	24

Supplementary Table 4. Clinically relevant groups of antimicrobial resistance genes (ARGs).

Gene group	Description	Resistance mechanism	Drug class
cblA	CblA beta-lactamase	antibiotic inactivation	cephalosporin
cepA	CepA beta-lactamase	antibiotic inactivation	cephalosporin
cfxA	CfxA beta-lactamase	antibiotic inactivation	cephamycin
blaCTX-M	CTX-M beta-lactamase	antibiotic inactivation	cephalosporin
blaKPC	KPC beta-lactamase	antibiotic inactivation	monobactam, carbapenem, cephalosporin, penam
blaSHV	SHV beta-lactamase	antibiotic inactivation	carbapenem, cephalosporin, penam
blaTEM	TEM beta-lactamase	antibiotic inactivation	penam, monobactam, cephalosporin, penem
cfiA	CfiA beta-lactamase	antibiotic inactivation	carbapenem
blaIMP	IMP beta-lactamase	antibiotic inactivation	carbapenem, cephalosporin, cephamycin, penam
blaNDM	NDM beta-lactamase	antibiotic inactivation	carbapenem,cephalosporin,cephamycin,penam
blaVIM	VIM beta-lactamase	antibiotic inactivation	carbapenem, cephalosporin, cephamycin, penam
blaACT	ACT beta-lactamase	antibiotic inactivation	carbapenem, cephalosporin,

			cephamycin, penam
ampC	ampC-type beta-lactamase	antibiotic inactivation	cephalosporin, penam
blaCMY	CMY beta-lactamase	antibiotic inactivation	cephamycin
blaOXA	OXA beta-lactamase	antibiotic inactivation	carbapenem, cephalosporin, penam
aac(3)	AAC(3)	antibiotic inactivation	aminoglycoside
aac(6')	AAC(6')	antibiotic inactivation	aminoglycoside
ant(3")	ANT(3")	antibiotic inactivation	aminoglycoside
ant(4')	ANT(4')	antibiotic inactivation	aminoglycoside
ant(6)	ANT(6)	antibiotic inactivation	aminoglycoside
aph(2")	APH(2")	antibiotic inactivation	aminoglycoside
aph(3')	APH(3')	antibiotic inactivation	aminoglycoside
aph(3")	APH(3")	antibiotic inactivation	aminoglycoside
aph(6)	APH(6)	antibiotic inactivation	aminoglycoside
vanA	vanA/vanB	antibiotic target alteration	glycopeptide
qnr	Quinolone resistance protein	antibiotic target protection	fluoroquinolone
mcr	Phosphoethanolamine transferase	antibiotic target alteration	peptide
pmr	Phosphoethanolamine transferase	antibiotic target alteration	peptide
fos	Fosfomycin thiol transferase	antibiotic inactivation	phosphonic acid
cat	Chloramphenicol acetyltransferase	antibiotic inactivation	phenicol
tet	tetracycline-resistant ribosomal protection protein	antibiotic target protection	tetracycline
tet	tetracycline inactivation enzyme	antibiotic inactivation	tetracycline

cfr	Cfr 23S ribosomal RNA methyltransferase	antibiotic target alteration	oxazolidinone, streptogramin, phenicol, lincosamide
erm	Erm 23S ribosomal RNA methyltransferase	antibiotic target alteration	macrolide, lincosamide, streptogramin
lnu	Lincosamide nucleotidyltransferase	antibiotic inactivation	lincosamide
ere	Macrolide esterase	antibiotic inactivation	macrolide
mph	Macrolide phosphotransferase	antibiotic inactivation	macrolide
arr	rifampin ADP-ribosyltransferase	antibiotic inactivation	rifamycin
sul	Sulfonamide resistant	antibiotic target replacement	sulfonamide

Supplementary Table 5. Environmental layers used for modelling ARG occurrence in the mouse gut microbiome. All spatial layers contain continuous data. Year refers to the year of origin of the data. For distance to roads and paths, the references provided refer to the original layers of respective streets and paths used to calculate the distances. Corine Land Cover layer was used to calculate the proportion of agricultural fields (*agri*).

Variable	Environmental layer	Units	Year	Original resolution
agri	CORINE land cover[1]	proportion of agricultural land (land use class 2)	2018	vector data
imperv	Imperviousness[1]	proportion of impervious surface per raster cell	2018	10m raster
tcd	Tree cover density[1]	proportion of area occupied by trees per raster cell	2018	10m raster
swf	Small woody features[1]	proportion of area occupied by linear structures such as hedgerows, as well as patches of woody features per raster cell	2018	100m raster
dist_roads, dist_paths	Distance to roads, distance to paths [2]	streets and paths linear, rasterised and transformed into distances in meters	2018	vector data
catdens, pigdens, poudens	Livestock[3]	Livestock units of cattle, pigs and poultry, transformed into density per hectare	2020	vector data

prec	Precipitation[4]	Monthly total precipitation (l/m2 or mm)	2015-2022	1km raster
temp	Temperature[4]	Monthly average air temperature (1/10 °C)	2015-2022	1km raster

[1] https://land.copernicus.eu/en/products/ (Downloaded 06.02.2024)
[2] obtained from GeoBasis-DE / BKG (2022): https://gdz.bkg.bund.de/index.php/ (Downloaded 07.02.2024)
[3] obtained from the Thünen Atlas: Landwirtschaftliche Nutzung Winterweizen / Landwirtschaftliche genutzte Fläche 2020, https://atlas.thuenen.de/webspace/agraratlas/agraratlas/index.html?LP=2 (Downloaded 18.09.2023)
[4] obtained from the Deutscher Wetterdients: https://opendata.dwd.de (Downloaded 05.11.2024)

Supplementary Table 6. Livestock manure sequencing runs used for comparative analysis. Run accession numbers and additional metadata from the livestock manure metagenomes included in the comparative analysis.

Bioproject	Run accession	Collection date	Location	Host
PRJEB62878	ERR11530773	2020	Ghana	chicken
PRJEB62878	ERR11530818	2020	Ghana	chicken
PRJEB62878	ERR11530933	2020	Ghana	chicken
PRJEB62878	ERR11531012	2020	Ghana	chicken
PRJEB62878	ERR11531680	2020	Ghana	chicken
PRJEB62878	ERR11531835	2020	Ghana	chicken
PRJEB62878	ERR11531875	2020	Ghana	chicken
PRJEB62878	ERR11532577	2020	Ghana	chicken
PRJEB62878	ERR11532797	2020	Ghana	chicken
PRJEB62878	ERR11532806	2020	Ghana	chicken
PRJEB62878	ERR11532862	2020	Ghana	chicken

PRJEB62878	ERR11532867	2020	Ghana	chicken
PRJEB62878	ERR11532915	2020	Ghana	chicken
PRJEB62878	ERR11532927	2020	Ghana	chicken
PRJEB62878	ERR11532933	2020	Ghana	chicken
PRJEB62878	ERR11532952	2020	Ghana	chicken
PRJEB62878	ERR11532957	2020	Ghana	chicken
PRJEB62878	ERR11532960	2020	Ghana	chicken
PRJEB62878	ERR11533227	2020	Ghana	chicken
PRJEB62878	ERR11533452	2020	Ghana	chicken
PRJEB62878	ERR11533467	2020	Ghana	chicken
PRJEB62878	ERR11533471	2020	Ghana	chicken
PRJEB62878	ERR11533532	2020	Ghana	chicken
PRJEB62878	ERR11533554	2020	Ghana	chicken
PRJEB62878	ERR11533599	2020	Ghana	chicken
PRJEB62878	ERR11533617	2020	Ghana	chicken
PRJEB62878	ERR11533667	2020	Ghana	chicken
PRJEB62878	ERR11533709	2020	Ghana	chicken
PRJEB62878	ERR11533759	2020	Ghana	chicken
PRJEB62878	ERR11533792	2020	Ghana	chicken
PRJEB62878	ERR11533822	2020	Ghana	chicken

PRJEB62878	ERR11533855	2020	Ghana	chicken
PRJEB62878	ERR11533894	2020	Ghana	chicken
PRJEB62878	ERR11533918	2020	Ghana	chicken
PRJEB62878	ERR11533953	2020	Ghana	chicken
PRJEB62878	ERR11533991	2020	Ghana	chicken
PRJEB62878	ERR11534017	2020	Ghana	chicken
PRJEB62878	ERR11534054	2020	Ghana	chicken
PRJEB62878	ERR11534088	2020	Ghana	chicken
PRJEB62878	ERR11534153	2020	Ghana	chicken
PRJEB62878	ERR11534223	2020	Ghana	chicken
PRJEB62878	ERR11534255	2020	Ghana	chicken
PRJEB62878	ERR11534445	2020	Ghana	chicken
PRJEB62878	ERR11534478	2020	Ghana	chicken
PRJEB62878	ERR11534529	2020	Ghana	chicken
PRJEB62878	ERR11534576	2020	Ghana	chicken
PRJEB62878	ERR11534607	2020	Ghana	chicken
PRJEB62878	ERR11534662	2020	Ghana	chicken
PRJEB62878	ERR11534711	2020	Ghana	chicken
PRJEB62878	ERR11534754	2020	Ghana	chicken
PRJEB62878	ERR11534771	2020	Ghana	chicken

PRJEB62878	ERR11534794	2020	Ghana	chicken
PRJEB62878	ERR11534847	2020	Ghana	chicken
PRJEB62878	ERR11534892	2020	Ghana	chicken
PRJEB62878	ERR11534911	2020	Ghana	chicken
PRJEB62878	ERR11534955	2020	Ghana	chicken
PRJEB62878	ERR11534995	2020	Ghana	chicken
PRJEB62878	ERR11535044	2020	Ghana	pig
PRJEB62878	ERR11535059	2020	Ghana	pig
PRJEB62878	ERR11535072	2020	Ghana	pig
PRJEB62878	ERR11535074	2020	Ghana	pig
PRJEB62878	ERR11535077	2020	Ghana	pig
PRJEB62878	ERR11535079	2020	Ghana	pig
PRJEB62878	ERR11535098	2020	Ghana	pig
PRJEB62878	ERR11535100	2020	Ghana	pig
PRJEB62878	ERR11535102	2020	Ghana	pig
PRJEB62878	ERR11535124	2020	Ghana	pig
PRJEB62878	ERR11535192	2020	Ghana	pig
PRJEB62878	ERR11535269	2020	Ghana	pig
PRJEB62878	ERR11535320	2020	Ghana	pig
PRJEB62878	ERR11535340	2020	Ghana	pig

PRJEB62878	ERR11535360	2020	Ghana	pig
PRJEB62878	ERR11535400	2020	Ghana	pig
PRJEB62878	ERR11535410	2020	Ghana	pig
PRJEB62878	ERR11535641	2020	Ghana	pig
PRJEB62878	ERR12015094	2020	Ghana	chicken
PRJEB62878	ERR12020496	2020	Ghana	chicken
PRJEB62878	ERR12020517	2020	Ghana	chicken
PRJEB62878	ERR12020518	2020	Ghana	pig
PRJEB62878	ERR12020519	2020	Ghana	pig
PRJEB62878	ERR12020520	2020	Ghana	pig
PRJEB62878	ERR12020521	2020	Ghana	pig
PRJEB62878	ERR12020522	2020	Ghana	pig
PRJEB62878	ERR12020523	2020	Ghana	pig
PRJEB62878	ERR12020524	2020	Ghana	pig
PRJEB62878	ERR12020525	2020	Ghana	pig
PRJEB62878	ERR12020526	2020	Ghana	pig
PRJEB62878	ERR12020527	2020	Ghana	pig
PRJEB62878	ERR12020528	2020	Ghana	pig
PRJEB62878	ERR12020694	2020	Ghana	pig
PRJEB22062	ERR2241624	2015	Netherlands	pig

PRJEB22062	ERR2241625	2015	Netherlands	pig
PRJEB22062	ERR2241626	2015	Netherlands	pig
PRJEB22062	ERR2241627	2015	Netherlands	pig
PRJEB22062	ERR2241628	2015	Netherlands	chicken
PRJEB22062	ERR2241629	2015	Netherlands	pig
PRJEB22062	ERR2241630	2015	Netherlands	pig
PRJEB22062	ERR2241631	2015	Netherlands	chicken
PRJEB22062	ERR2241632	2015	Netherlands	chicken
PRJEB22062	ERR2241633	2015	Netherlands	pig
PRJEB22062	ERR2241634	2015	Netherlands	chicken
PRJEB22062	ERR2241635	2015	Netherlands	chicken
PRJEB22062	ERR2241636	2015	Netherlands	pig
PRJEB22062	ERR2241637	2015	Netherlands	chicken
PRJEB22062	ERR2241638	2015	Netherlands	pig
PRJEB22062	ERR2241639	2015	Netherlands	chicken
PRJEB22062	ERR2241640	2015	Netherlands	pig
PRJEB22062	ERR2241641	2015	Netherlands	chicken
PRJEB22062	ERR2241642	2015	Netherlands	pig
PRJEB22062	ERR2241643	2015	Netherlands	chicken
PRJEB22062	ERR2241644	2015	Netherlands	pig

PRJEB22062	ERR2241645	2015	Netherlands	chicken
PRJEB22062	ERR2241646	2015	Netherlands	pig
PRJEB22062	ERR2241647	2015	Netherlands	chicken
PRJEB22062	ERR2241648	2015	Netherlands	pig
PRJEB22062	ERR2241649	2015	Netherlands	chicken
PRJEB22062	ERR2241650	2015	Netherlands	pig
PRJEB22062	ERR2241651	2015	Netherlands	chicken
PRJEB22062	ERR2241652	2015	Netherlands	pig
PRJEB22062	ERR2241653	2015	Netherlands	chicken
PRJEB22062	ERR2241654	2015	Netherlands	pig
PRJEB22062	ERR2241655	2015	Netherlands	chicken
PRJEB22062	ERR2241656	2015	Netherlands	pig
PRJEB22062	ERR2241657	2015	Netherlands	chicken
PRJEB22062	ERR2241658	2015	Netherlands	pig
PRJEB22062	ERR2241659	2015	Netherlands	chicken
PRJEB22062	ERR2241660	2015	Netherlands	pig
PRJEB22062	ERR2241661	2015	Netherlands	pig
PRJEB22062	ERR2241662	2015	Netherlands	chicken
PRJEB22062	ERR2241663	2015	Netherlands	pig
PRJEB22062	ERR2241664	2015	Netherlands	chicken

PRJEB22062	ERR2241665	2015	Netherlands	chicken
PRJEB22062	ERR2241666	2015	Germany	pig
PRJEB22062	ERR2241667	2015	Germany	pig
PRJEB22062	ERR2241668	2015	Germany	pig
PRJEB22062	ERR2241669	2015	Germany	pig
PRJEB22062	ERR2241670	2015	Germany	pig
PRJEB22062	ERR2241671	2015	Germany	pig
PRJEB22062	ERR2241672	2015	Germany	pig
PRJEB22062	ERR2241673	2015	Germany	pig
PRJEB22062	ERR2241674	2015	Germany	pig
PRJEB22062	ERR2241675	2015	Germany	pig
PRJEB22062	ERR2241676	2015	Germany	pig
PRJEB22062	ERR2241677	2015	Germany	pig
PRJEB22062	ERR2241678	2015	Germany	pig
PRJEB22062	ERR2241679	2015	Germany	pig
PRJEB22062	ERR2241680	2015	Germany	pig
PRJEB22062	ERR2241681	2015	Germany	pig
PRJEB22062	ERR2241682	2015	Germany	pig
PRJEB22062	ERR2241683	2015	Germany	pig
PRJEB22062	ERR2241684	2015	Germany	pig

PRJEB22062	ERR2241685	2015	Germany	pig
PRJEB22062	ERR2241686	2015	Germany	chicken
PRJEB22062	ERR2241687	2015	Germany	chicken
PRJEB22062	ERR2241688	2015	Germany	chicken
PRJEB22062	ERR2241689	2015	Germany	chicken
PRJEB22062	ERR2241690	2015	Germany	chicken
PRJEB22062	ERR2241691	2015	Germany	chicken
PRJEB22062	ERR2241692	2015	Germany	chicken
PRJEB22062	ERR2241693	2015	Germany	chicken
PRJEB22062	ERR2241694	2015	Germany	chicken
PRJEB22062	ERR2241695	2015	Germany	chicken
PRJEB22062	ERR2241696	2015	Germany	chicken
PRJEB22062	ERR2241697	2015	Germany	chicken
PRJEB22062	ERR2241698	2015	Germany	chicken
PRJEB22062	ERR2241699	2015	Germany	chicken
PRJEB22062	ERR2241700	2015	Germany	chicken
PRJEB22062	ERR2241701	2015	Germany	chicken
PRJEB22062	ERR2241702	2015	Germany	chicken
PRJEB22062	ERR2241703	2015	Germany	chicken
PRJEB22062	ERR2241704	2015	Germany	chicken

PRJEB22062	ERR2241705	2015	France	pig
PRJEB22062	ERR2241706	2015	France	pig
PRJEB22062	ERR2241707	2015	France	pig
PRJEB22062	ERR2241708	2015	France	pig
PRJEB22062	ERR2241709	2015	France	pig
PRJEB22062	ERR2241710	2015	France	pig
PRJEB22062	ERR2241711	2015	France	pig
PRJEB22062	ERR2241712	2015	France	pig
PRJEB22062	ERR2241713	2015	France	pig
PRJEB22062	ERR2241714	2015	France	pig
PRJEB22062	ERR2241715	2015	France	pig
PRJEB22062	ERR2241716	2015	France	pig
PRJEB22062	ERR2241717	2015	France	pig
PRJEB22062	ERR2241718	2015	France	pig
PRJEB22062	ERR2241719	2015	France	pig
PRJEB22062	ERR2241720	2015	France	pig
PRJEB22062	ERR2241721	2015	France	pig
PRJEB22062	ERR2241722	2015	France	pig
PRJEB22062	ERR2241723	2015	France	pig
PRJEB22062	ERR2241724	2015	France	pig

PRJEB22062	ERR2241725	2015	France	chicken
PRJEB22062	ERR2241726	2015	France	chicken
PRJEB22062	ERR2241727	2015	France	chicken
PRJEB22062	ERR2241728	2015	France	chicken
PRJEB22062	ERR2241729	2015	France	chicken
PRJEB22062	ERR2241730	2015	France	chicken
PRJEB22062	ERR2241731	2015	France	chicken
PRJEB22062	ERR2241732	2015	France	chicken
PRJEB22062	ERR2241733	2015	France	chicken
PRJEB22062	ERR2241734	2015	France	chicken
PRJEB22062	ERR2241736	2015	France	chicken
PRJEB22062	ERR2241737	2015	France	chicken
PRJEB22062	ERR2241738	2015	France	chicken
PRJEB22062	ERR2241739	2015	France	chicken
PRJEB22062	ERR2241740	2015	France	chicken
PRJEB22062	ERR2241741	2015	France	chicken
PRJEB22062	ERR2241742	2015	France	chicken
PRJEB22062	ERR2241743	2015	France	chicken
PRJEB22062	ERR2241744	2015	France	chicken
PRJEB22062	ERR2241745	2015	Spain	pig

PRJEB22062	ERR2241746	2015	Spain	pig
PRJEB22062	ERR2241747	2015	Spain	chicken
PRJEB22062	ERR2241748	2015	Spain	chicken
PRJEB22062	ERR2241749	2015	Spain	chicken
PRJEB22062	ERR2241750	2015	Spain	chicken
PRJEB22062	ERR2241751	2015	Spain	pig
PRJEB22062	ERR2241752	2015	Spain	chicken
PRJEB22062	ERR2241753	2015	Spain	chicken
PRJEB22062	ERR2241754	2015	Spain	chicken
PRJEB22062	ERR2241755	2015	Spain	chicken
PRJEB22062	ERR2241756	2015	Spain	chicken
PRJEB22062	ERR2241757	2015	Spain	pig
PRJEB22062	ERR2241758	2015	Spain	chicken
PRJEB22062	ERR2241759	2015	Spain	chicken
PRJEB22062	ERR2241760	2015	Spain	chicken
PRJEB22062	ERR2241761	2015	Spain	chicken
PRJEB22062	ERR2241762	2015	Spain	chicken
PRJEB22062	ERR2241763	2015	Spain	chicken
PRJEB22062	ERR2241764	2015	Spain	chicken
PRJEB22062	ERR2241765	2015	Spain	chicken

PRJEB22062	ERR2241766	2015	Spain	pig
PRJEB22062	ERR2241767	2015	Spain	pig
PRJEB22062	ERR2241768	2015	Spain	pig
PRJEB22062	ERR2241769	2015	Spain	pig
PRJEB22062	ERR2241770	2015	Spain	chicken
PRJEB22062	ERR2241771	2015	Spain	pig
PRJEB22062	ERR2241772	2015	Spain	pig
PRJEB22062	ERR2241773	2015	Spain	pig
PRJEB22062	ERR2241774	2015	Spain	pig
PRJEB22062	ERR2241775	2015	Spain	pig
PRJEB22062	ERR2241776	2015	Spain	pig
PRJEB22062	ERR2241777	2015	Spain	pig
PRJEB22062	ERR2241778	2015	Spain	pig
PRJEB22062	ERR2241779	2015	Spain	pig
PRJEB22062	ERR2241780	2015	Spain	pig
PRJEB22062	ERR2241781	2015	Spain	pig
PRJEB22062	ERR2241782	2015	Spain	pig
PRJEB22062	ERR2241783	2015	Spain	chicken
PRJEB22062	ERR2241784	2015	Spain	chicken
PRJEB22062	ERR2241785	2015	Belgium	pig

PRJEB22062	ERR2241786	2015	Belgium	pig
PRJEB22062	ERR2241787	2015	Belgium	pig
PRJEB22062	ERR2241788	2015	Belgium	chicken
PRJEB22062	ERR2241789	2015	Belgium	pig
PRJEB22062	ERR2241790	2015	Belgium	chicken
PRJEB22062	ERR2241791	2015	Belgium	chicken
PRJEB22062	ERR2241792	2015	Belgium	pig
PRJEB22062	ERR2241793	2015	Belgium	pig
PRJEB22062	ERR2241794	2015	Belgium	chicken
PRJEB22062	ERR2241795	2015	Belgium	chicken
PRJEB22062	ERR2241796	2015	Belgium	pig
PRJEB22062	ERR2241797	2015	Belgium	pig
PRJEB22062	ERR2241798	2015	Belgium	chicken
PRJEB22062	ERR2241799	2015	Belgium	chicken
PRJEB22062	ERR2241800	2015	Belgium	pig
PRJEB22062	ERR2241801	2015	Belgium	chicken
PRJEB22062	ERR2241802	2015	Belgium	chicken
PRJEB22062	ERR2241803	2015	Belgium	chicken
PRJEB22062	ERR2241804	2015	Belgium	pig
PRJEB22062	ERR2241805	2015	Belgium	chicken

PRJEB22062	ERR2241806	2015	Belgium	chicken
PRJEB22062	ERR2241807	2015	Belgium	chicken
PRJEB22062	ERR2241808	2015	Belgium	chicken
PRJEB22062	ERR2241809	2015	Belgium	chicken
PRJEB22062	ERR2241810	2015	Belgium	pig
PRJEB22062	ERR2241811	2015	Belgium	pig
PRJEB22062	ERR2241812	2015	Belgium	pig
PRJEB22062	ERR2241813	2015	Belgium	pig
PRJEB22062	ERR2241814	2015	Belgium	pig
PRJEB22062	ERR2241815	2015	Belgium	chicken
PRJEB22062	ERR2241816	2015	Belgium	pig
PRJEB22062	ERR2241817	2015	Belgium	pig
PRJEB22062	ERR2241818	2015	Belgium	chicken
PRJEB22062	ERR2241819	2015	Belgium	chicken
PRJEB22062	ERR2241820	2015	Belgium	pig
PRJEB22062	ERR2241821	2015	Belgium	pig
PRJEB22062	ERR2241822	2015	Belgium	pig
PRJEB22062	ERR2241823	2015	Belgium	chicken
PRJEB22062	ERR2241824	2015	Belgium	chicken
PRJEB22062	ERR2241825	2015	Belgium	pig

PRJEB22062	ERR2241826	2015	Belgium	pig
PRJEB22062	ERR2241827	2015	Italy	pig
PRJEB22062	ERR2241828	2015	Italy	chicken
PRJEB22062	ERR2241829	2015	Italy	pig
PRJEB22062	ERR2241830	2015	Italy	pig
PRJEB22062	ERR2241831	2015	Italy	pig
PRJEB22062	ERR2241832	2015	Italy	pig
PRJEB22062	ERR2241833	2015	Italy	pig
PRJEB22062	ERR2241834	2015	Italy	pig
PRJEB22062	ERR2241835	2015	Italy	pig
PRJEB22062	ERR2241836	2015	Italy	pig
PRJEB22062	ERR2241837	2015	Italy	pig
PRJEB22062	ERR2241838	2015	Italy	pig
PRJEB22062	ERR2241839	2015	Italy	pig
PRJEB22062	ERR2241840	2015	Italy	pig
PRJEB22062	ERR2241841	2015	Italy	pig
PRJEB22062	ERR2241842	2015	Italy	pig
PRJEB22062	ERR2241843	2015	Italy	pig
PRJEB22062	ERR2241844	2015	Italy	pig
PRJEB22062	ERR2241845	2015	Italy	pig

PRJEB22062	ERR2241846	2015	Italy	pig
PRJEB22062	ERR2241847	2015	Italy	pig
PRJEB22062	ERR2241848	2015	Italy	chicken
PRJEB22062	ERR2241849	2015	Italy	chicken
PRJEB22062	ERR2241850	2015	Italy	chicken
PRJEB22062	ERR2241851	2015	Italy	chicken
PRJEB22062	ERR2241852	2015	Italy	chicken
PRJEB22062	ERR2241853	2015	Italy	chicken
PRJEB22062	ERR2241854	2015	Italy	chicken
PRJEB22062	ERR2241855	2015	Italy	chicken
PRJEB22062	ERR2241856	2015	Italy	chicken
PRJEB22062	ERR2241857	2015	Italy	chicken
PRJEB22062	ERR2241858	2015	Italy	chicken
PRJEB22062	ERR2241859	2015	Italy	chicken
PRJEB22062	ERR2241860	2015	Italy	chicken
PRJEB22062	ERR2241861	2015	Italy	chicken
PRJEB22062	ERR2241862	2015	Italy	chicken
PRJEB22062	ERR2241863	2015	Italy	chicken
PRJEB22062	ERR2241864	2015	Italy	chicken
PRJEB22062	ERR2241865	2015	Italy	chicken

PRJEB22062	ERR2241866	2015	Italy	chicken
PRJEB22062	ERR2241867	2015	Poland	pig
PRJEB22062	ERR2241868	2015	Poland	pig
PRJEB22062	ERR2241869	2015	Poland	pig
PRJEB22062	ERR2241870	2015	Poland	pig
PRJEB22062	ERR2241871	2015	Poland	pig
PRJEB22062	ERR2241872	2015	Poland	pig
PRJEB22062	ERR2241873	2015	Poland	pig
PRJEB22062	ERR2241874	2015	Poland	pig
PRJEB22062	ERR2241875	2015	Poland	pig
PRJEB22062	ERR2241876	2015	Poland	pig
PRJEB22062	ERR2241877	2015	Poland	pig
PRJEB22062	ERR2241878	2015	Poland	pig
PRJEB22062	ERR2241879	2015	Poland	pig
PRJEB22062	ERR2241880	2015	Poland	pig
PRJEB22062	ERR2241881	2015	Poland	pig
PRJEB22062	ERR2241882	2015	Poland	pig
PRJEB22062	ERR2241883	2015	Poland	pig
PRJEB22062	ERR2241884	2015	Poland	pig
PRJEB22062	ERR2241885	2015	Poland	pig

PRJEB22062	ERR2241886	2015	Poland	pig
PRJEB22062	ERR2241887	2015	Poland	chicken
PRJEB22062	ERR2241888	2015	Poland	chicken
PRJEB22062	ERR2241889	2015	Poland	chicken
PRJEB22062	ERR2241890	2015	Poland	chicken
PRJEB22062	ERR2241891	2015	Poland	chicken
PRJEB22062	ERR2241892	2015	Poland	chicken
PRJEB22062	ERR2241893	2015	Poland	chicken
PRJEB22062	ERR2241894	2015	Poland	chicken
PRJEB22062	ERR2241895	2015	Poland	chicken
PRJEB22062	ERR2241896	2015	Poland	chicken
PRJEB22062	ERR2241897	2015	Poland	chicken
PRJEB22062	ERR2241898	2015	Poland	chicken
PRJEB22062	ERR2241899	2015	Poland	chicken
PRJEB22062	ERR2241900	2015	Poland	chicken
PRJEB22062	ERR2241901	2015	Poland	chicken
PRJEB22062	ERR2241902	2015	Poland	chicken
PRJEB22062	ERR2241903	2015	Poland	chicken
PRJEB22062	ERR2241904	2015	Poland	chicken
PRJEB22062	ERR2241905	2015	Poland	chicken

PRJEB22062	ERR2241906	2015	Poland	chicken
PRJEB22062	ERR2241907	2015	Denmark	chicken
PRJEB22062	ERR2241908	2015	Denmark	pig
PRJEB22062	ERR2241909	2015	Denmark	chicken
PRJEB22062	ERR2241910	2015	Denmark	pig
PRJEB22062	ERR2241911	2015	Denmark	chicken
PRJEB22062	ERR2241912	2015	Denmark	pig
PRJEB22062	ERR2241913	2015	Denmark	chicken
PRJEB22062	ERR2241914	2015	Denmark	pig
PRJEB22062	ERR2241915	2015	Denmark	chicken
PRJEB22062	ERR2241916	2015	Denmark	pig
PRJEB22062	ERR2241917	2015	Denmark	chicken
PRJEB22062	ERR2241918	2015	Denmark	pig
PRJEB22062	ERR2241919	2015	Denmark	chicken
PRJEB22062	ERR2241920	2015	Denmark	pig
PRJEB22062	ERR2241921	2015	Denmark	chicken
PRJEB22062	ERR2241922	2015	Denmark	pig
PRJEB22062	ERR2241923	2015	Denmark	chicken
PRJEB22062	ERR2241924	2015	Denmark	pig
PRJEB22062	ERR2241925	2015	Denmark	chicken

PRJEB22062	ERR2241926	2015	Denmark	pig
PRJEB22062	ERR2241927	2015	Denmark	chicken
PRJEB22062	ERR2241928	2015	Denmark	pig
PRJEB22062	ERR2241929	2015	Denmark	chicken
PRJEB22062	ERR2241930	2015	Denmark	pig
PRJEB22062	ERR2241931	2015	Denmark	chicken
PRJEB22062	ERR2241932	2015	Denmark	pig
PRJEB22062	ERR2241933	2015	Denmark	chicken
PRJEB22062	ERR2241934	2015	Denmark	pig
PRJEB22062	ERR2241935	2015	Denmark	chicken
PRJEB22062	ERR2241936	2015	Denmark	pig
PRJEB22062	ERR2241937	2015	Denmark	chicken
PRJEB22062	ERR2241938	2015	Denmark	pig
PRJEB22062	ERR2241939	2015	Denmark	chicken
PRJEB22062	ERR2241940	2015	Denmark	pig
PRJEB22062	ERR2241941	2015	Denmark	chicken
PRJEB22062	ERR2241942	2015	Denmark	pig
PRJEB22062	ERR2241943	2015	Denmark	chicken
PRJEB22062	ERR2241944	2015	Denmark	pig
PRJEB22062	ERR2241945	2015	Denmark	chicken

PRJEB22062	ERR2241946	2015	Denmark	pig
PRJEB22062	ERR2241947	2015	Bulgaria	pig
PRJEB22062	ERR2241948	2015	Bulgaria	pig
PRJEB22062	ERR2241949	2015	Bulgaria	pig
PRJEB22062	ERR2241950	2015	Bulgaria	chicken
PRJEB22062	ERR2241951	2015	Bulgaria	pig
PRJEB22062	ERR2241952	2015	Bulgaria	chicken
PRJEB22062	ERR2241953	2015	Bulgaria	chicken
PRJEB22062	ERR2241954	2015	Bulgaria	chicken
PRJEB22062	ERR2241955	2015	Bulgaria	chicken
PRJEB22062	ERR2241956	2015	Bulgaria	chicken
PRJEB22062	ERR2241957	2015	Bulgaria	chicken
PRJEB22062	ERR2241958	2015	Bulgaria	pig
PRJEB22062	ERR2241959	2015	Bulgaria	pig
PRJEB22062	ERR2241960	2015	Bulgaria	pig
PRJEB22062	ERR2241961	2015	Bulgaria	pig
PRJEB22062	ERR2241962	2015	Bulgaria	pig
PRJEB22062	ERR2241963	2015	Bulgaria	pig
PRJEB22062	ERR2241964	2015	Bulgaria	pig
PRJEB22062	ERR2241965	2015	Bulgaria	pig

PRJEB22062	ERR2241966	2015	Bulgaria	pig
PRJEB22062	ERR2241967	2015	Bulgaria	pig
PRJEB22062	ERR2241968	2015	Bulgaria	pig
PRJEB22062	ERR2241969	2015	Bulgaria	pig
PRJEB22062	ERR2241970	2015	Bulgaria	pig
PRJEB22062	ERR2241971	2015	Bulgaria	chicken
PRJEB22062	ERR2241972	2015	Bulgaria	chicken
PRJEB22062	ERR2241973	2015	Bulgaria	pig
PRJEB22062	ERR2241974	2015	Bulgaria	pig
PRJEB22062	ERR2241975	2015	Bulgaria	pig
PRJEB22062	ERR2241976	2015	Bulgaria	pig
PRJEB22062	ERR2241977	2015	Bulgaria	chicken
PRJEB22062	ERR2241978	2015	Bulgaria	chicken
PRJEB22062	ERR2241979	2015	Bulgaria	chicken
PRJEB22062	ERR2241980	2015	Bulgaria	chicken
PRJEB22062	ERR2241981	2015	Bulgaria	chicken
PRJEB22062	ERR2241982	2015	Bulgaria	chicken
PRJEB22062	ERR2241983	2015	Bulgaria	chicken
PRJEB22062	ERR2241984	2015	Bulgaria	chicken
PRJEB22062	ERR2241985	2015	Bulgaria	chicken

PRJEB22062	ERR2241986	2015	Bulgaria	chicken
PRJEB22062	ERR2244943	2015	Netherlands	pig
PRJEB22062	ERR2245331	2015	Netherlands	pig
PRJEB22062	ERR2245443	2015	Netherlands	pig
PRJEB22062	ERR2245444	2015	Netherlands	pig
PRJEB22062	ERR2245445	2015	Netherlands	pig
PRJEB22062	ERR2245446	2015	Netherlands	pig
PRJEB22062	ERR2245447	2015	Netherlands	pig
PRJEB22062	ERR2245448	2015	Netherlands	pig
PRJEB22062	ERR2245449	2015	Netherlands	pig
PRJEB22062	ERR2245450	2015	Netherlands	pig
PRJEB22062	ERR2245451	2015	Netherlands	pig
PRJEB22062	ERR2245452	2015	Netherlands	pig
PRJEB22062	ERR2245453	2015	Germany	pig
PRJEB22062	ERR2245454	2015	Germany	pig
PRJEB22062	ERR2245455	2015	Germany	chicken
PRJEB22062	ERR2245456	2015	Spain	pig
PRJEB22062	ERR2245457	2015	Belgium	pig
PRJEB22062	ERR2245458	2015	Belgium	pig
PRJEB22062	ERR2245459	2015	Belgium	pig

PRJEB22062	ERR2245460	2015	Belgium	pig
PRJEB22062	ERR2245461	2015	Belgium	pig
PRJEB22062	ERR2245462	2015	Belgium	pig
PRJEB22062	ERR2245463	2015	Belgium	pig
PRJEB22062	ERR2245464	2015	Belgium	pig
PRJEB22062	ERR2245465	2015	Belgium	pig
PRJEB22062	ERR2245466	2015	Belgium	pig
PRJEB22062	ERR2245467	2015	Belgium	pig
PRJEB22062	ERR2245468	2015	Italy	pig
PRJEB22062	ERR2245469	2015	Italy	pig
PRJEB22062	ERR2245470	2015	Italy	pig
PRJEB22062	ERR2245471	2015	Italy	pig
PRJEB22062	ERR2245472	2015	Italy	pig
PRJEB22062	ERR2245473	2015	Italy	pig
PRJEB22062	ERR2245474	2015	Italy	pig
PRJEB22062	ERR2245475	2015	Italy	pig
PRJEB22062	ERR2245476	2015	Italy	pig
PRJEB22062	ERR2245477	2015	Italy	pig
PRJEB22062	ERR2245478	2015	Italy	pig
PRJEB22062	ERR2245479	2015	Poland	pig

PRJEB22062	ERR2245480	2015	Poland	pig
PRJEB22062	ERR2245481	2015	Poland	pig
PRJEB22062	ERR2245482	2015	Poland	pig
PRJEB22062	ERR2245483	2015	Poland	pig
PRJEB22062	ERR2245484	2015	Poland	pig
PRJEB22062	ERR2245485	2015	Poland	pig
PRJEB22062	ERR2245486	2015	Poland	pig
PRJEB22062	ERR2245487	2015	Poland	pig
PRJEB22062	ERR2245488	2015	Poland	pig
PRJEB22062	ERR2245489	2015	Poland	pig
PRJEB22062	ERR2245490	2015	Denmark	pig
PRJEB22062	ERR2245491	2015	Bulgaria	pig
PRJEB22062	ERR2245492	2015	Bulgaria	pig
PRJEB22062	ERR2245493	2015	Bulgaria	pig
PRJEB22062	ERR2245494	2015	Bulgaria	pig
PRJEB39685	ERR4398787	not provided	Netherlands	cattle
PRJEB39685	ERR4398788	not provided	Netherlands	cattle
PRJEB39685	ERR4398789	not provided	Netherlands	cattle
PRJEB39685	ERR4398790	not provided	Netherlands	cattle
PRJEB39685	ERR4398791	not provided	Netherlands	cattle

PRJEB39685	ERR4398792	not provided	Netherlands	cattle
PRJEB39685	ERR4398793	not provided	Germany	cattle
PRJEB39685	ERR4398794	not provided	Germany	cattle
PRJEB39685	ERR4398795	not provided	Germany	cattle
PRJEB39685	ERR4398796	not provided	Germany	cattle
PRJEB39685	ERR4398797	not provided	Germany	cattle
PRJEB39685	ERR4398798	not provided	Germany	cattle
PRJEB39685	ERR4398799	not provided	Germany	cattle
PRJEB39685	ERR4398800	not provided	Germany	cattle
PRJEB39685	ERR4398801	not provided	Germany	cattle
PRJEB39685	ERR4398802	not provided	Germany	cattle
PRJEB39685	ERR4398803	not provided	Germany	cattle
PRJEB39685	ERR4398804	not provided	Germany	cattle
PRJEB39685	ERR4398805	not provided	Germany	cattle
PRJEB39685	ERR4398806	not provided	Germany	cattle
PRJEB39685	ERR4398807	not provided	Germany	cattle
PRJEB39685	ERR4398808	not provided	France	cattle
PRJEB39685	ERR4398809	not provided	France	cattle
PRJEB39685	ERR4398810	not provided	France	cattle
PRJEB39685	ERR4398811	not provided	France	cattle

PRJEB39685	ERR4398812	not provided	France	cattle
PRJEB39685	ERR4398813	not provided	France	cattle
PRJEB39685	ERR4398814	not provided	France	cattle
PRJEB39685	ERR4398815	not provided	France	cattle
PRJEB39685	ERR4398816	not provided	Germany	turkey
PRJEB39685	ERR4398817	not provided	Germany	turkey
PRJEB39685	ERR4398818	not provided	Germany	turkey
PRJEB39685	ERR4398819	not provided	Germany	turkey
PRJEB39685	ERR4398820	not provided	Germany	turkey
PRJEB39685	ERR4398821	not provided	Germany	turkey
PRJEB39685	ERR4398822	not provided	Germany	turkey
PRJEB39685	ERR4398823	not provided	Germany	turkey
PRJEB39685	ERR4398824	not provided	Germany	turkey
PRJEB39685	ERR4398825	not provided	France	turkey
PRJEB39685	ERR4398826	not provided	France	turkey
PRJEB39685	ERR4398827	not provided	France	turkey
PRJEB39685	ERR4398828	not provided	France	turkey
PRJEB39685	ERR4398829	not provided	France	turkey
PRJEB39685	ERR4398830	not provided	France	turkey
PRJEB39685	ERR4398831	not provided	France	turkey

PRJEB39685	ERR4398832	not provided	France	turkey
PRJEB22062	ERR2241735	2015	France	chicken
PRJEB39685	ERR4398741	not provided	Germany	turkey
PRJEB39685	ERR4398735	not provided	Germany	turkey
PRJEB39685	ERR4398736	not provided	Germany	turkey
PRJEB39685	ERR4398737	not provided	Germany	turkey
PRJEB39685	ERR4398780	not provided	Germany	turkey
PRJEB39685	ERR4398738	not provided	Germany	turkey
PRJEB39685	ERR4398739	not provided	Germany	turkey
PRJEB39685	ERR4398740	not provided	Germany	turkey
PRJEB39685	ERR4398742	not provided	Germany	turkey
PRJEB39685	ERR4398743	not provided	Germany	turkey
PRJEB39685	ERR4398781	not provided	Germany	turkey
PRJEB39685	ERR4398744	not provided	Germany	turkey
PRJEB39685	ERR4398746	not provided	Germany	turkey
PRJEB39685	ERR4398745	not provided	Germany	turkey
PRJEB39685	ERR4398762	not provided	Germany	cattle
PRJEB39685	ERR4398763	not provided	Germany	cattle
PRJEB39685	ERR4398760	not provided	Germany	cattle
PRJEB39685	ERR4398764	not provided	Germany	cattle

PRJEB39685	ERR4398765	not provided	Germany	cattle
PRJEB39685	ERR4398759	not provided	Germany	cattle
PRJEB39685	ERR4398761	not provided	Germany	cattle
PRJEB39685	ERR4398716	not provided	Spain	turkey
PRJEB39685	ERR4398720	not provided	Spain	turkey
PRJEB39685	ERR4398719	not provided	Spain	turkey
PRJEB39685	ERR4398718	not provided	Spain	turkey
PRJEB39685	ERR4398783	not provided	Spain	turkey
PRJEB39685	ERR4398732	not provided	Spain	turkey
PRJEB39685	ERR4398714	not provided	Spain	turkey
PRJEB39685	ERR4398713	not provided	Spain	turkey
PRJEB39685	ERR4398717	not provided	Spain	turkey
PRJEB39685	ERR4398721	not provided	Spain	turkey
PRJEB39685	ERR4398784	not provided	Spain	turkey
PRJEB39685	ERR4398785	not provided	Spain	turkey
PRJEB39685	ERR4398726	not provided	Spain	turkey
PRJEB39685	ERR4398725	not provided	Spain	turkey
PRJEB39685	ERR4398724	not provided	Spain	turkey
PRJEB39685	ERR4398782	not provided	Spain	turkey
PRJEB39685	ERR4398723	not provided	Spain	turkey

PRJEB39685	ERR4398730	not provided	Spain	turkey
PRJEB39685	ERR4398786	not provided	Spain	turkey
PRJEB39685	ERR4398729	not provided	Spain	turkey
PRJEB39685	ERR4398728	not provided	Spain	turkey
PRJEB39685	ERR4398715	not provided	Spain	turkey
PRJEB39685	ERR4398722	not provided	Spain	turkey
PRJEB39685	ERR4398727	not provided	Spain	turkey
PRJEB39685	ERR4398731	not provided	Spain	turkey
PRJEB39685	ERR4398734	not provided	Spain	turkey
PRJEB39685	ERR4398733	not provided	Spain	turkey
PRJEB39685	ERR4398701	not provided	France	turkey
PRJEB39685	ERR4398702	not provided	France	turkey
PRJEB39685	ERR4398703	not provided	France	turkey
PRJEB39685	ERR4398704	not provided	France	turkey
PRJEB39685	ERR4398705	not provided	France	turkey
PRJEB39685	ERR4398706	not provided	France	turkey
PRJEB39685	ERR4398707	not provided	France	turkey
PRJEB39685	ERR4398708	not provided	France	turkey
PRJEB39685	ERR4398709	not provided	France	turkey
PRJEB39685	ERR4398710	not provided	France	turkey

PRJEB39685	ERR4398711	not provided	France	turkey
PRJEB39685	ERR4398712	not provided	France	turkey
PRJEB39685	ERR4398747	not provided	France	cattle
PRJEB39685	ERR4398748	not provided	France	cattle
PRJEB39685	ERR4398749	not provided	France	cattle
PRJEB39685	ERR4398750	not provided	France	cattle
PRJEB39685	ERR4398751	not provided	France	cattle
PRJEB39685	ERR4398752	not provided	France	cattle
PRJEB39685	ERR4398753	not provided	France	cattle
PRJEB39685	ERR4398754	not provided	France	cattle
PRJEB39685	ERR4398755	not provided	France	cattle
PRJEB39685	ERR4398756	not provided	France	cattle
PRJEB39685	ERR4398757	not provided	France	cattle
PRJEB39685	ERR4398758	not provided	France	cattle
PRJEB39685	ERR4398766	not provided	Netherlands	cattle
PRJEB39685	ERR4398767	not provided	Netherlands	cattle
PRJEB39685	ERR4398768	not provided	Netherlands	cattle
PRJEB39685	ERR4398769	not provided	Netherlands	cattle
PRJEB39685	ERR4398770	not provided	Netherlands	cattle
PRJEB39685	ERR4398771	not provided	Netherlands	cattle

PRJEB39685	ERR4398772	not provided	Netherlands	cattle
PRJEB39685	ERR4398773	not provided	Netherlands	cattle
PRJEB39685	ERR4398774	not provided	Netherlands	cattle
PRJEB39685	ERR4398775	not provided	Netherlands	cattle
PRJEB39685	ERR4398776	not provided	Netherlands	cattle
PRJEB39685	ERR4398777	not provided	Netherlands	cattle
PRJEB39685	ERR4398778	not provided	Netherlands	cattle
PRJEB39685	ERR4398779	not provided	Netherlands	cattle
