# Supplementary material for: Farming practices exert selection pressures on the resistome of natural populations of house mice
Source: Nat Commun. 2026 Aug 5;17:7848. doi: 10.1038/s41467-026-76403-9 (PMC13443167; doi:10.1038/s41467-026-76403-9)
Supplement: Supplementary file 2 [file 41467_2026_76403_MOESM2_ESM.pdf]

nature portfolio

Corresponding author(s): Víctor Hugo Jarquín-Díaz

Last updated by author(s): Jun 15, 2026

Reporting Summary

Nature Portfolio wishes to improve the reproducibility of the work that we publish. This form provides structure for consistency and transparency in reporting. For further information on Nature Portfolio policies, see our Editorial Policies and the Editorial Policy Checklist.

Statistics

For all statistical analyses, confirm that the following items are present in the figure legend, table legend, main text, or Methods section.

n/a	Confirmed	
☐	☒	The exact sample size (n) for each experimental group/condition, given as a discrete number and unit of measurement
☐	☒	A statement on whether measurements were taken from distinct samples or whether the same sample was measured repeatedly
☐	☒	The statistical test(s) used AND whether they are one- or two-sided<br>*Only common tests should be described solely by name; describe more complex techniques in the Methods section.*
☐	☒	A description of all covariates tested
☐	☒	A description of any assumptions or corrections, such as tests of normality and adjustment for multiple comparisons
☐	☒	A full description of the statistical parameters including central tendency (e.g. means) or other basic estimates (e.g. regression coefficient) AND variation (e.g. standard deviation) or associated estimates of uncertainty (e.g. confidence intervals)
☐	☒	For null hypothesis testing, the test statistic (e.g. F, t, r) with confidence intervals, effect sizes, degrees of freedom and P value noted<br>*Give P values as exact values whenever suitable.*
☐	☒	For Bayesian analysis, information on the choice of priors and Markov chain Monte Carlo settings
☐	☒	For hierarchical and complex designs, identification of the appropriate level for tests and full reporting of outcomes
☐	☒	Estimates of effect sizes (e.g. Cohen's d, Pearson's r), indicating how they were calculated

Our web collection on statistics for biologists contains articles on many of the points above.

Software and code

Policy information about availability of computer code

Data collection	No software was used to collect the data. All environmental layers used for the modelling were obtained from open data available at Copernicus (EU Space programme on Earth observation), GeoBasis-DE, Thünen Atlas, and DWD.
Data analysis	All the code used for visualization and statistical analysis have been deposited in Zenodo under the DOI 10.5281/zenodo.20624924

For manuscripts utilizing custom algorithms or software that are central to the research but not yet described in published literature, software must be made available to editors and reviewers. We strongly encourage code deposition in a community repository (e.g. GitHub). See the Nature Portfolio guidelines for submitting code & software for further information.

Data

Policy information about availability of data

All manuscripts must include a data availability statement. This statement should provide the following information, where applicable:

- Accession codes, unique identifiers, or web links for publicly available datasets
- A description of any restrictions on data availability
- For clinical datasets or third party data, please ensure that the statement adheres to our policy

All raw sequence data are deposited and available online at the ENA with accession number PRJEB102564. Additional source data for the analysis have been deposited in Zenodo and it is available at 10.5281/zenodo.20624924.

Research involving human participants, their data, or biological material

Policy information about studies with human participants or human data. See also policy information about sex, gender (identity/presentation), and sexual orientation and race, ethnicity and racism.

Reporting on sex and gender	No human participants or biological material derived from humans was used in the study
Reporting on race, ethnicity, or other socially relevant groupings	No human participants or biological material derived from humans was used in the study
Population characteristics	No human participants or biological material derived from humans was used in the study
Recruitment	No human participants or biological material derived from humans was used in the study
Ethics oversight	No human participants or biological material derived from humans was used in the study

Note that full information on the approval of the study protocol must also be provided in the manuscript.

Field-specific reporting

Please select the one below that is the best fit for your research. If you are not sure, read the appropriate sections before making your selection.

☐ Life sciences ☐ Behavioural & social sciences ☒ Ecological, evolutionary & environmental sciences

For a reference copy of the document with all sections, see nature.com/documents/nr-reporting-summary-flat.pdf

Ecological, evolutionary & environmental sciences study design

All studies must disclose on these points even when the disclosure is negative.

Study description	We analysed 875 gut metagenomes from wild house mice (Mus musculus) collected from German farms between 2016 and 2022 to identify environmental and host determinant of ARG ocurrence.
Research sample	Colon content metagenomes from 395 male and 479 female wild house mice (Mus musculus) were used for the analysis.
Sampling strategy	No sample size calculation was perfomed.
Data collection	Data was collected in September between 2016 and 2022, with 2020 as the only missing year due to COVID-19 restrictions
Timing and spatial scale	Sampling was done once per year in September corresponding to late summer -early autumn season
Data exclusions	Samples with less than 1000 reads assigned to bacteria were removed from the analysis
Reproducibility	All pipelines for microbiome, ARG determination were standardized, evaluated and documented to ensure reproducibility. All the analysis was conducted with well documented pipelines.
Randomization	DNA of the samples was extracted, and distributed in 96 well plates for sequencing after randomization considering year of collection,
Blinding	Alphanumeric IDs were given to each mouse. The IDs did not include any information that could link samples to their characteristics

Did the study involve field work? ☒ Yes ☐ No

Field work, collection and transport

Field conditions	We visited farms during late summer and early autumn. Traps were set in places were mice inhabit within the farms and collected overnight.
Location	We conducted the study in Germany in the federal states of Berlin, Brandenburg, Bavaria and Mecklenburg-Vorpommern.
Access & import/export	We collected mice from farms with the permission of the owners.
Disturbance	As samples were collected from farms and house mice are consider pest animals, we did not disturbed any other wildlife species in the process of sample collection.

Reporting for specific materials, systems and methods

We require information from authors about some types of materials, experimental systems and methods used in many studies. Here, indicate whether each material, system or method listed is relevant to your study. If you are not sure if a list item applies to your research, read the appropriate section before selecting a response.

Materials & experimental systems

n/a	Involved in the study
☐	☐ Antibodies
☐	☐ Eukaryotic cell lines
☐	☐ Palaeontology and archaeology
☐	☒ Animals and other organisms
☐	☐ Clinical data
☐	☐ Dual use research of concern
☐	☐ Plants

Methods

n/a	Involved in the study
☒	☐ ChIP-seq
☒	☐ Flow cytometry
☒	☐ MRI-based neuroimaging

Antibodies

Antibodies used	NA
Validation	NA

Eukaryotic cell lines

Policy information about cell lines and Sex and Gender in Research

Cell line source(s)	NA
Authentication	NA
Mycoplasma contamination	NA
Commonly misidentified lines (See ICLAC register)	NA

Palaeontology and Archaeology

Specimen provenance	NA
Specimen deposition	NA
Dating methods	NA

☐ Tick this box to confirm that the raw and calibrated dates are available in the paper or in Supplementary Information.

Ethics oversight	NA

Note that full information on the approval of the study protocol must also be provided in the manuscript.

Animals and other research organisms

Policy information about studies involving animals; ARRIVE guidelines recommended for reporting animal research, and Sex and Gender in Research

Laboratory animals	NA
Wild animals	Mice were captured from farms in live traps, housed individually in cages until sample collection. Mice were euthanized maximum 24h after collection by overdoses of isoflurane.
Reporting on sex	Results apply to both sexes, no distinction was done
Field-collected samples	No further lab work than dissection for colon content collection was done with the wild mice.

Ethics oversight	Animal experiments were done under the licenses 2347/35/2014 and 2347/2021 issued by LAVG.

Note that full information on the approval of the study protocol must also be provided in the manuscript.

Clinical data

Policy information about clinical studies

All manuscripts should comply with the ICMJE guidelines for publication of clinical research and a completed CONSORT checklist must be included with all submissions.

Clinical trial registration	NA
Study protocol	NA
Data collection	NA
Outcomes	NA

Dual use research of concern

Policy information about dual use research of concern

Hazards

Could the accidental, deliberate or reckless misuse of agents or technologies generated in the work, or the application of information presented in the manuscript, pose a threat to:

No	Yes	
☒	☐	Public health
☒	☐	National security
☒	☐	Crops and/or livestock
☒	☐	Ecosystems
☒	☐	Any other significant area

Experiments of concern

Does the work involve any of these experiments of concern:

No	Yes	
☒	☐	Demonstrate how to render a vaccine ineffective
☒	☐	Confer resistance to therapeutically useful antibiotics or antiviral agents
☒	☐	Enhance the virulence of a pathogen or render a nonpathogen virulent
☒	☐	Increase transmissibility of a pathogen
☒	☐	Alter the host range of a pathogen
☒	☐	Enable evasion of diagnostic/detection modalities
☒	☐	Enable the weaponization of a biological agent or toxin
☒	☐	Any other potentially harmful combination of experiments and agents

Plants

Seed stocks	NA
Novel plant genotypes	NA
Authentication	NA
