# Supplementary material for: Farming practices exert selection pressures on the resistome of natural populations of house mice
Source: Nat Commun. 2026 Aug 5;17:7848. doi: 10.1038/s41467-026-76403-9 (PMC13443167; doi:10.1038/s41467-026-76403-9)
Supplement: Supplementary file 3 [file 41467_2026_76403_MOESM3_ESM.pdf]

nature portfolio

Peer Review File

Farming practices exert selection pressures on the resistome of natural populations of house mice

Corresponding Author: Dr Víctor Hugo Jarquín-Díaz

This file contains all reviewer reports in order by version, followed by all author rebuttals in order by version.

Version 0:

Reviewer comments:

Reviewer #1

(Remarks to the Author)
This is a very well-written and thorough examination of the gut resistomes of house mice from different locations in Germany. The study compares the composition of these resistomes to the environments in which the mice were captured, as well as to resistomes drawn from broader databases representing different farm animals from studies conducted in various parts of the world. The work is good and provides valuable insights into the relationships between environmental exposures, including exposure to different types of domesticated farm animals and the prevalence, types, and diversity of resistance genes acquired, and resistomes of nearby mice.

Assuming the assuming that defend my comments below, the resulting observations say a great deal about animal–animal transmission (not as much as many assume) and about how the broader environment surrounding animals influences resistance found in incidental species that are in proximity to other animals potentially exposed to antimicrobials. The authors identify particular associations between mice and pigs and show that herd density is an important factor, both of which are consistent with findings from other animal–animal systems. However, the work here is especially rigorous, and the data are more powerful than in previous studies. I therefore recommend publication with relatively minor revisions, assuming the authors can address my comments below.

My main concern with the paper relates to the title and how the data are discussed. This paper is fundamentally about farms, and the use of the term "anthropogenic" is misleading because the study does not examine urban or non-agricultural environments. As such, the title should be revised to something along the lines of:

“Anthropogenic land use exerts selection pressures on the resistome of non-domesticated rodents in farm environments” (or something like this)

Having lived on a farm, I feel strongly that mice on farms are not truly “wild” but are instead part of the farm system. They are not domesticated rodents, but they experience substantial human–animal contact. Thus, describing them as “wild” is misleading. Moreover, the authors frequently use the term “house mice,” which contradicts the use of “wild.” Using the term “house mice” is appropriate, but “wild” is not. This is not merely a semantic issue: a truly wild rodent with limited domestic exposure (for example, in a savannah ecosystem) would, in theory, have a very different resistome, and this is not the system studied here.

Additionally, this study primarily examines farm systems, and the title is too general. As currently written, it implies that the work encompasses all environments with human impact, which is not the case. It would be clearer and more accurate for readers if “farm” or “agricultural” were included in the title and consistently stated throughout the manuscript. This change would not diminish the quality of the work but would instead present it more clearly in relation to what was actually studied.

Beyond these points, I have a few additional comments:

1. Figure 2 and associated discussion: The authors report differences between the periods 2016–2019 and 2019–2022, suggesting temporal changes. However, these periods also correspond to two distinct sampling campaigns. It is therefore possible that the observed differences reflect differences between campaigns rather than true temporal trends. Please examine potential differences in the sampling campaigns and justify the interpretation that these changes are time-related.

Additionally, herd density may have changed over time and could explain the observed differences. Please discuss herd density in relation to time within the context of these data.

2. Figure 3: Nothing in this figure indicates an urban context. This figure and its associated discussion exemplify the need for clearer editing to emphasize that this study focuses primarily on farm environments rather than urban settings.

3. Figure 5 (resistome diversity comparisons): Could the lower resistome diversity observed in mice, compared to other animals, be a consequence of differences in sampling and extraction methods? The mouse samples were collected and processed uniformly, whereas the comparison data were drawn from databases with no guarantee of comparable sampling depth, collection methods, or processing protocols. I am surprised that mouse resistomes appear to be relatively low in diversity; I would have expected them to resemble those of birds, which tend to be highly diverse. Please further justify the methods and interpretations here. It is possible that the observed low ARG diversity reflects bias, especially given that these mice were largely sampled from farms, which your own data suggest impose selective pressures related to herd density. That same selection pressure may also influence ARG diversity in mice.

4. Pages 698–705: Could the observed patterns be influenced by sampling limitations? The methods used to collect and analyze the mice may reflect the inherently less diverse environments associated with farm ecosystems, which may select for lower diversity overall. This further underscores the importance of removing references to “wild” throughout the manuscript. Please consider this point carefully in the broader narrative.

(Remarks on code availability)

Reviewer #2

(Remarks to the Author)
This is a really interesting paper examining AMR using gut samples from wild house mice (Mus musculus) from German farms with metagenomes. The data were analyses using a range of techniques, but the traits revealed that agricultural land use and exposure to livestock increased the occurrence of mobile antimicrobial resistance genes. This is a well well-designed with important findings as to where the source of AMR in wildlife derives. The statistical analyses linking the host and environmental variables is really interesting. Other comments are outlined below.

L54 Are the factors know any better in non-human dominated landscapes? In Europe, even ‘wild’ systems are all human dominated.

L103-104 And in the Introduction in general- human activity is driving this process so don’t frame it as wildlife being the source of the issue or blame for the problem. In order to be accurate, soften the language used regarding the role of AMR risk to public health.

L130 Animals found in human dominated landscapes make us sick in respect to zoonoses
This citation may be useful for L112-130:
Gibb, R., Redding, D.W., Chin, K.Q. et al. Zoonotic host diversity increases in human-dominated ecosystems. Nature 584, 398–402 (2020). https://doi.org/10.1038/s41586-020-2562-8

L132-144: Although the content in this section is broadly accurate is it poorly referenced. Please see below some ideas for zoonoses emergence. I believe that your data is perfectly placed to show an analogous situation for AMR linked to zoonoses but the framing needs to be correct. It was first the case with zoonoses but now you’re demonstrating a similar link with AMR.
https://doi.org/10.1073/pnas.1208059110
https://doi.org/10.1111/zph.12489
https://doi.org/10.1016/ S2542-5196(21)00031-0

L319 What were the random and fixed effects? Could you include a Table that outlines that data and categories attributes e.g. response or explanatory, nature of data, fixed/random

L390 The number of samples from mice is very impressive (n=875) and the density of information presented in the results is substantial.

Other Methods:
Two different extraction kits which can give varying fragment lengths etc, but that may not be as important for short-read seq, is this an issue?
Additionally, why localisation of ARGs as a proxy of ARG mobility? I think is fine but possibly for future research could maybe look at long-read seq of the metagenomes that contain many likely MGE-mediated ARGs, this might allow for some plasmid tracking amongst the samples.

Results
Is there a possible explanation for the finding that commensal gut bacteria like Enterobacteriaceae and Enterococcaceae were poorly represented in these samples, as that's fairly unusual I think!

(Remarks on code availability)

Reviewer #3

(Remarks to the Author)
Gicquel et al has completed an interesting and impressive study in which they examined the gut microbiomes and resistomes of house mice in the context of an ecological model to try to determine the ecological characteristics on the occurrence of antimicrobial resistance genes. They used metagenomic sequencing and a joint species distribution model to determine the influence of a variety of influencing factors. They first characterized the abundance and prevalence of the genes that were observed and describe that only a few of the genes identified were clinically relevant. The metagenomic sequences were not assembled to determine their mobility in the context of MAGs, but mobility was determined based on the gene's previous description of mobility in the Comprehensive Antibiotic Resistance Database (CARD). They then use the JSDM to determine that pig density was associated with the abundance of tetracycline resistance genes (as a category), sul2 and cblA-1.

While this study quantifies the ecological drivers of the mouse resistome (a potential vector of antibiotic resistance genes between environments), some of the claims are overstated or not well supported by the data presented in the paper. These weaknesses of the paper jeopardize its impact.

Major comments:
1. Many of the claims of the paper are overstated.
a. From the abstract (lines 58-59), "Environmental variables and livestock farming intensity explained more than 21% of ARG variation, whereas host characteristics accounted for less than 10%." However, only two host factors were considered, sex and body condition score, while there were 11 environmental and livestock farming intensity variables. All together in Fig. 3a those accounted for 27% of total ARG variation, not 21%. So this is an uneven comparison and the host factors may be inadequately represented.
b. From the abstract (lines 60-61), "Analysis of ARG traits revealed that agricultural land use and exposure to livestock increased the occurrence of mobile ARGs." This result comes from Fig. 4 and while this statement is true, it does not report the coefficient estimate (<2.5) or probability (75%). Livestock density only was associated with MGE with the pig-integron pair of data and the rest were not significant, or slightly negatively probable. Also the authors do not prove that any of these ARGs are mobile. They are stated in the CARD database that they are mobile, but they have no evidence of that from their own sequences. If MAGs had been assembled, and they examined the genetic context of these likely mobile ARGs, then they could have generated that evidence that they were mobile.
c. From the abstract (lines 62), "beta lactam resistance" was associated with pig density. According to Fig 4, the category beta lactam resistance is negatively predicted by pig density. Some beta lactam ARGs (Fig. S9) are associated (but only cblA-1 with confidence) with pig density, but not the entire category. There is also an issue with the format of gene names. Gene names should be italicized and lower case.
d. From Abstract (line 63), "mouse resistomes closely resembled those of livestock manure." It is assumed the authors are drawing this conclusion from Fig. 5b in that about half of the ARGs found in mice are also found in farm animals, and Fig. 5e-f shows that the most prevalent mouse ARGs are also found in farm animals. However, Fig 5c-d clearly show that taken as a whole, the mouse resistome is clearly distinct from the farm animal resistomes. So this conclusion is overstated. Additionally, these shared genes (mostly tet genes) are widespread genes and probably found in many environments.
Many of these same conclusions are repeated in the discussion and again may not be well supported by the evidence presented in this paper.
e. From Discussion (Line 670-671), "strongly influenced by land use characteristics". The authors should define what is a strong influence. This JSDM is not a universally common tool and the values generated by the model might be misunderstood by the reader. The only strong influence I see out of the model is that pig density is strongly related to sul2. Even the claim that pig density strongly influences ARGs in integrons is problematic to me because the authors did not demonstrate which genes were in integrons in their samples, but only associated with integrons in other studies. This relationship is better stated on line 527 "potential co-localization in integrons" and similar language should be used throughout the paper.
f. Similar issue was taken with statements in the discussion on lines 675-679.
2. The ARG traits are assumed from the literature and not demonstrated from the dataset. Both mobility and gene localization are assumed from other datasets and not demonstrated in this dataset. This is a serious weakness and has already been brought up in item #1 above.
3. Resistance conferring SNPs in housekeeping genes seem to be included in the dataset (Lines 393-394). These SNPs probably should have been excluded from the analysis. These SNPs have been confirmed and conferring resistance only in the species that they were described in (often E. coli, line 407). However, the SNPs in this dataset may have been identified from other bacterial species and may not confer resistance to those species. Thus, unless the identity of the host of this SNP can be identified, these SNPs should not be considered.
4. Fig 4. If I understand correctly, the same ARG could be included in multiple ARG trait categories. The gene sul1 was associated with pig farm density, but sul1 was included in the traits: Sulfonamides, antibiotic target replacement (Table S2), integron (line 528) and therefore, probably the mobility trait. Some of these categories only contain a few genes (Sulfonamides (3), antibiotic target replacement (6), integron(4)) and are therefore heavily influenced by sul1. So while it appears there are many effects due to pig farming, its likely due to sul1 and not the larger category as a whole (eg. Integrons)

Minor comments:
DNA extraction – use of two different extraction methods confounded within years may bias the chronological analysis of the

data. The effect of time was not considered often throughout the paper, but was considered in the overall description of the resistomes (Fig. 2).
Line 208 “45 b”. Should this be “45 bp”
Line 334 “read”. Should this be “road”
Line 391 “confers” should be “confer”
Line 785 It sounds like the authors are saying that sul1 is a beta lactamse. Rephrase.

(Remarks on code availability)
The github page was not active. Its probably set to private. The code should be set to public before consideration of publication. The data is also not available currently. The paper states, "All raw sequence data are deposited and available online at the European Nucleotide Archive with an accession number PRJEB102564." However, this accession number was not found on their website. These are both major issues that must be resolved before the peer review proceeds.

Version 1:

Reviewer comments:

Reviewer #1

(Remarks to the Author)
I am satisfied the authors have resolved my queries, and happy supporting publication of this interesting piece work.

(Remarks on code availability)
No comments.

Reviewer #2

(Remarks to the Author)
As stated in my 1st review, this is a really interesting paper examining AMR using gut samples from wild house mice (Mus musculus) from German farms with metagenomes.
Thank you to the authors for dealing with the queries I put forward regarding their paper.

There is one issue that needs to be addressed with a little more detail:
The details regarding the different extraction kit methods is still not sufficiently addressed in the paper, as results showed a significant difference. I appreciate that the effect size is small but the differences could be attributed to the DNA extraction. This needs to be noted more explicitly in the paper.

(Remarks on code availability)

Reviewer #3

(Remarks to the Author)
The authors have worked to address all reviewer comments. Some concerns remain, but the authors have done all they could to address the concerns. The work is a significant advancement for the research area.

(Remarks on code availability)

Reviewer #4

(Remarks to the Author)
I co-reviewed this manuscript with one of the reviewers who provided the listed reports. This is part of the Nature Communications initiative to facilitate training in peer review and to provide appropriate recognition for Early Career Researchers who co-review manuscripts.

(Remarks on code availability)

Open Access This Peer Review File is licensed under a Creative Commons Attribution 4.0 International License, which permits use, sharing, adaptation, distribution and reproduction in any medium or format, as long as you give appropriate credit to the original author(s) and the source, provide a link to the Creative Commons license, and indicate if changes were made.
In cases where reviewers are anonymous, credit should be given to 'Anonymous Referee' and the source.
The images or other third party material in this Peer Review File are included in the article's Creative Commons license, unless indicated otherwise in a credit line to the material. If material is not included in the article's Creative Commons license and your intended use is not permitted by statutory regulation or exceeds the permitted use, you will need to obtain permission directly from the copyright holder.
To view a copy of this license, visit https://creativecommons.org/licenses/by/4.0/

We thank the reviewers for their helpful comments on the preliminary version of our manuscript. In this document, we provide detailed answers to their questions and concerns.

We also include a revised version of our manuscript which includes the changes made highlighted and linked to the comment number and reviewer. We hope this helps to assess our implementation of the required changes.

Sincerely,
Víctor Hugo Jarquín-Díaz
In behalf of the authors

REVIEWER COMMENTS

Reviewer #1 (Remarks to the Author):

This is a very well-written and thorough examination of the gut resistomes of house mice from different locations in Germany. The study compares the composition of these resistomes to the environments in which the mice were captured, as well as to resistomes drawn from broader databases representing different farm animals from studies conducted in various parts of the world. The work is good and provides valuable insights into the relationships between environmental exposures, including exposure to different types of domesticated farm animals and the prevalence, types, and diversity of resistance genes acquired, and resistomes of nearby mice.

Assuming the assuming that defend my comments below, the resulting observations say a great deal about animal–animal transmission (not as much as many assume) and about how the broader environment surrounding animals influences resistance found in incidental species that are in proximity to other animals potentially exposed to antimicrobials. The authors identify particular associations between mice and pigs and show that herd density is an important factor, both of which are consistent with findings from other animal–animal systems. However, the work here is especially rigorous, and the data are more powerful than in previous studies. I therefore recommend publication with relatively minor revisions, assuming the authors can address my comments below.

Response: Thank you very much for the general assessment of our manuscript. We are happy that our study provides useful information into the field. We took all your comments into consideration and implemented the required changes. Detailed information for each of the comments are described below.

R1C1: My main concern with the paper relates to the title and how the data are discussed. This paper is fundamentally about farms, and the use of the term "anthropogenic" is misleading because the study does not examine urban or non-agricultural environments. As such, the title should be revised to something along the lines of:

“Anthropogenic land use exerts selection pressures on the resistome of non-domesticated rodents in farm environments” (or something like this)

Response: We agree that the title could be more focused in the main conclusions on the anthropogenic activities in farm environments, thus we revised our title:

Farming practices exerts selection pressures on the resistome of natural populations of house mice

R1C2: Having lived on a farm, I feel strongly that mice on farms are not truly "wild" but are instead part of the farm system. They are not domesticated rodents, but they experience substantial human–animal contact. Thus, describing them as "wild" is misleading. Moreover, the authors frequently use the term "house mice," which contradicts the use of "wild." Using the term "house mice" is appropriate, but "wild" is not. This is not merely a semantic issue: a truly wild rodent with limited domestic exposure (for example, in a savannah ecosystem) would, in theory, have a very different resistome, and this is not the system studied here.

Response: We agree that house mice *per se* are not truly representative of a wildlife species. In the original manuscript, we made the distinction and refer to them as *wild* since most of the time house mice (*Mus musculus*) are associated to be only a model for laboratory biomedical research, which is not the case for our sampled population. In the revised introduction and some parts of the discussion, we maintained the term "wild" to encompass any natural populations of non-domesticated animal hosts, which includes house mice. However, we also avoided the use of "wild" to refer specifically to the house mice (*Mus musculus*) used in our study. We adjust the term to "natural populations" in the title and throughout the manuscript when we refer to our study organism. In the methods at the "Sample collection" section, we added:

The mice from this study represent a natural population of non-domesticated animals, which co-habitat in domestic environments.

R1C3: Additionally, this study primarily examines farm systems, and the title is too general. As currently written, it implies that the work encompasses all environments with human impact, which is not the case. It would be clearer and more accurate for readers if "farm" or "agricultural" were included in the title and consistently stated throughout the manuscript. This change would not diminish the quality of the work but would instead present it more clearly in relation to what was actually studied.

Response: See response to ***R1C1***

Beyond these points, I have a few additional comments:

R1C4: 1. Figure 2 and associated discussion: The authors report differences between the periods 2016–2019 and 2019–2022, suggesting temporal changes. However, these periods also correspond to two distinct sampling campaigns. It is therefore possible that the observed differences reflect differences between campaigns rather than true temporal trends. Please examine potential differences in the sampling campaigns and justify the interpretation that these changes are time-related. Additionally, herd density may have changed over time and could explain the observed differences. Please discuss herd density in relation to time within the context of these data.

Response: We checked potential differences between the two sampling periods in terms of sequencing, taxonomic and ARGs detected. While we observed significant differences with a low effect size in sequencing depth between these two periods (Wilcoxon, r=0.096, *p*-value <0.001), the sampling period did not impact on the microbiome (Wilcoxon, r=0.031, *p*-value >0.05). We estimated the number of ARGs after rarefying to an even sequencing depth and still observed the increase in ARGs towards the end of the sampling period. We included these results in the revised manuscript in the Supplementary Figure S7 and Supplementary Table S8. We also adjusted the main text and legend of Fig. 2b to only specify time-related differences within the same one.

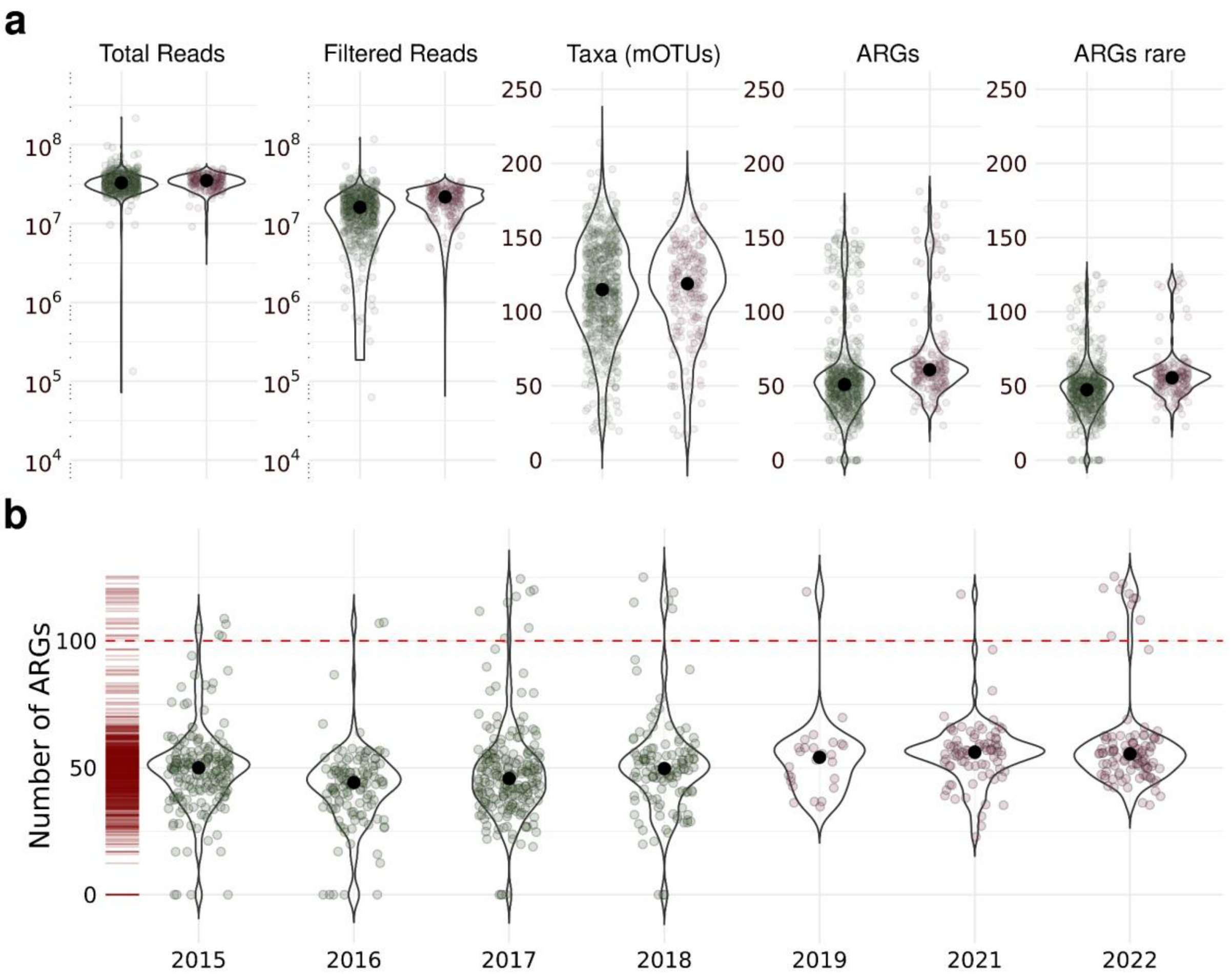

Supplementary Figure S7. (a) Effect of sampling campaigns in sequencing output, taxonomy and ARGs. Significant differences on total reads, filtered reads after preprocessing and number of ARGs without and with rarefaction were observed between sampling campaigns (green = 2015-2018, pink = 2019 to 2022), although the effect size of the difference in sequencing parameters was lower compared to the effect in ARG differences. **(b)** The number of rarefied ARGs detected in 2016 was significantly lower than the number detected in 2015 and 2018 within the same sampling campaign (Wilcoxon, *p*-value adjusted <0.001). There was no difference in ARGs detected between years at the end of the study period. Most mice (n = 843) had an ARG richness of around 50 genes, while a smaller group (n = 32) had a higher number of genes detected. The dashed red line defines a threshold of 100 ARGs detected. Each coloured point represents a single mouse metagenome. Black points indicate the median.

Supplementary Table S8. Effect of sampling campaigns in sequencing output, taxonomy and ARGs

Measurement	N Group 1	N Group 2	P-value	Significance	Effect size
Total reads	661	214	4.59E-03	**	0.096
Filtered reads	661	214	4.18E-17	****	0.284
Taxa (mOTUs)	645	214	0.223	NS	0.030
ARGs	661	214	2.23E-19	****	0.304
ARGs rarefied	661	214	2.26E-19	****	0.304

Regarding the animal density, we had used the averaged livestock units for cattle, pig and poultry per hectare based on the available data in 2020. The livestock density per year is only available for 2016 and 2020 within our sampling period, so we cannot test whether the number of ARGs detected by individuals varies across the entire sampling period due to changes in livestock density. However, we observed a strong correlation between the livestock density per municipality from both available years, which suggests an overall consistency. In addition, outliers suggest that pig and poultry densities had even been higher in some sampling sites in the year 2016, making our finding even more conservative and robust.

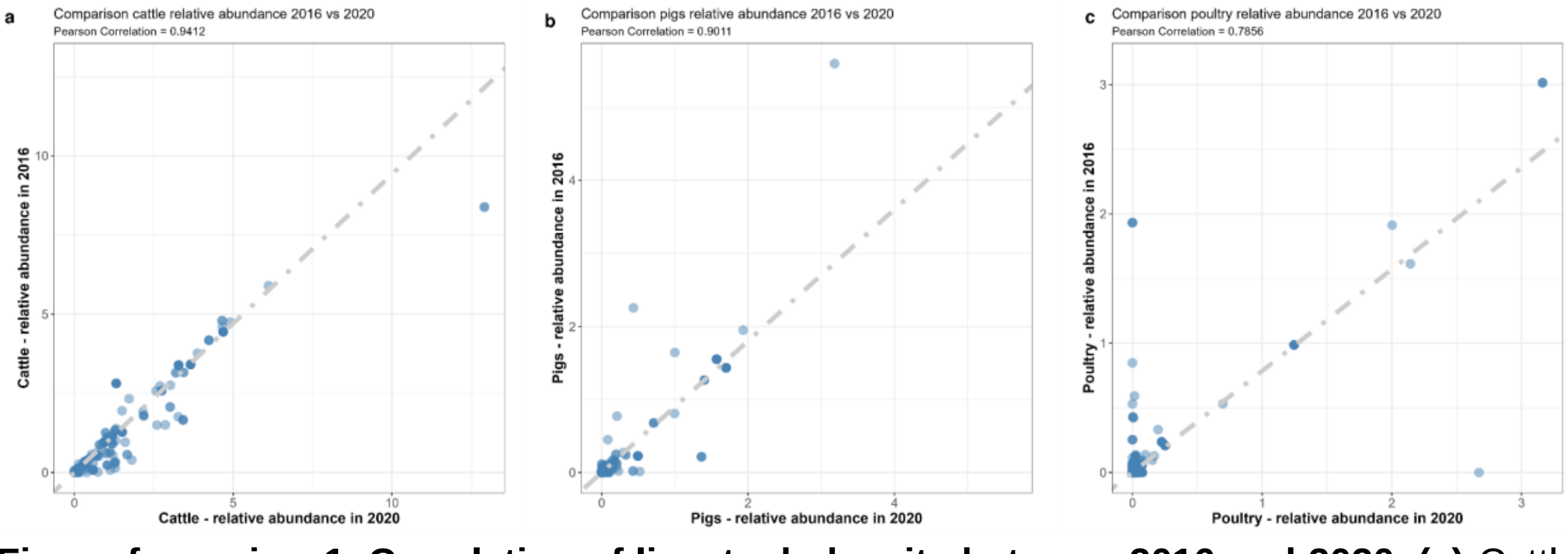

Figure for review 1. Correlation of livestock density between 2016 and 2020. (a) Cattle, **(b)** Pig, **(c)** Poultry.

R1C5: 2. Figure 3: Nothing in this figure indicates an urban context. This figure and its associated discussion exemplify the need for clearer editing to emphasize that this study focuses primarily on farm environments rather than urban settings.

Response: We adjusted any interpretation related to the environmental characteristics and specified in the text that those are related to the localities and geographical areas surrounding the farms where we capture the house mice. Despite this area being highly heterogeneous,

with farms being in densely populated municipalities with at least 50,000 inhabitants that are officially considered as urban areas according to the Federal Statistical Office. Thus some of those farms are in proximity to "urban areas", which might impact the surrounding characteristics. We only focus on discussing the impact of farming practices on the resistome of our natural populations of house mice.

R1C6: 3. Figure 5 (resistome diversity comparisons): Could the lower resistome diversity observed in mice, compared to other animals, be a consequence of differences in sampling and extraction methods? The mouse samples were collected and processed uniformly, whereas the comparison data were drawn from databases with no guarantee of comparable sampling depth, collection methods, or processing protocols. I am surprised that mouse resistomes appear to be relatively low in diversity; I would have expected them to resemble those of birds, which tend to be highly diverse. Please further justify the methods and interpretations here. It is possible that the observed low ARG diversity reflects bias, especially given that these mice were largely sampled from farms, which your own data suggest impose selective pressures related to herd density. That same selection pressure may also influence ARG diversity in mice.

Response: Technical differences between our dataset and those from livestock used for comparison were minimised by re-analysing and annotating ARGs the livestock dataset with the same strategy as for our mice dataset. Only next-generation sequencing metagenomic data from Illumina paired-end platform with 1E6 bases were used in the analysis to avoid potential issues arising from disparities in handling, collection or DNA extraction. All the preprocessing to filter out of low quality reads was performed under the same parameters. We observed that the ARG diversity in the livestock resistomes resembled those values reported in their corresponding publications.

R1C7: 4. Pages 698–705: Could the observed patterns be influenced by sampling limitations? The methods used to collect and analyze the mice may reflect the inherently less diverse environments associated with farm ecosystems, which may select for lower diversity overall. This further underscores the importance of removing references to "wild" throughout the manuscript. Please consider this point carefully in the broader narrative.

Response:

We focused our sample collection in a way to represent the microbial composition from the house mice GI tract and minimise the impact or cross-contamination of any environmentally derived bacteria (soil, water). We consider that lower overall ARG diversity is mostly associated to the different degree of exposure rather than to collection-derived factors, as reflected by sampling multiple years and different localities. Our results actually confirm that the composition of genes detected heavily depend on the locality, but in general house mice are not as rich as other sources (farm animals, for example).

In discussion:

While the natural populations of house mice are neither fully "wild" nor fully "domestic" animals, they represent an intermediate stage that gets in contact and influenced by both environments. Thus, the house mouse ARG composition was largely shaped by the bacterial taxa present in

their microbiomes, and both bacterial and ARG composition could reflect the degree of anthropogenic exposure and potential for gene exchange across ecological boundaries.

Reviewer #2 (Remarks to the Author):

This is a really interesting paper examining AMR using gut samples from wild house mice (Mus musculus) from German farms with metagenomes. The data were analyses using a range of techniques, but the traits revealed that agricultural land use and exposure to livestock increased the occurrence of mobile antimicrobial resistance genes. This is a well
well-designed with important findings as to where the source of AMR in wildlife derives. The statistical analyses linking the host and environmental variables is really interesting. Other comments are outlined below.

Response: We appreciate your general assessment of the manuscript and we are pleased to see your interest in the results and the analysis. We respond to your valuable comments in more detail below.

R2C1: L54 Are the factors know any better in non-human dominated landscapes? In Europe, even 'wild' systems are all human dominated.

Response: Actually it applies to both species inhabiting human and non human impacted areas, this stresses the point on how little we know of ARG maintenance in wildlife microbiomes. We include a reference to this fact in the revised abstract:

Antimicrobial resistance poses a significant global health challenge. However, the factors maintaining antimicrobial resistance genes (ARGs) in non-domesticated animal microbiomes remain unclear particularly for species inhabiting human-dominated or less human-impacted landscapes.

R2C2: L103-104 And in the Introduction in general- human activity is driving this process so don't frame it as wildlife being the source of the issue or blame for the problem. In order to be accurate, soften the language used regarding the role of AMR risk to public health.

Response: We actually intended to emphasise that there is a lack of understanding on what is the role of wildlife species in the maintenance and transmission of ARGs. We adjusted parts of the introduction to make this point clear and avoid any point that could suggest wildlife species as responsible for AMR:

Metagenomic screening has extensively characterised the resistome of human gut, wastewater and livestock[19–22]. Such high throughput approaches have been instrumental to identifying socio-economic, genetic and environmental factors associated with resistance potential, and the dynamics of clinically relevant ARGs from environmental reservoirs to humans[10,23–26]. *However, the sporadic systematic metagenomic screening of wildlife-associated resistomes limits our understanding of their role in AMR dynamics and background levels of resistance in species able to mobilise antimicrobial resistant bacteria over large geographical distances or to maintain local dissemination*[27,28]. Therefore, the use of high-throughput approaches in wildlife species becomes crucial in two directions: first, to determine

the basal resistome in their microbiomes and second to assess the impact of environmental drivers in resistance dynamics.

R2C3: L130 Animals found in human dominated landscapes make us sick in respect to zoonoses
This citation may be useful for L112-130:
Gibb, R., Redding, D.W., Chin, K.Q. et al. Zoonotic host diversity increases in human-dominated ecosystems. Nature 584, 398–402 (2020). https://doi.org/10.1038/s41586-020-2562-8

Response: This reference was added into the manuscript. It indeed clearly represents the point of animals in human areas to be essential in infectious diseases ecology.

R2C4: L132-144: Although the content in this section is broadly accurate is it poorly referenced. Please see below some ideas for zoonoses emergence. I believe that your data is perfectly placed to show an analogous situation for AMR linked to zoonoses but the framing needs to be correct. It was first the case with zoonoses but now you're demonstrating a similar link with AMR.
https://doi.org/10.1073/pnas.1208059110
https://doi.org/10.1111/zph.12489
https://doi.org/10.1016/S2542-5196(21)00031-0

Response: These references were added into the manuscript.

R2C5: L319 What were the random and fixed effects? Could you include a Table that outlines that data and categories attributes e.g. response or explanatory, nature of data, fixed/random

Response: We apologise to not clarify this very well in the text. In the revised version variables included in the models are described in the following paragraph.

R2C6: L390 The number of samples from mice is very impressive (n=875) and the density of information presented in the results is substantial.

Response: Thank you for highlighting our dataset.

Other Methods:
R2C7: Two different extraction kits which can give varying fragment lengths etc, but that may not be as important for short-read seq, is this an issue?

Response: Thank you very much for raising this concern. Indeed extraction protocols might introduce differences. This comment also relates to ***R1C4***. We checked potential differences between the two sampling periods in terms of sequencing, taxonomic and ARGs detected and we observed significant differences with a low effect size in sequencing depth between these two periods (Wilcoxon, r=0.096, *p*-value <0.001), the sampling period did not impact on the microbiome (Wilcoxon, r=0.031, *p*-value >0.05). We estimated the number of ARGs after rarefying to an even sequencing depth and still observed the increase in ARGs towards the end of the sampling period. We include this analysis in the results and supplementary material.

R2C8: Additionally, why localisation of ARGs as a proxy of ARG mobility? I think is fine but possibly for future research could maybe look at long-read seq of the metagenomes that contain many likely MGE-mediated ARGs, this might allow for some plasmid tracking amongst the samples.

Response: We use a broad approximation of the actual gene mobility to localise the genes, which helps us to overcome the limitations of short-read MGE coverage. In the revised manuscript, we present an assembly-based approach to analyse plasmids in our house mice and determine whether they co-localise with ARGs. We include those results at the revised manuscript and as Supplementary Figure S9 (*see also **R3C12***). However, as some of the genes are present in low abundance and prevalence, not all of the ARGs detected were co-localised in plasmids. A deeper shotgun sequencing dataset aims to track more MGEs in these metagenomes, which is beyond the scope of this manuscript.

Results
R2C9: Is there a possible explanation for the finding that commensal gut bacteria like Enterobacteriaceae and Enterococcaceae were poorly represented in these samples, as that's fairly unusual I think!

Response: While members of the Enterobacteriaceae and Enterococcaceae families are recognised as commensals of the animal microbiome and therefore used as a marker of fecal pollution in food or water, based on our own previous work and that of others (Cizkova et al., 2023; Ferreira et al., 2024, Hanski et al., 2025), mainly carried out using 16S amplicon sequencing, we have observed that natural populations of house mice are not enriched in these more common families. Most likely related to their diet in natural conditions, other bacteria groups like Lachnospiraceae which are fiber-degradators are much more abundant (Carmody et al. 2015). We include this explanation at the discussion:

Families of commensal gut bacteria such as *Enterobacteriaceae*, *Enterococcaceae*, and *Staphylococcaceae*, which are usually involved in mediating ARG transmission within host-associated microbiomes[22,60,61], were poorly represented in our house mouse samples *as previously reported with amplicon-based approaches (Cizkova et al., 2023; Ferreira et al., 2024, Hanski et al., 2025), and mostly dominated by known fiber degraders like members of Lachnospiracea family probably related to their diet (Carmody et al. 2015).*

Reviewer #3 (Remarks to the Author):

R3C1: Gicquel et al has completed an interesting and impressive study in which they examined the gut microbiomes and resistomes of house mice in the context of an ecological model to try to determine the ecological characteristics on the occurrence of antimicrobial resistance genes. They used metagenomic sequencing and a joint species distribution model to determine the influence of a variety of influencing factors. They first characterized the abundance and prevalence of the genes that were observed and describe that only a few of the genes identified were clinically relevant. The metagenomic sequences were not assembled to determine their mobility in the context of MAGs, but mobility was determined based on the gene's previous description of mobility in the Comprehensive Antibiotic Resistance Database (CARD). They then use the JSDM to determine that pig density was

associated with the abundance of tetracycline resistance genes (as a category), sul2 and cblA-1.

R3C2: While this study quantifies the ecological drivers of the mouse resistome (a potential vector of antibiotic resistance genes between environments), some of the claims are overstated or not well supported by the data presented in the paper. These weaknesses of the paper jeopardize its impact.

Response: Thank you for your general assessment of the manuscript and for highlighting relevant points. We have taken all of these into consideration and responded to your comments in more detail below. We have ensured that all claims are supported by the presented data, and any speculative claims have been toned down. We hope that the revised version clarifies the main message and conclusions more effectively.

Major comments:

R3C3: 1. Many of the claims of the paper are overstated.
a. From the abstract (lines 58-59), “Environmental variables and livestock farming intensity explained more than 21% of ARG variation, whereas host characteristics accounted for less than 10%.” However, only two host factors were considered, sex and body condition score, while there were 11 environmental and livestock farming intensity variables. All together in Fig. 3a those accounted for 27% of total ARG variation, not 21%. So this is an uneven comparison and the host factors may be inadequately represented.

Response: We corrected the values in the abstract to correspond to the variance actually explained by the environmental and host factors in the model, respectively, without making a direct comparison. We agree that host factors may not be represented equally alongside the environmental factor. However, we have no further host characteristics at the same level of granularity as the environmental ones. In the revised version, we have included an analysis of a subset of mice (n = 771), for which we have calculated the genotype as a Hybrid Index (HI), as reported in Balard et al. (2020). Models including the genotype increased the variance explained by mouse characteristics from 3% to 9%. We also observed relative increases in the variance explained by landscape variables (land cover, accessibility and farm animals) and climatic variables, from 27% to 37% in the model for all ARGs, and from 41% to 46% in the model with mobile ARGs (see Supplement Figure 14). These additional results suggest that the HI reduces the unexplained variance (random effects) and enhances the importance of environmental factors.

We provide further clarification of the interpretation of variance explained by models. In general, it is possible to compare the variance explained by variables that are equally represented in the models, e.g. between different groups of ARGs (total vs mobile) or different traits, as the groups of variables are the same in both cases. In these situations, an increase or decrease in variance really means that a group of variables is more or less important for that group or trait. We elaborate about this in the discussion:

In discussion:

We observed that intrinsic characteristics of each farm exerted a strong influence on the overall ARG composition. Ecological and environmental variables also explained a substantial proportion of the variability in ARG occurrence. When the environmental effects are taken together, they exceed the effects of host density and sex, consistent with previous observations in the human gut resistome[19]. While our models included more environmental than host variables and therefore a higher amount of variance was expected, each subset of environmental variables (e.g. land cover, accessibility and farm animals) had a similar explanatory power to host variables with regard to the total ARG composition. This highlights the importance of the environment and its different components. Furthermore, this importance increased drastically for mobile ARGs. These patterns likely reflect anthropogenic factors that promote antibiotic resistance in bacteria within the mouse gut. Environmental and climatic factors were more influential for genes potentially co-localised on mobile genetic elements and therefore more likely to be transferred through horizontal gene transfer.

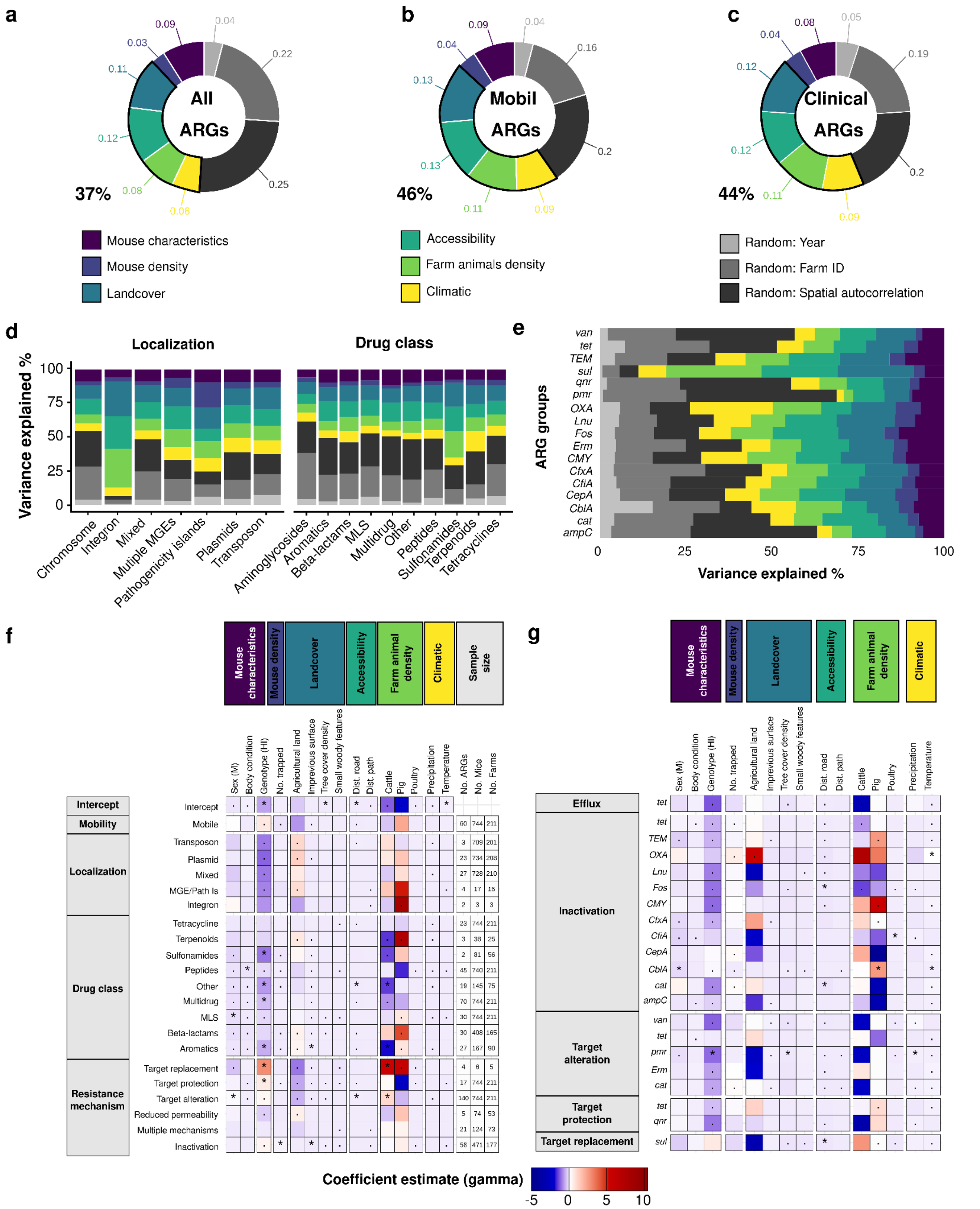

Supplementary Figure S14. Variance partitioning showing the relative contribution of mouse characteristics, environmental factors, and random effects, on the explained variation in all **(a)** ARGs detected, **(b)** potentially mobile ARGs and **(c)** clinically relevant ARG occurrence in mouse gut microbiomes in a model that include host genotype (see Balard et al. 2020 and Ferreira et al., 2024 for details) as an additional host characteristic. **(d)** The variance partitioning averaged for each ARG's traits shows the occurrence of genes with potential localization in integrons, genes conferring resistance to sulfonamides and conferring resistance to sulfonamides are more influenced by environmental factors. **(e)** Variance partitioning in occurrence averaged for each clinically relevant ARG group. For *sul* genes

landscape characteristics are particularly relevant and account for more than 70% of occurrence variance. The heatmaps show the coefficient estimate (gamma) for the ARG traits **(f)** or for each clinically relevant ARG group and a given explanatory variable of the jSDM **(g)**. The higher the coefficient estimate, the higher the association of the variable with the ARG group. Symbol in tile indicate the posterior probability of the estimate in the model: ∗ > 0.95 and · > 0.75.

R3C4: b. From the abstract (lines 60-61), "Analysis of ARG traits revealed that agricultural land use and exposure to livestock increased the occurrence of mobile ARGs." This result comes from Fig. 4 and while this statement is true, it does not report the coefficient estimate (<2.5) or probability (75%). Livestock density only was associated with MGE with the pig-integron pair of data and the rest were not significant, or slightly negatively probable. Also the authors do not prove that any of these ARGs are mobile. They are stated in the CARD database that they are mobile, but they have no evidence of that from their own sequences. If MAGs had been assembled, and they examined the genetic context of these likely mobile ARGs, then they could have generated that evidence that they were mobile.

Response: In the revised version of the abstract, we included the posterior probability for the findings related to the mobile ARGs. Regarding the mobility of the genes, we used the reported mobility from CARD as reference and now referred to it as potential mobility in the revised version. We performed an assembly-based annotation and given that some of the antimicrobial resistance genes in the house mice were identified with low-abundance within the complex gut community, some of them had incomplete or poor assemblies which makes it more difficult to assess their co-localisation within MGE. However, for some of the genes found in large contigs, we were able to report their genetic context. We added this information in the methods and the results (Supplementary Figure S9).

In methods:

The potential mobility of the ARGs detected by a read-based approach was determined based on their genetic locations with MGEs by referencing CARD Data. To determine the genetic context of ARGs, a contig-based annotation was performed. Metagenomic de novo assembly was performed with the decontaminated and quality controlled reads using SPAdes genome assembler v4.1.0 under default parameters (k-mer= 21, 29, 39, 59, 79, 99). Only contigs longer than 1000b were used for further analysis. ARG annotation was done with RGI main using DIAMOND aligner and to plasmids using Plasmer v1.0 (Zhu et al. 2023). Only ARGs in contigs with identity and coverage to the reference equal or higher to 80% were considered.

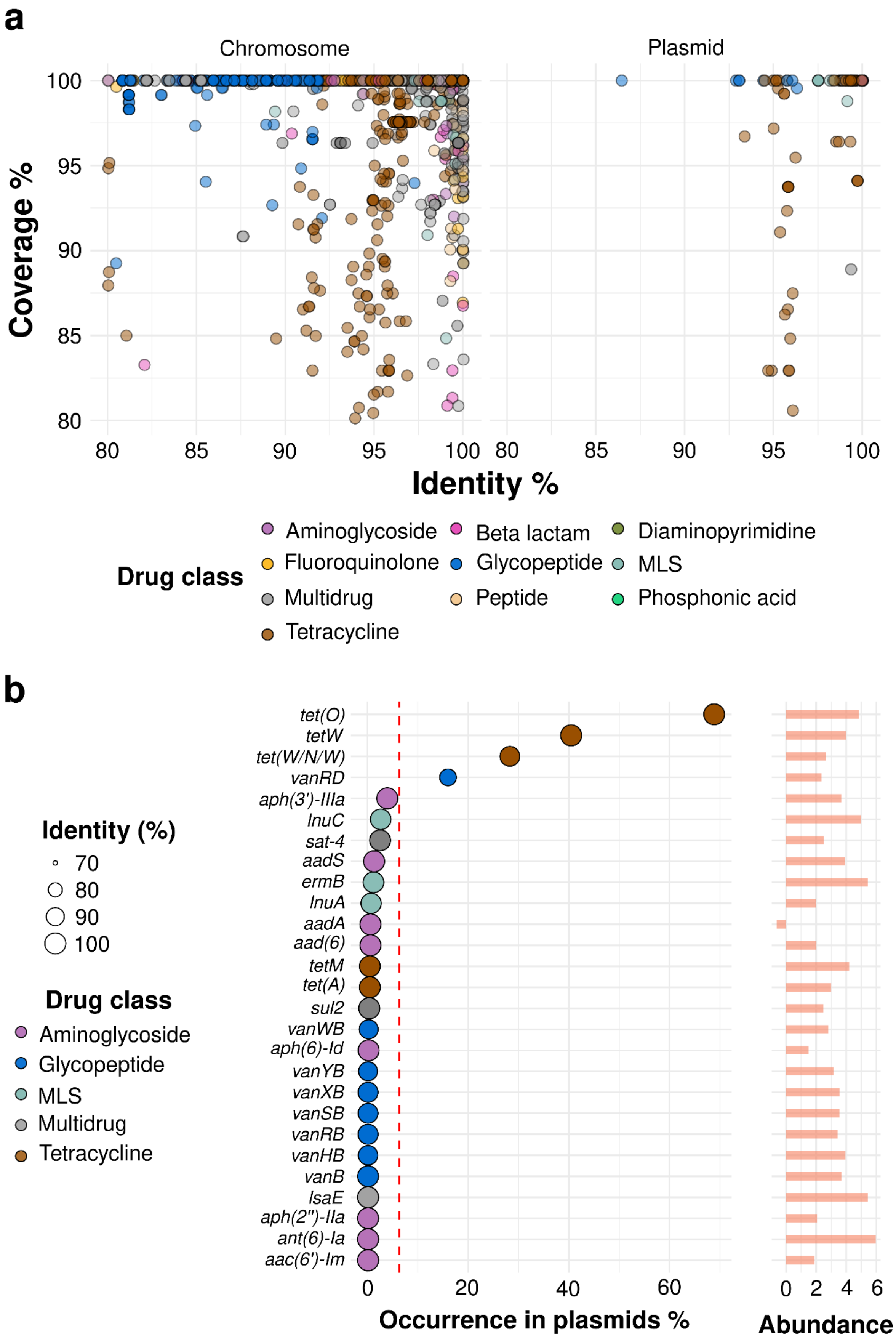

Supplementary Figure S9. Genetic context of ARGs in contigs with more than 1000b. **(a)** Out of 5874 contigs containing genes with >=80% identity and >=80% coverage, 96% of these

contigs correspond to genes in bacterial main chromosomes (N= 5687) and 235 contigs correspond to genes localised in plasmids. **(b)** Plasmids carry 27 different ARGs, three *tet* genes co-localised at plasmids were detected in more than 20% of samples, the rest of genes were detected in plasmids for less than 10% of the samples. The mean abundance was 15 FPKM across the plasmids. The size of the points represent the percentage of identity to the reference ARG- The abundance is expressed as log2 FPKM.

R3C5: c. From the abstract (lines 62), "beta lactam resistance" was associated with pig density. According to Fig 4, the category beta lactam resistance is negatively predicted by pig density. Some beta lactam ARGs (Fig. S9) are associated (but only cblA-1 with confidence) with pig density, but not the entire category. There is also an issue with the format of gene names. Gene names should be italicized and lower case.

Response: We adjusted the phrasing both in the abstract and throughout the text to specify that only the gene *cblA-1* was the only one conferring resistance to beta lactams associated with confidence to pig density. We also corrected the format of the gene names in the supplementary figure and in the text.

R3C6: d. From Abstract (line 63), "mouse resistomes closely resembled those of livestock manure." It is assumed the authors are drawing this conclusion from Fig. 5b in that about half of the ARGs found in mice are also found in farm animals, and Fig. 5e-f shows that the most prevalent mouse ARGs are also found in farm animals. However, Fig 5c-d clearly show that taken as a whole, the mouse resistome is clearly distinct from the farm animal resistomes. So this conclusion is overstated. Additionally, these shared genes (mostly tet genes) are widespread genes and probably found in many environments.
Many of these same conclusions are repeated in the discussion and again may not be well supported by the evidence presented in this paper.

Response: In the original manuscript, we specified in the results that the house mice resistomes have a distinctive ARG composition. We now avoid overstated conclusions and clearly indicate in the abstract and discussion that around 50% of the genes are shared with livestock resistomes and specify that this fraction includes genes widely spread and also some genes which are more prevalent in livestock's resistomes.

In abstract:

Consistent with these findings, mouse resistomes have a distinctive resistome, but share more than 50% of the genes with those in livestock manure, including widespread genes and those promoted in livestock.

In results:

The house mouse resistome showed a clearly distinctive ARG composition compared to the resistomes of livestock manure (PERMANOVA, R2= 0.21, p-value <0.01, Figure 5c). However, the resistome similarity was higher between resistomes of mice and pig and cattle manure than between resistomes from poultry hosts (contrast pig-mouse: -0.264, SE [0.0098], contrast cattle-mouse: -0.310, SE [0.013], Figure 5d).

R3C7: e. From Discussion (Line 670-671), “strongly influenced by land use characteristics”. The authors should define what is a strong influence. This JSDM is not a universally common tool and the values generated by the model might be misunderstood by the reader. The only strong influence I see out of the model is that pig density is strongly related to sul2. Even the claim that pig density strongly influences ARGs in integrons is problematic to me because the authors did not demonstrate which genes were in integrons in their samples, but only associated with integrons in other studies. This relationship is better stated on line 527 “potential co-localization in integrons” and similar language should be used throughout the paper.

Response: While we specified that the overall effect of the explanatory variable from the jSDM is determined by the proportion of variance explained by the model at lines 351–353 of the original manuscript, we did not indicate the reference comparison to show that some of them “strongly” at lines 670–671. For this reason, we have avoided using adjectives to describe the jSDM results in the revised version without clearly stating the reference for a specific comparison, as pointed out in ***R3C3*** and ***R3C8***. We also adjusted the conclusion to indicate clearly that the effect is based on potential co-localisation in mobile genetic elements.

R3C8: f. Similar issue was taken with statements in the discussion on lines 675-679.

Response: In the revised version, we adjusted this conclusion to the results reported in our analysis.

We demonstrated that environmental variables, particularly livestock farming intensity, explained a higher proportion of the variation in ARG occurrence than host characteristics did. This proportion increased further when the analysis was restricted to genes potential co-localisation in mobile genetic elements. High pig farming density was closely associated with the integron-encoded sulfonamide resistance gene sul1, as well as with genes conferring resistance to tetracyclines and one to beta-lactams.

R3C9: 2. The ARG traits are assumed from the literature and not demonstrated from the dataset. Both mobility and gene localization are assumed from other datasets and not demonstrated in this dataset. This is a serious weakness and has already been brought up in item #1 above.

Response: *See response to* ***R3C4***

R3C10: 3. Resistance conferring SNPs in housekeeping genes seem to be included in the dataset (Lines 393-394). These SNPs probably should have been excluded from the analysis. These SNPs have been confirmed and conferring resistance only in the species that they were described in (often E. coli, line 407). However, the SNPs in this dataset may have been identified from other bacterial species and may not confer resistance to those species. Thus, unless the identity of the host of this SNP can be identified, these SNPs should not be considered.

Response: Considering the general low abundance of *E. coli* in the house mice from our study, we are not able to generate *E. coli* MAGs and thus report a direct association between

these SNPs and *E. coli*. However, we observed that mice with higher ARG richness were generally associated with higher *E. coli* abundance (Supplementary Figure S7c). In the revised version, we checked with more detail which ARGs characterise the *E.coli* enriched group by differential abundance analysis. These results show that *E. coli* point mutations characterise the resistome in mice with higher *E. coli* abundance (Supplementary Figure SX). For this reason, we decided to still include them in the main analysis. We have also adjusted the manuscript to clarify this point and indicate that our results are consistent only for clinically relevant genes.

In result:

The genes corresponding to mutations in housekeeping genes associated with Escherichia coli variants (n=48) characterise the resistome from mice with high ARG richness (>=100 ARGs) as they are occurring more and differentially abundant (Supplementary Figure S11).

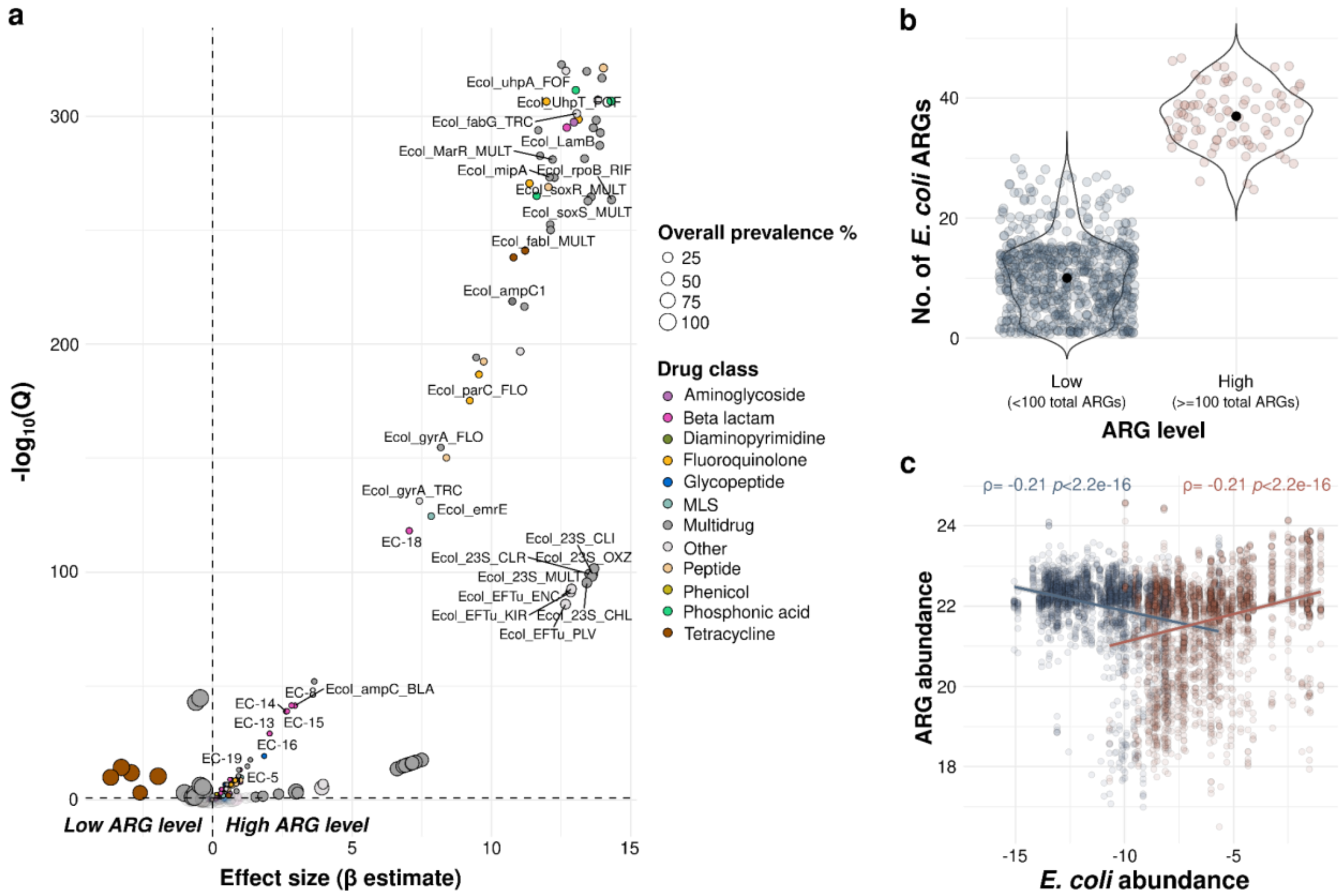

Supplementary Figure S11. Mice with high ARG level (>= 100 ARGs) are characterised by *Escherichia coli* related genes. **(a)** 48 genes related to *E. coli* were differentially abundant and **(b)** significantly higher in occurrence in mice with higher ARG richness. The volcano plot shows the genes' different abundance between the two subsets of house mice metagenomes. The size of the points indicates its prevalence across metagenomes and color the drug class. Violin plot shows that mice with higher ARG richness carry significantly more resistance conferring SNPs in housekeeping genes (Wilcoxon, r=0.52, *p*-value <0.001). **(c)** The abundance of those *E. coli* related genes was positively correlated to *E. coli* abundance in the house mice metagenomes. Spearman's correlation between *E. coli* and ARGs. The abundance for both *E. coli* and ARGs is expressed as log2 relative abundance or FPKM, respectively.

R3C11: 4. Fig 4. If I understand correctly, the same ARG could be included in multiple ARG trait categories. The gene sul1 was associated with pig farm density, but sul1 was included in the traits: Sulfonamides, antibiotic target replacement (Table S2), integron (line 528) and therefore, probably the mobility trait. Some of these categories only contain a few genes (Sulfonamides (3), antibiotic target replacement (6), integron(4)) and are therefore heavily influenced by sul1. So while it appears there are many effects due to pig farming, its likely due to sul1 and not the larger category as a whole (eg. Integrons)

Response: Yes, each ARG trait includes multiple genes, and each gene can be included in multiple traits. We clarify this point in the text to make it clear.

In the methods:

To assess how ARG trait–host-environment interactions shaped ARGs distributions, we also included a trait matrix comprising relevant ARG characteristics (see ARG traits and explanatory variables) in both models. Each of the detected ARGs in house mice resistomes could be represented in multiple traits.

Minor comments:

R3C12: DNA extraction – use of two different extraction methods confounded within years may bias the chronological analysis of the data. The effect of time was not considered often throughout the paper, but was considered in the overall description of the resistomes (Fig. 2).

Response: Thank you very much for raising this concern. Indeed extraction protocols might introduce differences. This comment also relates to ***R1C4*** and ***R2C7***. We checked potential differences between the two extraction methods in terms of sequencing, taxonomic and ARGs detected. While we observed significant differences in sequencing depth between these two periods with a low effect size (Wilcoxon, r=0.096, *p*-value <0.001), the sampling period did not impact on the microbiome (Wilcoxon, r=0.031, *p*-value >0.05). We confirmed the temporal changes in ARGs were detected independently from the two different extraction methods.

R3C13: Line 208 “45 b”. Should this be “45 bp”

Response: corrected

R3C14: Line 334 “read”. Should this be “road”

Response: corrected

R3C15: Line 391 “confers” should be “confer”

Response: corrected

R3C16: Line 785 It sounds like the authors are saying that sul1 is a beta lactamse. Rephrase.

Response: All the minor comments were implemented in the revised version. Thank you for pointing them out.

Reviewer #3 (Remarks on code availability):

R3C17: The github page was not active. Its probably set to private. The code should be set to public before consideration of publication. The data is also not available currently. The paper states, "All raw sequence data are deposited and available online at the European Nucleotide Archive with an accession number PRJEB102564." However, this accession number was not found on their website. These are both major issues that must be resolved before the peer review proceeds.

Response: We apologise for not making both the repository and sequencing data available since the first submission. We should have provided access to reviewers. The data and the scripts for the analysis are now available at the European Nucleotide Archive with an accession number PRJEB102564 (https://www.ebi.ac.uk/ena/browser/view/PRJEB102564) and https://github.com/mgicquel/mus_musculus_de_amr, respectively.

Thanks to the reviewers for the final remarks on our manuscript. We provide the answers to the pending questions.

Sincerely,

Víctor Hugo Jarquín-Díaz
In behalf of the authors

REVIEWERS' COMMENTS
Reviewer #1 (Remarks to the Author):

I am satisfied the authors have resolved my queries, and happy supporting publication of this interesting piece work.

Reviewer #1 (Remarks on code availability):

No comments.

Response: We are pleased that all the adjustments and clarifications besides improving our manuscript, they satisfied your queries. Thanks for your support and helpful comments.

Reviewer #2 (Remarks to the Author):

As stated in my 1st review, this is a really interesting paper examining AMR using gut samples from wild house mice (Mus musculus) from German farms with metagenomes. Thank you to the authors for dealing with the queries I put forward regarding their paper.

There is one issue that needs to be addressed with a little more detail:
The details regarding the different extraction kit methods are still not sufficiently addressed in the paper, as results showed a significant difference. I appreciate that the effect size is small but the differences could be attributed to the DNA extraction. This needs to be noted more explicitly in the paper.

Response:

Overall, thanks for your support and helpful comments that improved the manuscript.

Thank you very much for the remark regarding the different extraction methods. In the final version of the manuscript, we explicitly indicate that differences in sequencing depth could be attributed to the different DNA extraction methods, but the effect remains to be overall low.

In Results
While temporal differences could reflect small differences in sequencing depth between sampling periods (Two-sided Wilcoxon rank-sum test, effect size r=0.096, p-value =4.59E-03, Supplementary Table 1) that could be attributed to the different DNA extraction kits, this change did not impact the microbial richness and the increase in ARGs was detected nonetheless after rarefying to even sequencing depth (Supplementary Fig. 2 and Table S3).

Reviewer #3 (Remarks to the Author):

The authors have worked to address all reviewer comments. Some concerns remain, but the authors have done all they could to address the concerns. The work is a significant advancement for the research area.

Response: We appreciate all the helpful comments that improved the manuscript. We are happy that despite the remaining concerns you find the results and conclusion of relevance in the field.

Reviewer #4 (Remarks to the Author):

I co-reviewed this manuscript with one of the reviewers who provided the listed reports. This is part of the Nature Communications initiative to facilitate training in peer review and to provide appropriate recognition for Early Career Researchers who co-review manuscripts.

Response: Thank you for your support in reviewing the manuscript.
